# Bioinformatics and *in vitro* experimental analyses identify the selective therapeutic potential of interferon gamma and apigenin against cervical squamous cell carcinoma and adenocarcinoma

**DOI:** 10.18632/oncotarget.17574

**Published:** 2017-05-02

**Authors:** Pei-Ming Yang, Chia-Jung Chou, Ssu-Hsueh Tseng, Chien-Fu Hung

**Affiliations:** ^1^ Department of Pathology, Johns Hopkins Medical Institutions, Baltimore, MD, United States; ^2^ Department of Oncology, Johns Hopkins Medical Institutions, Baltimore, MD, United States; ^3^ Graduate Institute of Cancer Biology and Drug Discovery, College of Medical Science and Technology, Taipei Medical University, Taipei, Taiwan

**Keywords:** cell cycle, cervical cancer, drug repurposing, flavonoid, interferon

## Abstract

The clinical management and treatment of cervical cancer, one of the most commonly diagnosed cancers and a leading cause of cancer-related female death, remains a huge challenge for researchers and health professionals. Cervical cancer can be categorized into two major subtypes: common squamous cell carcinoma (SCC) and adenocarcinoma (AC). Although it is a relatively rare histological subtype of cervical cancer, there has been a steady increase in the incidences of AC. Therefore, new strategies to treat cervical cancer are urgently needed. In this study, the potential uses of IFNγ-based therapy for cervical cancer were evaluated using bioinformatics approaches. Gene expression profiling identified that cell cycle dysregulation was a major hallmark of cervical cancer including SCC and AC subtypes, and was associated with poor clinical outcomes for cervical cancer patients. *In silico* and *in vitro* experimental analyses demonstrated that IFNγ treatment could reverse the cervical cancer hallmark and induce cell cycle arrest and apoptosis. Furthermore, we demonstrated that apigenin could enhance the anticancer activity of IFNγ in a HeLa cervical AC cell line by targeting cyclin-dependent kinase 1. Taken together, the present study suggests the selective therapeutic potential of IFNγ alone or in combination with apigenin for managing cervical SCC and AC.

## INTRODUCTION

Persistent infection of high-risk human papillo-mavirus (HPV) has been identified as the main etiological factor for cervical cancer [1]. HPV oncogenes E6 and E7 are involved in the transformation and immortalization of cervical cells, and are required for malignant progression [1]. E6 and E7 regulate tumor growth by forming complexes with tumor suppressor proteins (p53 and pRb, respectively) and inhibiting their function [2]. To date, the standard therapeutic strategy for treatment of cervical cancer involves a combination of surgery, chemotherapy or radiotherapy. Furthermore, effective therapeutic treatments for patients with metastatic or recurrent cervical cancer after platinum-based chemotherapy are still lacking [3]. In addition, the adverse effects of current chemotherapy regimens remain a major concern. Therefore, the develop-ment of alternative therapeutic drugs or combination treatments is urgently needed to improve patient survival and reduce morbidity rates associated with standard of care therapies.

Precancerous lesions of the cervix typically develop into cervical cancer over 10 to 20 years. The major histologic types of human cervical cancer are squamous cell carcinoma (SCC, 80-85%) and adenocarcinoma (AC, 15-20%) [4]. Although the incidence of SCC has had a marked decline due to cytologic screening, there has been a rise in the incidences of AC [4]. Currently, there is no difference in the treatment strategy between SCC and AC; however, they respond very differently to treatment [4]. Because patients with AC have a worse prognosis and lower survival rate than those with SCC [5–8], it has been postulated that new treatment strategies specifically tailored to AC should be explored [4].

Interferon gamma (IFNγ), a pleiotropic cytokine mainly produced by T lymphocytes and natural killer (NK) cells, is essential for the function of immune cells, and innate and adaptive immune responses [9]. IFNγ has been used in a wide variety of clinical indications including cancers. The anticancer activity of IFNγ is associated with its antiproliferative, antiangiogenic, and proapoptotic effects [9]. However, IFNγ can also exhibit protumor activity involving proliferative and antiapoptotic signaling [10]. Thus, the outcome of IFNγ cancer therapy may depend on the cellular, microenvironmental, and molecular contexts [10]. A better understanding of the action mechanisms of IFNγ is important in order to create novel strategies to potentiate its anticancer activity.

In this study, IFNγ was found to be a potential anticancer agent against both SCC (SiHa) and AC (HeLa) subtypes of the cervix. Bioinformatics methods based on gene expression profiling were used to study the molecular mechanism for the anticancer activity of IFNγ and design a new strategy to enhance its efficacy. We found that cell cycle dysregulation was the common hallmark of cervical SCC and AC, and could be reversed by IFNγ treatment. In addition, Connectivity Map (CMap) analysis identified that apigenin, a dietary flavonoid, may enhance the anticancer activity of IFNγ by targeting cyclin-dependent kinase 1 (CDK1). *In vitro* experimental analyses demonstrated that combination treatment with apigenin enhanced IFNγ-induced cytotoxicity through the increases of cell cycle arrest and apoptosis in HeLa (AC subtype) but not SiHa (SCC subtype) cells. Therefore, we demonstrate that IFNγ alone or in combination with apigenin is a selective therapeutic strategy for managing different histological subtypes of cervical cancer.

## RESULTS

### IFNγ exhibits anticancer activity toward cervical SCC and AC cells through induction of cell cycle arrest and apoptosis

**Figure 1:**
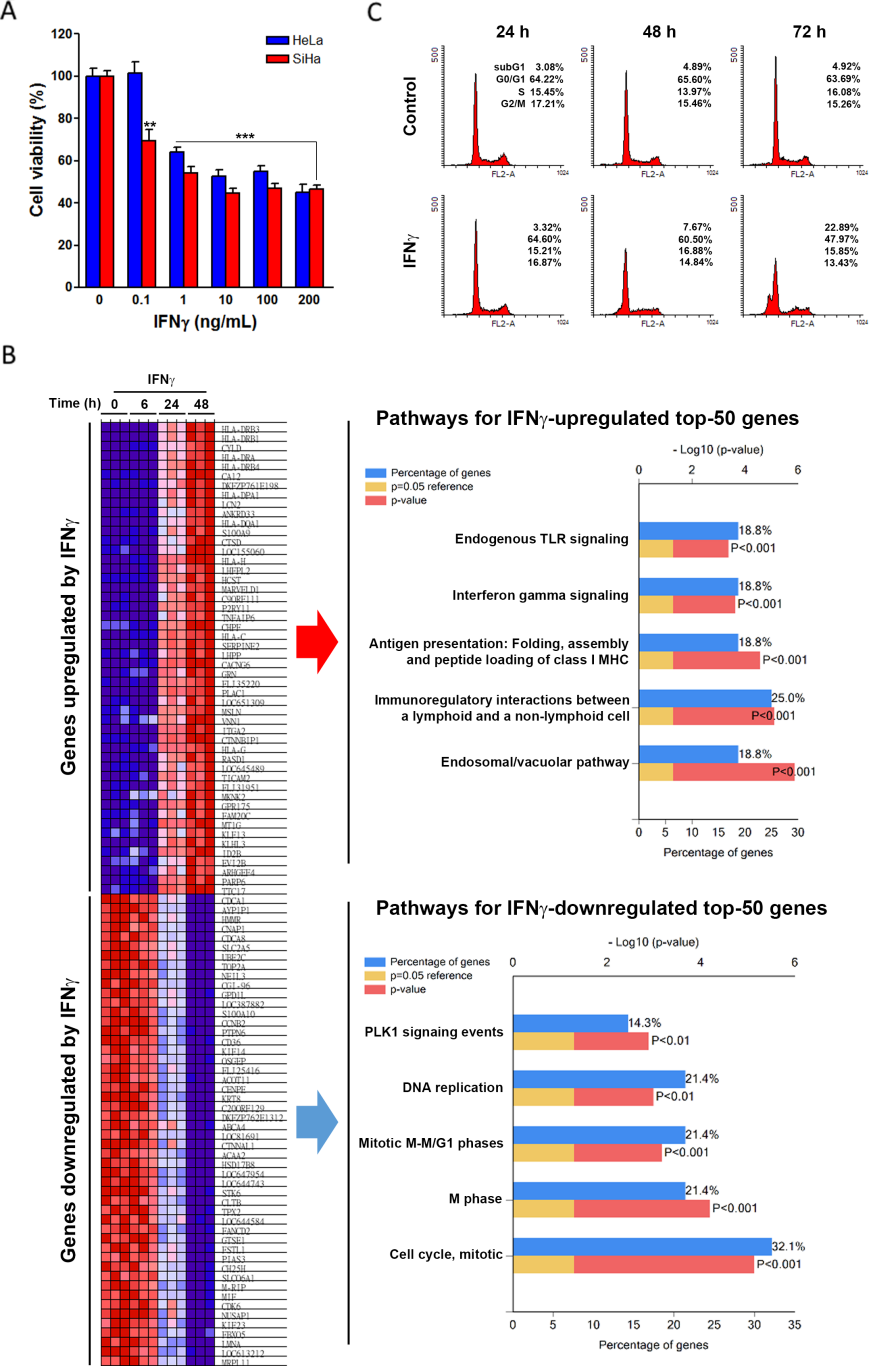
The anticancer effect of IFNγ on cervical cancer **(A)** HeLa and SiHa cells were treated with the indicated doses of IFNγ for 72 h, and then cell viability was examined by MTT assay. A *p* value of < 0.01 (**) or < 0.001(***) indicates significant differences between IFNγ-treated and control cells. **(B)** The microarray data of IFNγ-treated HeLa cells were analyzed by GSEA. The most upregulated and downregulated genes were illustrated on a heat map. Top-50 genes upregulated and downregulated by IFNγ were further analyzed by the FunRich software for pathway enrichment. Pathways were ranked according to the *p* value (red bar). A *p* value lower than 0.05 (yellow bar) was considered significant. The blue bar indicated the percentage of altered genes in a whole pathway. **(C)** HeLa cells were treated with 100 ng/mL IFNγ for 24, 48, and 72 h, and then cell cycle distribution was examined by flow cytometry.

To date, the efficacy of IFNγ and other cytokine-based anticancer therapies remains uncertain, due to their complex effect on both tumor cells and the tumor microenvironment. This study sought to investigate the direct anticancer effect of IFNγ on human cervical cancer; therefore, an *in vitro* IFNγ-treated cell culture model was used. HeLa (adenocarcinoma) and SiHa (grade II squamous cell carcinoma) cell lines, which represent the two major histological types of human cervical cancer, were treated with various doses of IFNγ for 72 h, after which cell viability was examined by MTT assay (Figure 1A). IFNγ at concentrations as low as 0.1∼1 ng/mL exhibited anticancer activity toward both HeLa and SiHa cell lines. Additionally, we found that SiHa cells had higher sensitivity to IFNγ; however, higher concentrations (10∼200 ng/mL) of IFNγ did not reduce the cell viability beyond half. These results suggest that IFNγ is a potential anticancer agent for cervical cancer regardless of the histological subtype.

To identify IFNγ-regulated genes or pathways, a microarray data set (GSE11299 [11]) of HeLa cells treated with 100 ng/mL IFNγ for 6-24 h was obtained from the Gene Expression Omnibus (GEO) database at the National Center for Biotechnology Information (NCBI) [12]. Next, the alterations of genes in response to IFNγ treatment were analyzed and ranked using the Gene Set Enrichment Analysis (GSEA) software [13, 14]. The most upregulated and downregulated genes were illustrated on a heat map (Figure 1B, left part). To determine which biological pathways were altered by IFNγ, the top 50 genes upregulated and top 50 genes downregulated by IFNγ (Supplementary Table 1) were further analyzed using the Functional Enrichment (FunRich) software [15]. We then identified the top 5 upregulated and top 5 downregulated pathways, as shown in Figure 1B (right part). After analysis of these data, it was clear that IFNγ activated pathways were associated with an immune response. Interestingly, IFNγ inhibited pathways seemed to be related to cell cycle regulation and DNA replication, suggesting that IFNγ might cause cell cycle disablement. Indeed, IFNγ has been identified as a mediator of cell cycle regulation in both normal and cancer cells [16–18]. To confirm the effect of IFNγ on the cell cycle, IFNγ-treated HeLa and SiHa cells were analyzed using flow cytometry. As shown in Figure 1C, IFNγ induced apoptosis in HeLa cells as indicated by the increase of subG1 fraction. Additionally, treatment with IFNγ for 24 h was shown to induce G0/G1 arrest in SiHa cells, followed by S/G2/M arrest and apoptosis after 48 and 72 h (Supplementary Figure 1). Cell apoptosis was further confirmed by the Annexin V-FITC/17-AAD double staining. Consistently, HeLa cells had a higher apoptotic portion than SiHa cells (Supplementary Figure 2). Therefore, IFNγ can inhibit the cell viability of cervical SCC and AC cell lines by inducing both cell cycle arrest and apoptosis.

### Identification of cell cycle deregulation as a common hallmark of SCC and AC of the cervix

**Figure 2:**
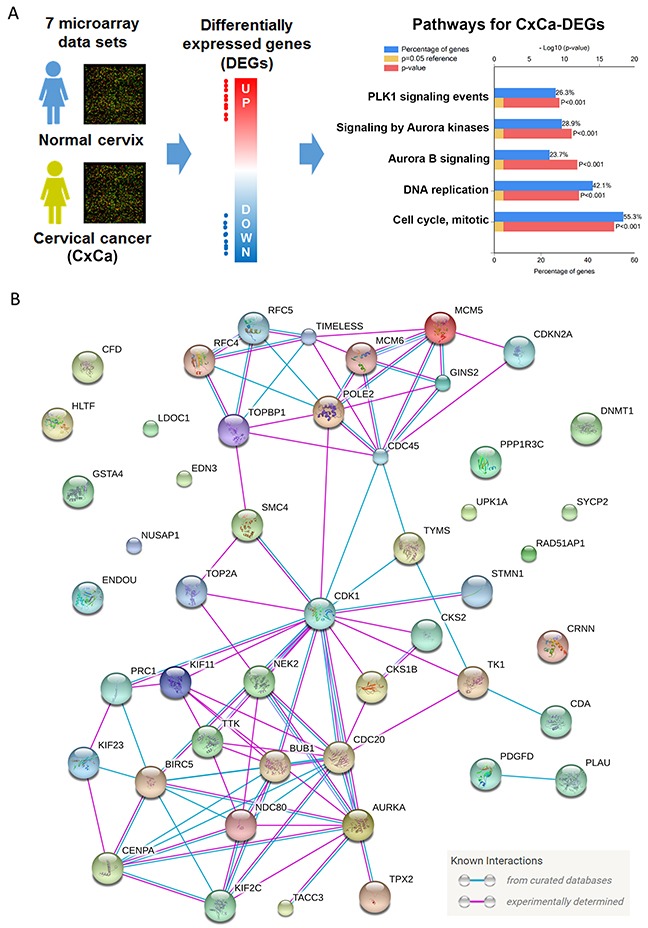
Analysis for the hallmark of cervical cancer **(A)** DEGs obtained from cervical cancerous v.s. normal tissues were analyzed by the FunRich software for pathway enrichment. Pathways were ranked according to the *p* value (red bar). A *p* value less than 0.05 (yellow bar) was considered significant. The blue bar indicated the percentage of altered genes in a whole pathway. **(B)** The network of CxCa-DEGs was reconstituted by the STRING database.

It is well known that cell cycle dysregulation is the major hallmark of many cancer types [19]. Therefore, we hypothesized that inhibition of cell cycle progression-related genes by IFNγ could provide clinical benefits through the reversal of the cancer hallmark. To demonstrate this, a microarray analysis of human cervical cancer tissues was performed (Figure 2A). Seven microarray data sets (Table 1 [20–25]) from healthy donors and patients with cervical cancer were obtained from the NCBI-GEO database. As expected, the majority of these cohorts were SCC cases. Next, the differentially expressed genes (DEGs; listed in Supplementary Table 2) of each data set were prepared by the GEO2R, an R-based web application used to analyze GEO data [12]. We identified 38 upregulated and 10 downregulated genes that were common in cancerous cervical tissues (Table 2). These cervical cancer DEGs (CxCa-DEGs) were then analyzed for biological pathway enrichment using the FunRich software. As shown in Figure 2A, gene alterations related to cell cycle progression (DNA replication and cell cycle, mitotic) and upstream kinases (PLK1 and Aurora kinases) were found. The parallel of biological pathways altered between CxCa-DEGs and IFNγ-downregulated genes (Figures 1B and 2A) may imply the clinical benefit of IFNγ-based therapy through targeting cell cycle deregulation of cervical cancer.

**Table 1:** Summary of 7 cohorts of cervical cancer microarray data sets

GEO accession number	Description	No. of normal tissues	No. of cancer tissues	Reference
GSE7803	Normal squamous cervical epithelia samples and invasive squamous cell carcinomas of the cervix	10	21	[20]
GSE29570	Healthy exocervix and cervical cancer biopsy (cancer type was not specified in this data set)	17	45	[21]
GSE39001 (GPL201)	Healthy endocervix/exocervix and HPV16-positive cervical cancer biopsy (29 were squamous cell carcinomas, 13 were adenocarcinomas, and 1 was adenosquamous carcinoma)	12	43	[23]
GSE39001 (GPL6244)	Healthy exocervix and HPV16-positive cervical cancer biopsy (18 were squamous cell carcinomas and 1 were adenocarcinoma)	5	19	[23]
GSE52903	Healthy exocervix and squamous cell carcinomas of the cervix (51 were squamous cell carcinomas, 3 were adenocarcinomas, and 1 was adenosquamous carcinoma)	17	55	[24]
GSE63514	Normal cervical epithelium and cervical squamous epithelial cancer	24	28	[22]
GSE67522	HPV-negative normal cervical tissues and HPV-positive cervical squamous epithelial cancer tissues	12	20	[25]

The microarray data were downloaded from the NCBI-GEO database. The differential expressed genes (DEGs) from each data set were used to enrich pathways (Figure 2) and to query the CMap (Figure 7).

**Table 2:** The gene list for the differentially expressed genes of cervical cancer (CxCa-DEGs) and the cervical cancer signature (CxCa-Sig)

	CxCa-DEGs	CxCa-Sig
Upregulated genes	AURKA, BIRC5, BUB1, CDC20, CDC45, CDK1, CDKN2A, CENPA, CKS1B, CKS2, DNMT1, GINS2, HLTF, KIF11, KIF23, KIF2C, MCM5, MCM6, NDC80, NEK2, NUSAP1, PLAU, POLE2, PRC1, RAD51AP1, RFC4, RFC5, SMC4, STMN1, SYCP2, TACC3, TIMELESS, TK1, TOP2A, TOPBP1, TPX2, TTK, TYMS (38 genes)	AURKA, BIRC5, BUB1, CDC20, CDC45, CDK1, CDKN2A, CENPA, CKS1B, CKS2, GINS2, KIF11, KIF23, KIF2C, MCM5, MCM6, NDC80, NEK2, POLE2, PRC1, RFC4, RFC5, SMC4, STMN1, TACC3, TIMELESS, TK1, TOP2A, TOPBP1, TPX2, TTK, TYMS (32 genes)
Downregulated genes	CDA, CFD, CRNN, EDN3, ENDOU, GSTA4, LDOC1, PDGFD, PPP1R3C, UPK1A (10 genes)	CDA (1 gene)

CxCa-DEGs represented the overlapped DEGs from 7 microarray data sets. The network of CxCa-DEGs was reconstructed by the STRING database. The genes directly linked to each other were used as a CxCa-Sig for further analyses.

**Figure 3:**
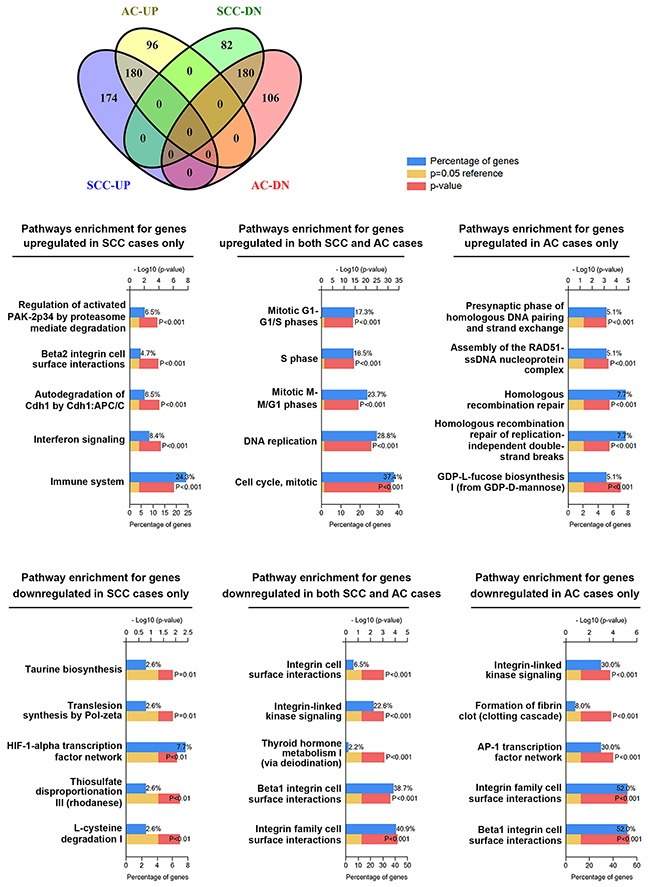
Pathways enrichment in cervical SCC and AC subtypes The DEGs for SCC and AC were prepared from the data set GSE39001 (GPL201). The common and specific DEGs were virtualized with a Venn diagram. Then, biological pathway enrichment for each category was performed using the FunRich software.

To further clarify whether cell cycle deregulation is a common hallmark between SCC and AC, a data set GSE39001 (GPL201) containing 29 SCC and 13 AC cases [23] was used to prepare DEGs (Supplementary Table 3). The DEGs of SCC and AC cases were further divided into SCC-specific DEGs, AC-specific DEGs, and common DEGs in SCC and AC cases (Supplementary Table 4). These sets of DEGs were shown using a Venn diagram (Figure 3) that was generated using an online Venny tool [26]. Next, biological pathway enrichment using the FunRich software was performed to compare the similarities and differences between SCC and AC. Only pathways with 10% or more genes were considered. As shown in Figure 3, genes related to cell cycle progression and integrin signaling were respectively upregulated and downregulated in both SCC and AC subtypes; however, we also found differences between SCC and AC cases. Genes related to the immune system were upregulated in SCC cases only, and a larger number of genes related to integrin signaling were downregulated in AC cases. In addition, genes related to AP-1 transcription factor network were also downregulated in AC cases. This analysis shows that cell cycle deregulation is indeed a common hallmark in cervical cancer regardless of the histological subtypes.

### Establishment of a cervical cancer gene signature that is associated with poor clinical outcomes

**Figure 4:**
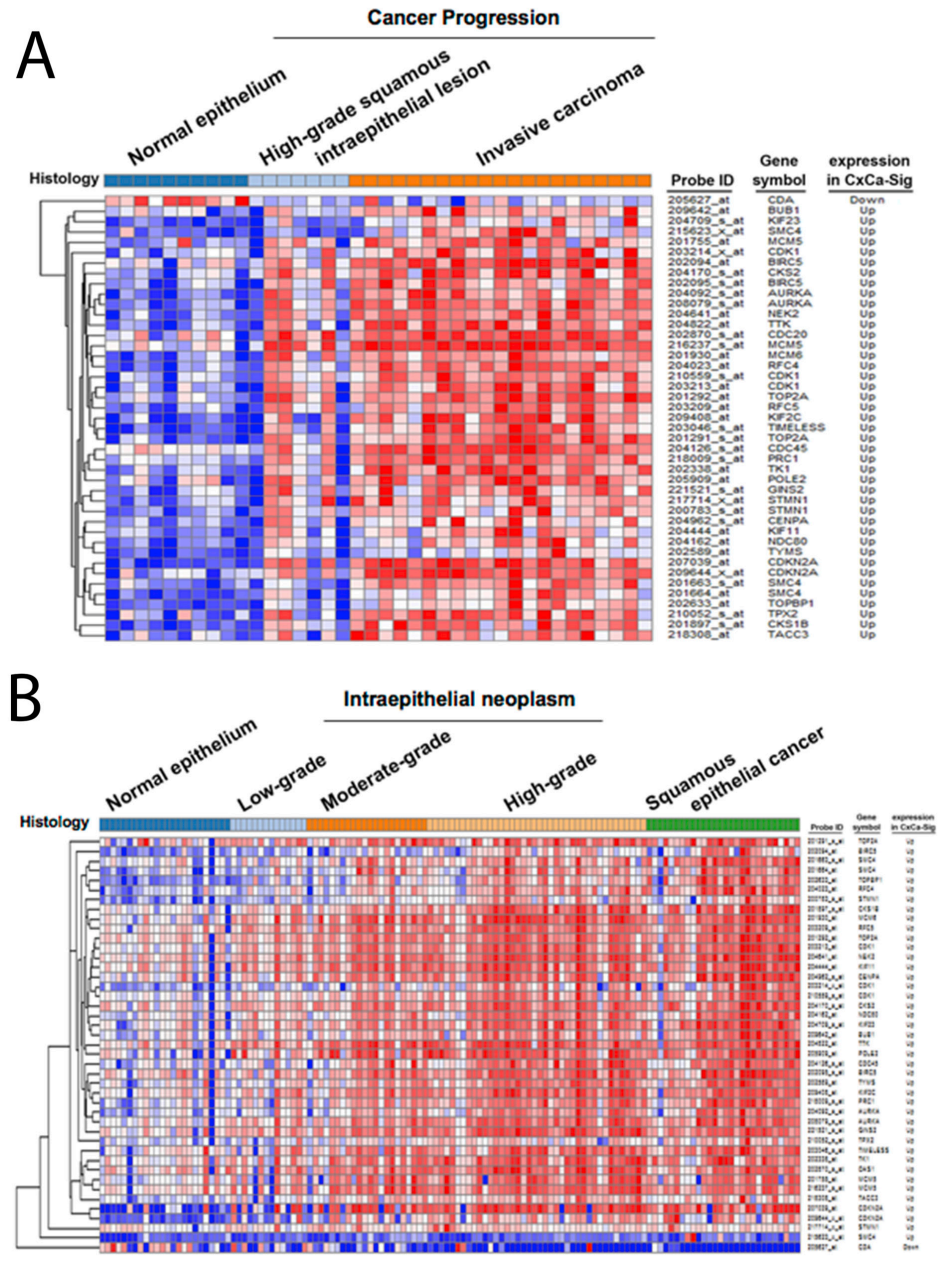
The expression of CxCa-Sig during cervical cancer progression Clustering analysis was performed to examine the expression of CxCa-Sig during cancer progression from normal epithelium, intraepithelial neoplasm, to cervical cancer in two data sets, GSE7803 **(A)** and GSE63514 **(B)**.

**Figure 5:**
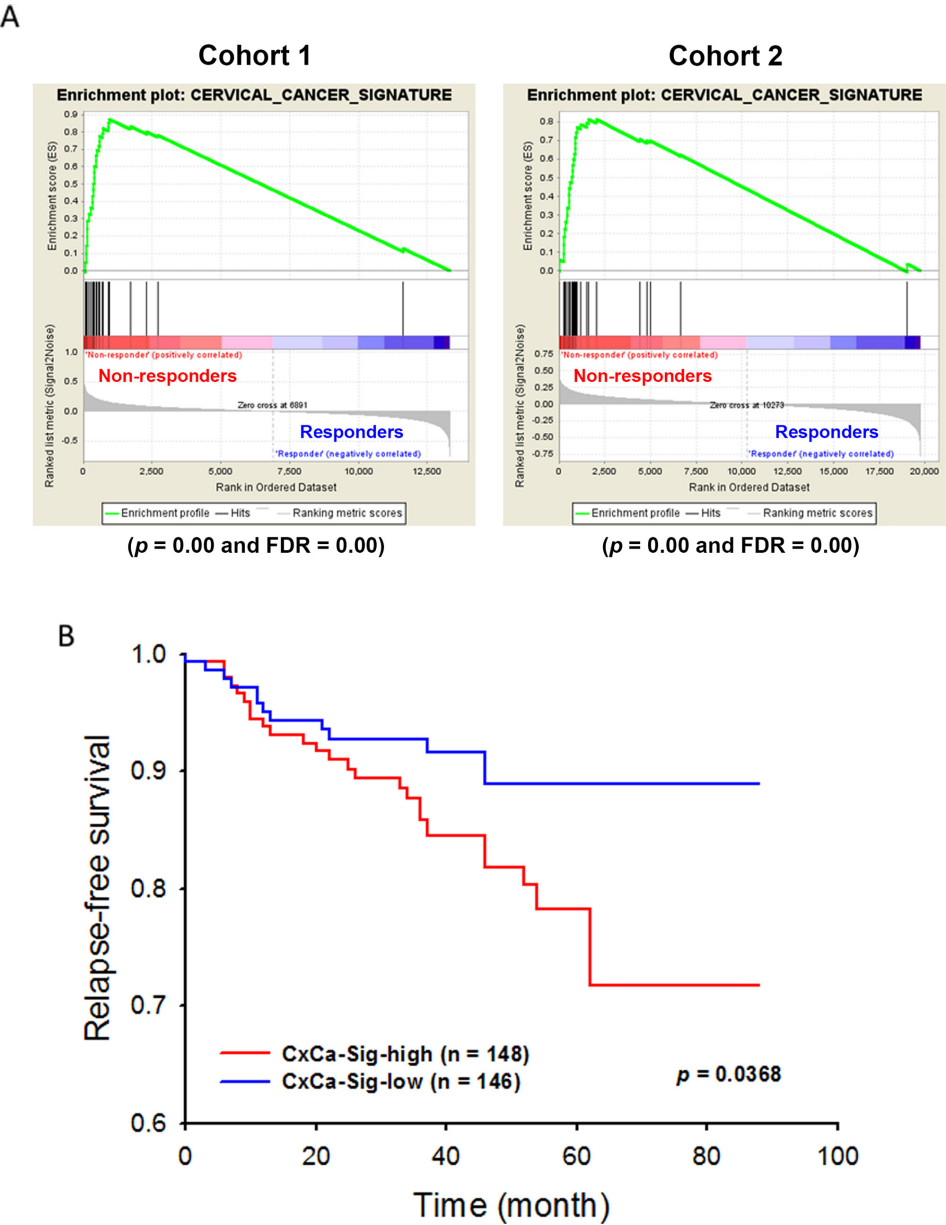
The role of CxCa-Sig in clinical outcomes of cervical cancer patient The expression of CxCa-Sig in cervical cancer patients containing responders and non-responders to neoadjuvant chemotherapy using nedaplatin and irinotecan before the surgery **(A)**, and chemoradiotherapy using cisplatin with or without surgery **(B)** was analyzed by GSEA.

To establish a gene signature of cervical cancer for further analyses, the network of CxCa-DEGs was reconstructed by the Search Tool for the Retrieval of Interacting Genes/Proteins (STRING) database that can provide the assessment and integration of protein-protein interactions, including direct (physical) and indirect (functional) associations [27]. As shown in Figure 2B, the major network consisted of 32 upregulated genes and one downregulated gene (listed in Table 2), and was designated as a cervical cancer signature (CxCa-Sig). Clustering analysis and heat map visualization showed that CxCa-Sig was consecutively altered during the cervical cancer progression from normal epithelium, to intraepithelial lesion, to invasive carcinoma in two data sets (Figure 4A: GSE7803 [20]; Figure 4B: GSE63514 [22]) of cervical SCC patient cohorts. In addition, GSEA was performed to test whether the expression of CxCa-Sig was associated with clinical outcome. Two cohorts of cervical SCC patients were used, which contained responders and non-responders to neoadjuvant chemotherapy using nedaplatin and irinotecan before surgery (cohort 1, GSE70035 [28]), and chemoradiotherapy using cisplatin with or without surgery (cohort 2, GSE56363 [29]). As shown in the enrichment plot (Figure 5A) and the heat map (Supplementary Figure 3), CxCa-Sig was enriched in non-responders in both cohorts (*p* value = 0.00 and FDR = 0.00), indicating that cervical cancer patients with lower expression of CxCa-Sig respond better to therapy. Furthermore, Kaplan-Meier (KM) survival analysis using the PROGgeneV2 prognostic database [30] demonstrated that higher expression of CxCa-Sig was associated with shorter relapsed-free survival (Figure 5B). These results suggest that CxCa-Sig was associated with poor clinical outcomes.

### IFNγ significantly reverses the cervical cancer signature

**Figure 6:**
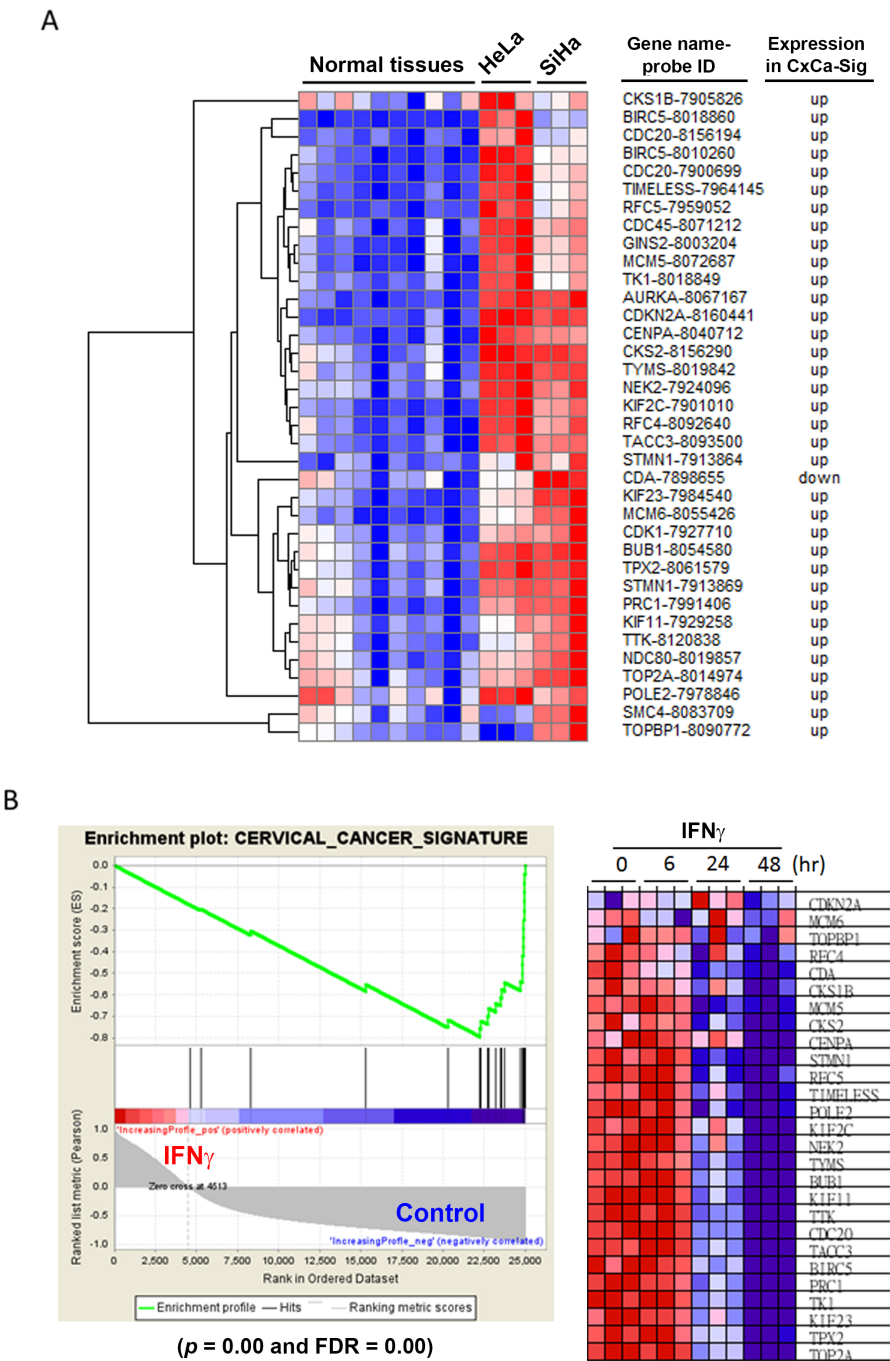
Effects of IFNγ on the expression of CxCa-Sig in HeLa cells **(A)** Clustering analysis was performed to examine the expression of CxCa-Sig in HeLa and SiHa cells compared to normal cervix. **(B)** The microarray data of IFNγ-treated HeLa cells were analyzed by GSEA for the expression of CxCa-Sig.

Because IFNγ downregulated cell cycle-related gene expression (Figure 1B), resembling the CxCa-DEGs (Figure 2A), we hypothesized that the expression of CxCa-Sig would be suppressed by IFNγ treatment. Higher expression of CxCa-Sig in HeLa and SiHa cells compared to normal tissues (microarray data set: GSE29216 [31]) was confirmed by clustering analysis (Figure 6A). To test whether the anticancer activity of IFNγ *in vitro* (Figure 1A) was indeed associated with the reversion of CxCa-Sig, a GSEA of microarray data in IFNγ-treated HeLa cells (GSE11299 [11]) was performed. As shown in Figure 6B (left part), CxCa-Sig was enriched in untreated control cells with statistical significance (*p* value = 0.00; FDR = 0.00). Heat map visualization showed that IFNγ could reverse the CxCa-Sig after 24 h treatment (Figure 6B, right part). Thus, these results indicate that IFNγ is able to reverse the gene signature of cervical cancer.

### Identification of apgenin as a new drug combination with IFNγ for treating cervical AC cells

**Figure 7:**
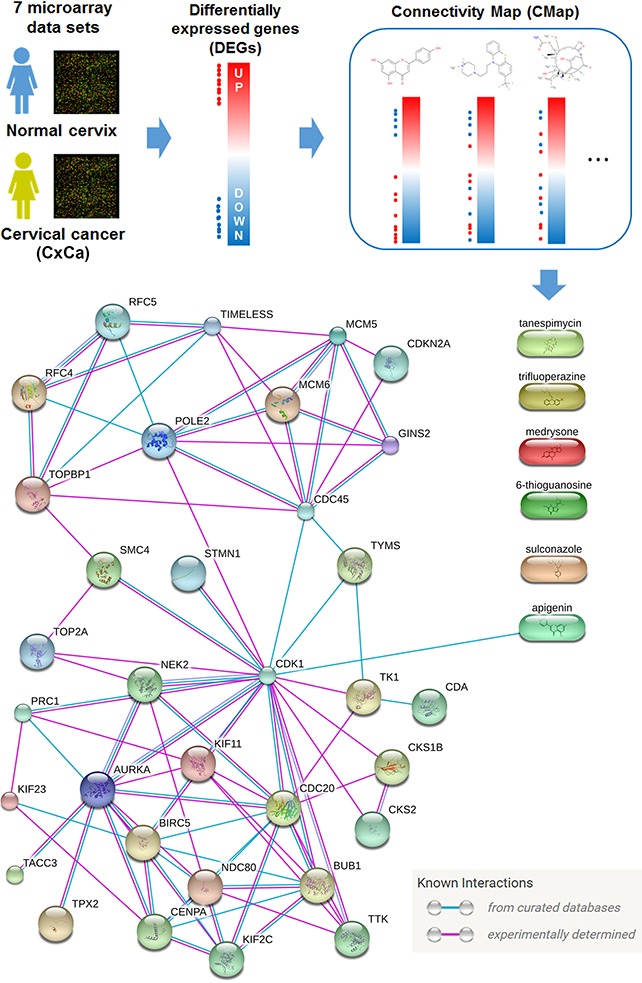
Identification of apigenin as an anticancer agent for cervical cancer Upper part: DEGs obtained from cervical cancerous v.s. normal tissues were queried using the CMap database for potential anticancer agents against cervical cancer. Lower part: Six candidate compounds were further analyzed by the STITCH database for their connectivity to CxCa-Sig.

Because the anticancer activity of IFNγ was limited (Figure 1A), we attempted to search for original drug combinations with IFNγ using the Connectivity Map (CMap) software (Figure 7). CMap is a chemical genomics database that collects gene-expression profiles from cultured human cells treated with small molecules. It can be used to find small molecules that reverse a specific gene expression profile [32]. DEGs (listed in Supplementary Table 2) of microarray data sets with normal and cancerous tissues (Table 1) were queried using the CMap. From the queried results of the seven microarray data sets there are six common drugs (Table 3). CMap drugs with negative mean scores reversed (or opposed) gene expression profiles with DEGs (Figure 7, upper part). The relationship between CxCa-Sig and CMap drugs were further established using the Search Tool for Interactions of Chemicals (STITCH) database, which can explore known and predicted interactions of chemicals and proteins through evidence derived from experiments, databases, and literature [33]. As shown in Figure 7 (lower part), only apigenin was linked to the network of CxCa-Sig through targeting CDK1. In addition, the average mean score of apigenin (−0.771) indicated that it was the most potent drug, able to reverse the expression of CxCa-DEGs (Table 3). To investigate whether apigenin is a common drug that can reverse the gene expression profiles of both SCC and AC, the DEGs of SCC and AC (Supplementary Table 3) were queried using the CMap. As shown in Supplementary Table 4 and Supplementary Figure 4, five, including apigenin, of the six common CMap drugs (Table 3) were also predicted in SCC and AC data sets. Therefore, apigenin was chosen for combination with IFNγ.

**Table 3:** Summary of predicted CMap drugs

Drug name	Pharmacologic action	Average mean score
Apigenin	A natural product belonging to the flavone class that is the aglycone of several naturally occurring glycosides	−0.771
Thioguanosine	A purine analog showing antineoplastic activity	−0.757
Sulconazole	An antifungal medication of the imidazole class	−0.706
Medrysone	A corticosteroid that has been used in optometry, and in ophthalmology for the treatment of eye inflammations	−0.684
Trifluoperazine	A typical antipsychotic of the phenothiazine chemical class	−0.5
Tanespimycin	Heat shock protein 90 (HSP90) inhibitor	−0.418

Each profile of differential expressed genes (DEGs) from 7 cohorts of cervical cancer microarray data sets were used to query the CMap. Only the results of CMap drugs with enrichment score < 0 and *p* value < 0.01 were considered significant. The predicted CMap drugs overlapped in these data sets were listed in this table and ranked according their average mean scores. The more negative in average mean score means the higher possibility of CMap drugs to reverse the DEGs.

**Figure 8:**
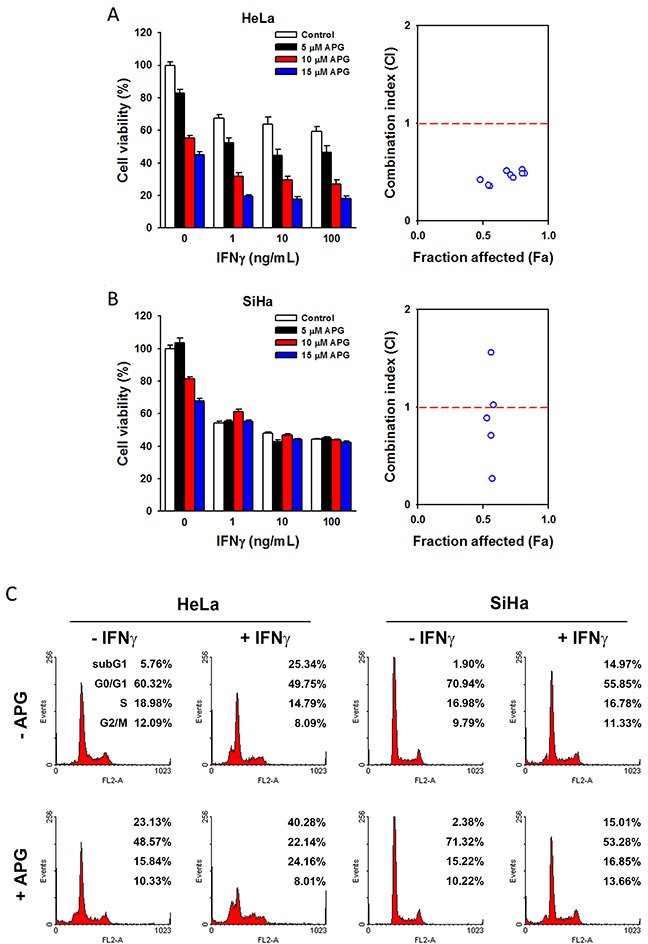
The combinational anticancer activity of apigenin and IFNγ **(A, B)** HeLa and SiHa cells were treated with different doses of apigenin and IFNγ (alone or in combinations) for 72 h. In left parts, the cell viability was analyzed by an MTT assay. In right parts, the combination index (CI) was calculated as described in the Materials and Methods, and then plotted against the values of fraction affected (Fa). The CI values higher than 2 were not shown in Fa-CI plot. **(C)** HeLa and SiHa cells were treated with 100 ng/mL IFNγ for 72 h with or without 10 μM apigenin, and then cell cycle distribution was examined by flow cytometry.

Apigenin's (APG) cytotoxicity in HeLa and SiHa cells was analyzed by MTT assay using the physiologically relevant doses (5∼15 μM) [34–38]. We found that HeLa cells were more sensitive than SiHa cells in response to apigenin treatment (Figure 8A and 8B, left parts), suggesting that the anticancer activity of apigenin may depend on the histological type of cervical cancer. Consistently, combination with IFNγ treatment further reduced cell viability in HeLa cells, but not in SiHa cells (Figure 8A and 8B, left parts). To analyze the combination effect of APG and IFNγ, combination index (CI) was calculated by the CompuSyn software according to the Chou-Talalay method [39]. As shown in Figure 8A (right part), the CI values of each APG/IFNγ combination in HeLa cells were lower than 1, indicating that APG exhibits a synergistic effect in combination with IFNγ. However, the synergistic interaction between APG and IFNγ was not observed in SiHa cells (Figure 8B, right part). Furthermore, apigenin enhanced the apoptosis-inducing effects of IFNγ in HeLa cells, but not in SiHa cells (Figure 8C). In conclusion, these results suggest that apigenin enhances the anticancer activity of IFNγ in HeLa cervical AC cells.

## DISCUSSION

The clinical management of cervical cancer remains a challenge for researchers and health professionals, and the development of inventive anticancer strategies is crucial. Two major histologic subtypes of human cervical cancer are squamous cell carcinoma (SCC) and adenocarcinoma (AC). Current regimens do not discriminate between SCC and AC patients; however, their clinical responses are very different. Generally, AC patients have a worse prognosis than patients with SCC; therefore, more specific therapeutic strategy for different histological subtypes of cervical cancer are needed. This study provides a molecular basis for the application of IFNγ-based therapy alone, or in combination with apigenin, a dietary flavonoid, for the selective treatment of cervical SCC and AC. Bioinformatics analyses suggest that cell cycle dysregulation was a general hallmark of SCC and AC of the cervix, which can be reversed by IFNγ. *In vitro* experimental analyses demonstrated IFNγ's anticancer activity against both cervical SCC and AC cells. In addition, apigenin was found to be an effective combination strategy to treat cervical AC subtypes. Thus, our study provides a therapeutic option for treating the different histological subtypes of cervical cancer in the future.

In a previous study of gene network reconstruction using DEGs in cervical cancer [40], cell cycle gene networking was identified as a major driving force of cervical cancer. Through direct stimulation, cell cycle genes synergize with E6/E7 oncoprotein-mediated blockade of cell cycle suppressors, p53 and RB, to promote cancer progression [40]. More recently, an *in silico* pathway analysis revealed that deregulation of the cell cycle is one of the major trademarks in cervical cancer [41]. In addition, this study also suggests that the major components of cell cycle pathway, such as CDK1/2, could be potential new targets for treating cervical cancer [41]. However, this concept has not yet been demonstrated. Our study not only showed that cell cycle deregulation is an indicator of cervical cancer, it also confirmed that targeting the cell cycle by IFNγ and apigenin kills cervical cancer cell lines by triggering cell cycle arrest and apoptosis.

Because SCC is the predominant histological subtype of cervical cancer, bioinformatics analyses described above focus on SCC cases [40, 41]. Currently, there is an urgent need for analyses to discriminate between SCC and AC cases. Two previous studies have been conducted to compare the gene expression profiling of cervical SCC and AC. They focus on the specific genes upregulated in either SCC or AC cases [42, 43]. However, our bioinformatics analyses focused on the biological pathways, not individual genes, to discriminate both similarities and differences between SCC and AC cancer. Our results may explain how the different histological origins of HeLa and SiHa cells contribute to their differential sensitivity to IFNγ and apigenin. The anticancer activity of IFNγ was associated with the activation of an immune response and the inhibition of cell cycle regulation and DNA replication. Resultantly, the enrichment of genes related to immune system may be associated with the higher sensitivity of the SCC (SiHa) cell line to IFNγ. In addition, the AC (HeLa) cell line was significantly more sensitive to apigenin alone or in combination with IFNγ. This may be due to the downregulation of AP-1 transcription factor network because apigenin is able to block phorbol ester (PMA)-mediated cell survival through suppressing AP-1 activity [44].

Apigenin is abundant in fruits and vegetables. It has been shown to exhibit anti-inflammatory, antioxidant, and anticancer properties with high selectivity to cancer cells and a high safety threshold [45]. A review article comprehensively analyzes the cytotoxicity of several flavonoids against different types of human cancer cells, which are compiled from assorted literature sources. After statistical calculation of the respective mean parameters, it was postulated that apigenin is probably more potent and sensitive in killing cervical cancer cells than other cancer cell types [46]. Therefore, combination therapy with apigenin and IFNγ is a promising therapeutic strategy for cervical cancer.

Our previous study has shown that the combination of apigenin treatment with therapeutic HPV DNA vaccination generates an enhanced *in vitro* and *in vivo* anticancer effect against cervical cancer [47]. Apigenin enhances the ability of therapeutic HPV DNA vaccination to activate IFNγ-producing CD8+ cells [47], implying that vaccination-induced IFNγ and apigenin act together to kill tumor cells. Indeed, the direct anticancer effect of IFNγ alone and in combination with apigenin was demonstrated in this study. Moreover, apigenin is shown to induce apoptosis of human hepatocellular carcinoma HepG2 cells through the releases of IFNγ [48]. Taken together, these studies indicate that apigenin may be able to trigger the production of IFNγ through tumor and immune cells. Consequently, the increase of IFNγ further potentiates the anticancer activity of apigenin.

One potential problem for using apigenin as an anticancer agent is its low bioavailability [49]. However, the slow absorption and elimination property of apigenin may allow it to accumulate within tissues [50]. In addition, biodistribution analysis for the uptake of ^131^I-labeled apigenin in female rats indicates that apigenin has the highest uptake in the intestine and uterus [51], which may lead to the accessibility and accumulation of apigenin to cervical cancer tumor tissues, as the cervix is the lower part of the uterus.

The bioinformatics analyses suggest that apigenin targets to CDK1-related pathways. Previous studies indicate that higher concentrations (40∼100 μM) of apigenin are required for its inhibition on CDK1's expression and activity [52–55]. In this study, we found that a lower dose (15 μM) of apigenin was sufficient to reduce the expression of CDK1 in HeLa cells, but not in SiHa cells (Supplementary Figure 5), which may also explain the differential sensitivity of these two cell lines in response to apigenin. We proposed that higher concentrations of apigenin or more specific CDK1 inhibitors may exhibit higher potency to kill SiHa cells. However, current results still cannot exclude the possibility that other mechanisms are involved because multiple cellular and molecular targets of apigenin have been identified [45]. Further studies are needed to warrant the targets of apigenin.

There were several limitations in this study. First, only *in silico* and *in vitro* experimental analyses were performed. Further investigations using animal models specific for cervical SCC or AC subtypes were required. Second, only 13 cases of AC patients were available for bioinformatics analyses. Third, gene expression signature-based approaches were used in this study. The differences of genetic mutations, epigenetics, proteomics, and metabolomics between SCC and AC subtypes should also be considered in future investigations.

In conclusion, this study employs bioinformatics and *in vitro* experimental approaches to investigate the anticancer effect of IFNγ against human cervical SCC and AC. In addition, apigenin is identified as a repurposed drug when combined with IFNγ to treat AC of the cervix. The anticancer activities of apigenin and IFNγ are associated with the inhibition of cell cycle progression and induction of apoptosis. Our study provides an innovative strategy for treating cervical cancer derived from different histological origins.

## MATERIALS AND METHODS

### Gene set enrichment analysis (GSEA)

GSEA v2.2.2 software (http://www.broadinstitute.org/gsea/) was used to analyze microarray data [13, 14]. Genes were ranked by running a gene set type permutation test with log2 ratio ranking statistic. Default settings were used for all other GSEA parameters. Gene sets with a *p* value < 0.01 and a false discovery rate (FDR) < 0.05 were considered significant.

### Pathway enrichment analysis and pathway reconstruction

Pathway analysis was performed using the FunRich v.2.1.2 software (http://www.funrich.org) [15]. For the pathway reconstruction of genes, the STRING v10.0 database was used (http://string-db.org/) [27]. The parameters were set as follows: organism = homo sapiens; active prediction methods = experiments and databases; minimum required interaction score = medium confidence (0.400); max number of interactors to show = none (query proteins only); and view setting = evidence view.

### Chemical-gene connectivity by the Search Tool for Interactions of Chemicals (STITCH) database

The relationship between chemicals and genes was analyzed using the STITCH v5.0 (http://stitch-beta.embl.de/) [33]. The parameters were set as follows: organism = homo sapiens; active prediction methods = experiments, databases and predictions; minimum required interaction score = medium confidence (0.400); max number of interactors to show = none (query proteins only); and view setting = evidence view.

### Chemicals and reagents

RPMI-1640 medium, L-glutamine, sodium pyruvate, penicillin, and streptomycin were purchased from Life Technologies. Fetal bovine serum (FBS) was purchased from Hyclone. Apigenin, 3-(4,5-Dimethylthiazol- 2-yl)-2,5-diphenyl tetrazolium bromide (MTT), dimethyl sulfoxide (DMSO), propidium iodide (PI), and ribonuclease A (RNase A) were purchased from Sigma. Recombinant human IFNγ was purchased from PeproTech. Antibodies specific for phospho-CDK1 (Tyr15) and total CDK1 were purchased from Cell Signaling.

### Cell culture

HeLa (human cervix adenocarcinoma) and SiHa (human cervix grade II squamous cell carcinoma) cell lines were obtained from American Type Culture Collection (ATCC). Cells were cultured in RPMI-1640 medium supplemented with 10% FBS, 1 mM sodium pyruvate, 2 mM L-glutamine, 100 IU/mL pencillin, and 100 μg/mL streptomycin. Cells were incubated at 37°C in a humidified incubator containing 5% CO_2_.

### Cell viability assay

Cell viability was measured with a MTT assay. Cells were plated in 96-well plates and treated with drugs. After 72 h of incubation, 0.5 mg/mL of MTT was added to each well for an additional 4 h. The blue MTT formazan precipitate was then dissolved in 100∼200 μL of DMSO. The absorbance at 570 nm was measured on a multiwell plate reader. Cell viability was expressed as the percentage of surviving cells related to untreated control cells. The fraction affected (Fa) representing the respective growth inhibition was determined (for example, The Fa value of 70% growth inhibition is 0.7). The combination index (CI) was calculated by the CompuSyn software (ComboSyn, Inc.) according to the Chou-Talalay method [39]. The CI value quantitatively defines synergistic cytotoxicity (< 1), addictive cytotoxicity (= 1), and antagonistic cytotoxicity (> 1).

### Flow cytometry analysis

Cells were plated in 60-cm dishes for 24 h, and then treated with complete medium containing drugs for 24, 48, and 72 h. Floating and adherent cells were harvested and immediately fixed with 75% ethanol and stored at -20°C. Cells were stained in staining buffer (10 μg/mL PI and 100 μg/mL RNase A) for 30 min and then analyzed on a flow cytometry (FACSCalibur, BD Biosciences). Data were analyzed by WinMDI 2.9 free software (Scripps Research Institute).

### Statistical analysis

Means and standard deviations of samples in MTT cell viability assays were calculated from the numerical data generated in this study. Data were analyzed using Student's *t*-test, and *p* values of < 0.05 were considered significant (*). Hierarchical clustering analysis by one minus Pearson's correlation metric was performed using the GENE-E software (http://www.broadinstitute.org/cancer/software/GENE-E/). The prognostic impact of the cervical cancer gene signature (CxCa-Sig) was analyzed using the PROGgeneV2 database (http://www.compbio.iupui.edu/proggene/) [30]. Patient survival data from the GSE44001 [56] data set was used to construct Kaplan-Meier (KM) survival plot.

## SUPPLEMENTARY FIGURES AND TABLES











## References

[1] zur Hausen H (2002). Papillomaviruses and cancer: from basic studies to clinical application. Nat Rev Cancer.

[2] Munger K, Scheffner M, Huibregtse JM, Howley PM (1992). Interactions of HPV E6 and E7 oncoproteins with tumour suppressor gene products. Cancer Surv.

[3] Yee GP, de Souza P, Khachigian LM (2013). Current and potential treatments for cervical cancer. Curr Cancer Drug Targets.

[4] Gien LT, Beauchemin MC, Thomas G (2010). Adenocarcinoma: a unique cervical cancer. Gynecol Oncol.

[5] Chen RJ, Lin YH, Chen CA, Huang SC, Chow SN, Hsieh CY (1999). Influence of histologic type and age on survival rates for invasive cervical carcinoma in Taiwan. Gynecol Oncol.

[6] Eifel PJ, Burke TW, Morris M, Smith TL (1995). Adenocarcinoma as an independent risk factor for disease recurrence in patients with stage IB cervical carcinoma. Gynecol Oncol.

[7] Hopkins MP, Morley GW (1991). A comparison of adenocarcinoma and squamous cell carcinoma of the cervix. Obstet Gynecol.

[8] Lai CH, Hsueh S, Hong JH, Chang TC, Tseng CJ, Chou HH, Huang KG, Lin JD (1999). Are adenocarcinomas and adenosquamous carcinomas different from squamous carcinomas in stage IB and II cervical cancer patients undergoing primary radical surgery?. Int J Gynecol Cancer.

[9] Miller CH, Maher SG, Young HA (2009). Clinical use of interferon-gamma. Ann N Y Acad Sci.

[10] Zaidi MR, Merlino G (2011). The two faces of interferon-gamma in cancer. Clin Cancer Res.

[11] Ni Z, Abou El Hassan M, Xu Z, Yu T, Bremner R (2008). The chromatin-remodeling enzyme BRG1 coordinates CIITA induction through many interdependent distal enhancers. Nat Immunol.

[12] Barrett T, Wilhite SE, Ledoux P, Evangelista C, Kim IF, Tomashevsky M, Marshall KA, Phillippy KH, Sherman PM, Holko M, Yefanov A, Lee H, Zhang N (2013). NCBI GEO: archive for functional genomics data sets--update. Nucleic Acids Res.

[13] Mootha VK, Lindgren CM, Eriksson KF, Subramanian A, Sihag S, Lehar J, Puigserver P, Carlsson E, Ridderstrale M, Laurila E, Houstis N, Daly MJ, Patterson N (2003). PGC-1alpha-responsive genes involved in oxidative phosphorylation are coordinately downregulated in human diabetes. Nat Genet.

[14] Subramanian A, Tamayo P, Mootha VK, Mukherjee S, Ebert BL, Gillette MA, Paulovich A, Pomeroy SL, Golub TR, Lander ES, Mesirov JP (2005). Gene set enrichment analysis: a knowledge-based approach for interpreting genome-wide expression profiles. Proc Natl Acad Sci U S A.

[15] Pathan M, Keerthikumar S, Ang CS, Gangoda L, Quek CY, Williamson NA, Mouradov D, Sieber OM, Simpson RJ, Salim A, Bacic A, Hill AF, Stroud DA (2015). FunRich: an open access standalone functional enrichment and interaction network analysis tool. Proteomics.

[16] Kano A, Watanabe Y, Takeda N, Aizawa S, Akaike T (1997). Analysis of IFN-gamma-induced cell cycle arrest and cell death in hepatocytes. J Biochem.

[17] Burke F, East N, Upton C, Patel K, Balkwill FR (1997). Interferon gamma induces cell cycle arrest and apoptosis in a model of ovarian cancer: enhancement of effect by batimastat. Eur J Cancer.

[18] Wall L, Burke F, Barton C, Smyth J, Balkwill F (2003). IFN-gamma induces apoptosis in ovarian cancer cells in vivo and in vitro. Clin Cancer Res.

[19] Hanahan D, Weinberg RA (2011). Hallmarks of cancer: the next generation. Cell.

[20] Zhai Y, Kuick R, Nan B, Ota I, Weiss SJ, Trimble CL, Fearon ER, Cho KR (2007). Gene expression analysis of preinvasive and invasive cervical squamous cell carcinomas identifies HOXC10 as a key mediator of invasion. Cancer Res.

[21] Guardado-Estrada M, Medina-Martinez I, Juarez-Torres E, Roman-Bassaure E, Macias L, Alfaro A, Alcantara-Vazquez A, Alonso P, Gomez G, Cruz-Talonia F, Serna L, Munoz-Cortez S, Borges-Ibanez M (2012). The Amerindian mtDNA haplogroup B2 enhances the risk of HPV for cervical cancer: de-regulation of mitochondrial genes may be involved. J Hum Genet.

[22] den Boon JA, Pyeon D, Wang SS, Horswill M, Schiffman M, Sherman M, Zuna RE, Wang Z, Hewitt SM, Pearson R, Schott M, Chung L, He Q (2015). Molecular transitions from papillomavirus infection to cervical precancer and cancer: role of stromal estrogen receptor signaling. Proc Natl Acad Sci U S A.

[23] Espinosa AM, Alfaro A, Roman-Basaure E, Guardado-Estrada M, Palma I, Serralde C, Medina I, Juarez E, Bermudez M, Marquez E, Borges-Ibanez M, Munoz-Cortez S, Alcantara-Vazquez A (2013). Mitosis is a source of potential markers for screening and survival and therapeutic targets in cervical cancer. PLoS One.

[24] Medina-Martinez I, Barron V, Roman-Bassaure E, Juarez-Torres E, Guardado-Estrada M, Espinosa AM, Bermudez M, Fernandez F, Venegas-Vega C, Orozco L, Zenteno E, Kofman S, Berumen J (2014). Impact of gene dosage on gene expression, biological processes and survival in cervical cancer: a genome-wide follow-up study. PLoS One.

[25] Sharma S, Mandal P, Sadhukhan T, Roy Chowdhury R, Ranjan Mondal N, Chakravarty B, Chatterjee T, Roy S, Sengupta S (2015). Bridging links between long noncoding RNA HOTAIR and HPV Oncoprotein E7 in cervical cancer pathogenesis. Sci Rep.

[26] Oliveros JC (2007-2015). Venny. An interactive tool for comparing lists with Venn's diagrams. http://bioinfogp.cnb.csic.es/tools/venny/index.html.

[27] Szklarczyk D, Franceschini A, Wyder S, Forslund K, Heller D, Huerta-Cepas J, Simonovic M, Roth A, Santos A, Tsafou KP, Kuhn M, Bork P, Jensen LJ (2015). STRING v10: protein-protein interaction networks, integrated over the tree of life. Nucleic Acids Res.

[28] Horikawa N, Baba T, Matsumura N, Murakami R, Abiko K, Hamanishi J, Yamaguchi K, Koshiyama M, Yoshioka Y, Konishi I (2015). Genomic profile predicts the efficacy of neoadjuvant chemotherapy for cervical cancer patients. BMC Cancer.

[29] Balacescu O, Balacescu L, Tudoran O, Todor N, Rus M, Buiga R, Susman S, Fetica B, Pop L, Maja L, Visan S, Ordeanu C, Berindan-Neagoe I (2014). Gene expression profiling reveals activation of the FA/BRCA pathway in advanced squamous cervical cancer with intrinsic resistance and therapy failure. BMC Cancer.

[30] Goswami CP, Nakshatri H (2014). PROGgeneV2: enhancements on the existing database. BMC Cancer.

[31] Vazquez-Mena O, Medina-Martinez I, Juarez-Torres E, Barron V, Espinosa A, Villegas-Sepulveda N, Gomez-Laguna L, Nieto-Martinez K, Orozco L, Roman-Basaure E, Munoz Cortez S, Borges Ibanez M, Venegas-Vega C (2012). Amplified genes may be overexpressed, unchanged, or downregulated in cervical cancer cell lines. PLoS One.

[32] Lamb J, Crawford ED, Peck D, Modell JW, Blat IC, Wrobel MJ, Lerner J, Brunet JP, Subramanian A, Ross KN, Reich M, Hieronymus H, Wei G (2006). The Connectivity Map: using gene-expression signatures to connect small molecules, genes, and disease. Science.

[33] Kuhn M, Szklarczyk D, Pletscher-Frankild S, Blicher TH, von Mering C, Jensen LJ, Bork P (2014). STITCH 4: integration of protein-chemical interactions with user data. Nucleic Acids Res.

[34] Seo HS, DeNardo DG, Jacquot Y, Laios I, Vidal DS, Zambrana CR, Leclercq G, Brown PH (2006). Stimulatory effect of genistein and apigenin on the growth of breast cancer cells correlates with their ability to activate ER alpha. Breast Cancer Res Treat.

[35] Shukla S, Gupta S (2007). Apigenin-induced cell cycle arrest is mediated by modulation of MAPK, PI3K-Akt, and loss of cyclin D1 associated retinoblastoma dephosphorylation in human prostate cancer cells. Cell Cycle.

[36] Cao HH, Chu JH, Kwan HY, Su T, Yu H, Cheng CY, Fu XQ, Guo H, Li T, Tse AK, Chou GX, Mo HB, Yu ZL (2016). Inhibition of the STAT3 signaling pathway contributes to apigenin-mediated anti-metastatic effect in melanoma. Sci Rep.

[37] Wang W, VanAlstyne PC, Irons KA, Chen S, Stewart JW, Birt DF (2004). Individual and interactive effects of apigenin analogs on G2/M cell-cycle arrest in human colon carcinoma cell lines. Nutr Cancer.

[38] Shukla S, Mishra A, Fu P, MacLennan GT, Resnick MI, Gupta S (2005). Up-regulation of insulin-like growth factor binding protein-3 by apigenin leads to growth inhibition and apoptosis of 22Rv1 xenograft in athymic nude mice. FASEB J.

[39] Chou TC (2010). Drug combination studies and their synergy quantification using the Chou-Talalay method. Cancer Res.

[40] Mine KL, Shulzhenko N, Yambartsev A, Rochman M, Sanson GF, Lando M, Varma S, Skinner J, Volfovsky N, Deng T, Brenna SM, Carvalho CR, Ribalta JC (2013). Gene network reconstruction reveals cell cycle and antiviral genes as major drivers of cervical cancer. Nat Commun.

[41] van Dam PA, van Dam PJ, Rolfo C, Giallombardo M, van Berckelaer C, Trinh XB, Altintas S, Huizing M, Papadimitriou K, Tjalma WA, van Laere S (2016). In silico pathway analysis in cervical carcinoma reveals potential new targets for treatment. Oncotarget.

[42] Chao A, Wang TH, Lee YS, Hsueh S, Chao AS, Chang TC, Kung WH, Huang SL, Chao FY, Wei ML, Lai CH (2006). Molecular characterization of adenocarcinoma and squamous carcinoma of the uterine cervix using microarray analysis of gene expression. Int J Cancer.

[43] Kim YW, Bae SM, Kim YW, Park DC, Lee KH, Liu HB, Kim IW, Jang CK, Ahn WS (2013). Target-based molecular signature characteristics of cervical adenocarcinoma and squamous cell carcinoma. Int J Oncol.

[44] Weldon CB, McKee A, Collins-Burow BM, Melnik LI, Scandurro AB, McLachlan JA, Burow ME, Beckman BS (2005). PKC-mediated survival signaling in breast carcinoma cells: a role for MEK1-AP1 signaling. Int J Oncol.

[45] Shukla S, Gupta S (2010). Apigenin: a promising molecule for cancer prevention. Pharm Res.

[46] Sak K (2014). Cytotoxicity of dietary flavonoids on different human cancer types. Pharmacogn Rev.

[47] Chuang CM, Monie A, Wu A, Hung CF (2009). Combination of apigenin treatment with therapeutic HPV DNA vaccination generates enhanced therapeutic antitumor effects. J Biomed Sci.

[48] Khan TH, Sultana S (2006). Apigenin induces apoptosis in Hep G2 cells: possible role of TNF-alpha and IFN-gamma. Toxicology.

[49] Zhang J, Liu D, Huang Y, Gao Y, Qian S (2012). Biopharmaceutics classification and intestinal absorption study of apigenin. Int J Pharm.

[50] Gradolatto A, Basly JP, Berges R, Teyssier C, Chagnon MC, Siess MH, Canivenc-Lavier MC (2005). Pharmacokinetics and metabolism of apigenin in female and male rats after a single oral administration. Drug Metab Dispos.

[51] Seyitoglu B, Yurt Lambrecht F, Durkan K (2009). Labeling of apigenin with 131I and bioactivity of 131I-apigenin in male and female rats. J Radioanal Nucl Chem.

[52] Casagrande F, Darbon JM (2001). Effects of structurally related flavonoids on cell cycle progression of human melanoma cells: regulation of cyclin-dependent kinases CDK2 and CDK1. Biochem Pharmacol.

[53] Choi EJ, Kim GH (2009). Apigenin causes G(2)/M arrest associated with the modulation of p21(Cip1) and Cdc2 and activates p53-dependent apoptosis pathway in human breast cancer SK-BR-3 cells. J Nutr Biochem.

[54] Iizumi Y, Oishi M, Taniguchi T, Goi W, Sowa Y, Sakai T (2013). The flavonoid apigenin downregulates CDK1 by directly targeting ribosomal protein S9. PLoS One.

[55] Maggioni D, Garavello W, Rigolio R, Pignataro L, Gaini R, Nicolini G (2013). Apigenin impairs oral squamous cell carcinoma growth in vitro inducing cell cycle arrest and apoptosis. Int J Oncol.

[56] Lee YY, Kim TJ, Kim JY, Choi CH, Do IG, Song SY, Sohn I, Jung SH, Bae DS, Lee JW, Kim BG (2013). Genetic profiling to predict recurrence of early cervical cancer. Gynecol Oncol.

