# Supplementary material for: Bioinformatics and in vitro experimental analyses identify the selective therapeutic potential of interferon gamma and apigenin against cervical squamous cell carcinoma and adenocarcinoma
Source: Oncotarget. 2017 May 2;8(28):46145–62. doi: 10.18632/oncotarget.17574 (PMC5542256; doi:10.18632/oncotarget.17574)
Supplement: Supplementary file 1 [file oncotarget-08-46145-s001.pdf]

Bioinformatics and *in vitro* experimental analyses identify the selective therapeutic potential of interferon gamma and apigenin against cervical squamous cell carcinoma and adenocarcinoma

SUPPLEMENTARY METHODS

Apoptosis detection

Apoptosis induced by IFNγ was determined using the Annexin V-FITC/17-AAD double staining assay (BD Biosciences) according to the manufacturer's instructions. Cells were treated with the indicated doses of IFNγ for 72 h. Then, the cells were trypsinized and analyzed on a flow cytometry (FACSCalibur, BD Biosciences). Data were analyzed by WinMDI 2.9 free software (Scripps Research Institute).

Western blot analysis

Cells were lysed in the M-PER Mammalian Protein Extraction Reagent (Pierce) containing 1x protease inhibitor cocktail (Roche) at 4°C for 30 min. Cell lysates were separated on a sodium dodecylsulfate (SDS)-polyacrylamide gel, and then transferred electrophoretically onto a Hybond-C Extra nitrocellulose membrane (GE Healthcare). The membrane was pre-hybridized in 20 mM Tris-HCl (pH 7.5), 150 mM NaCl, 0.05% Tween-20 (TBST buffer), and 5% skim milk for 1 h, and then transferred to a solution containing 1% bovine serum albumin (BSA)/TBST and a primary antibody and incubated overnight at 4 °C. After washing with the TBST buffer, the membrane was submerged in 1% BSA/TBST containing an HRP-conjugated secondary antibody for 1 h. The membrane was washed with TBST buffer, and then developed with an enhanced chemiluminescence (ECL) system (Bio-Rad Laboratories). Chemiluminescent signals were detected by the ChemiDoc Imaging System (Bio-Rad Laboratories).

SUPPLEMENTARY MATERIALS

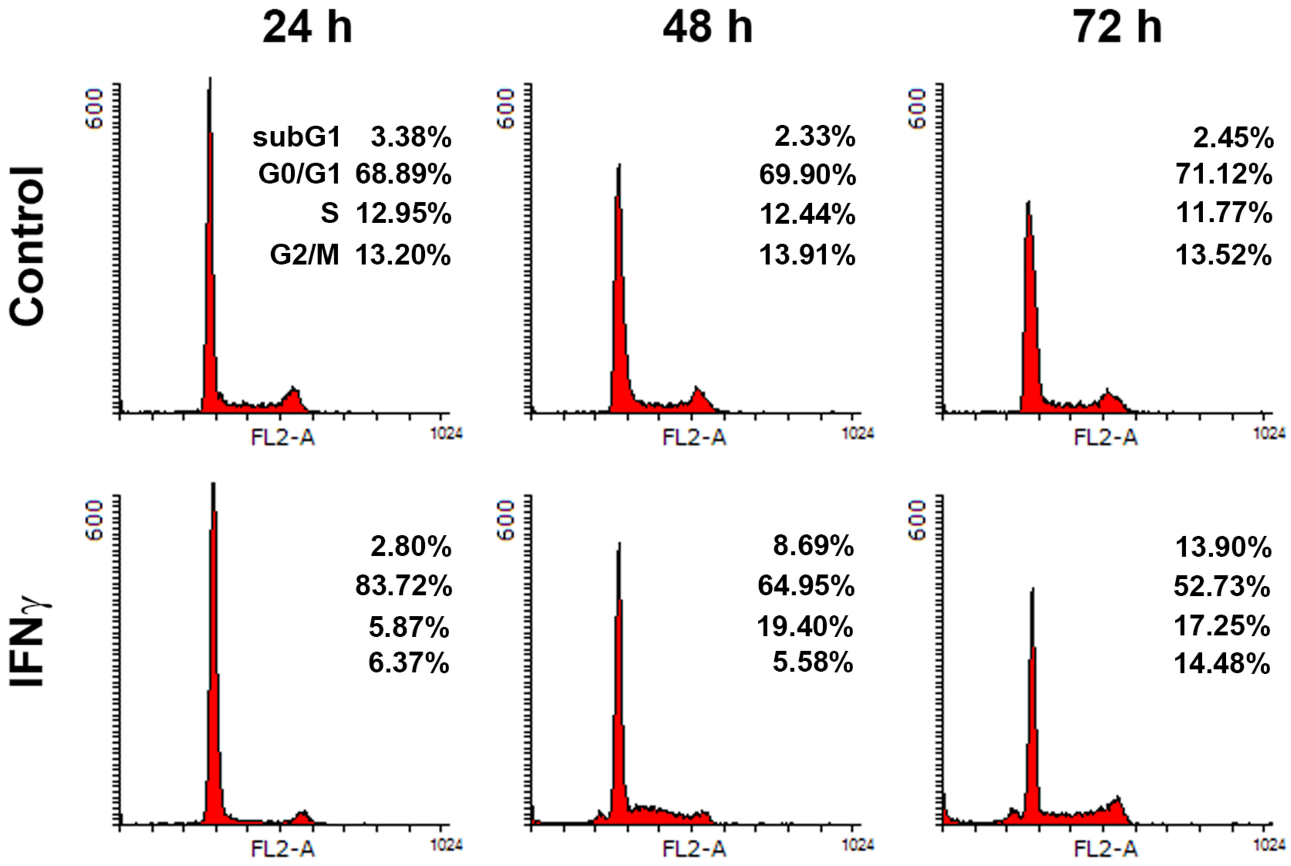

Supplementary Figure 1: Effect of IFNγ on cell cycle progression in SiHa cells. SiHa cells were treated with 100 ng/mL IFNγ for 24, 48, and 72 h, and then cell cycle distribution was examined by flow cytometry.

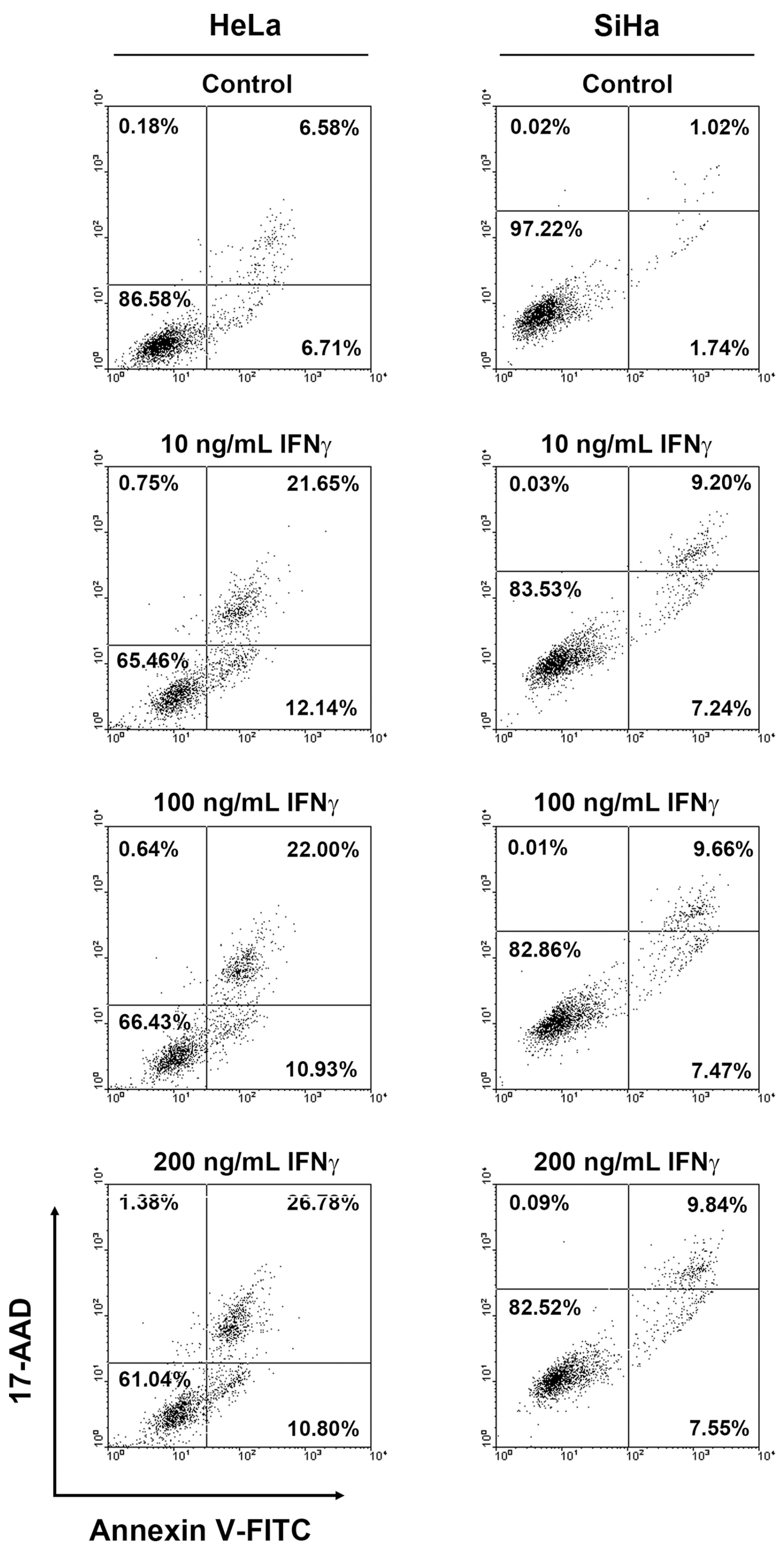

Supplementary Figure 2: Effect of IFNγ on apoptosis induction in HeLa and SiHa cells. HeLa and SiHa cells were treated with 10, 100, and 200 ng/mL IFNγ for 72 h, and then apoptosis was examined by the Annexin V-FITC/17-AAD double staining assay.

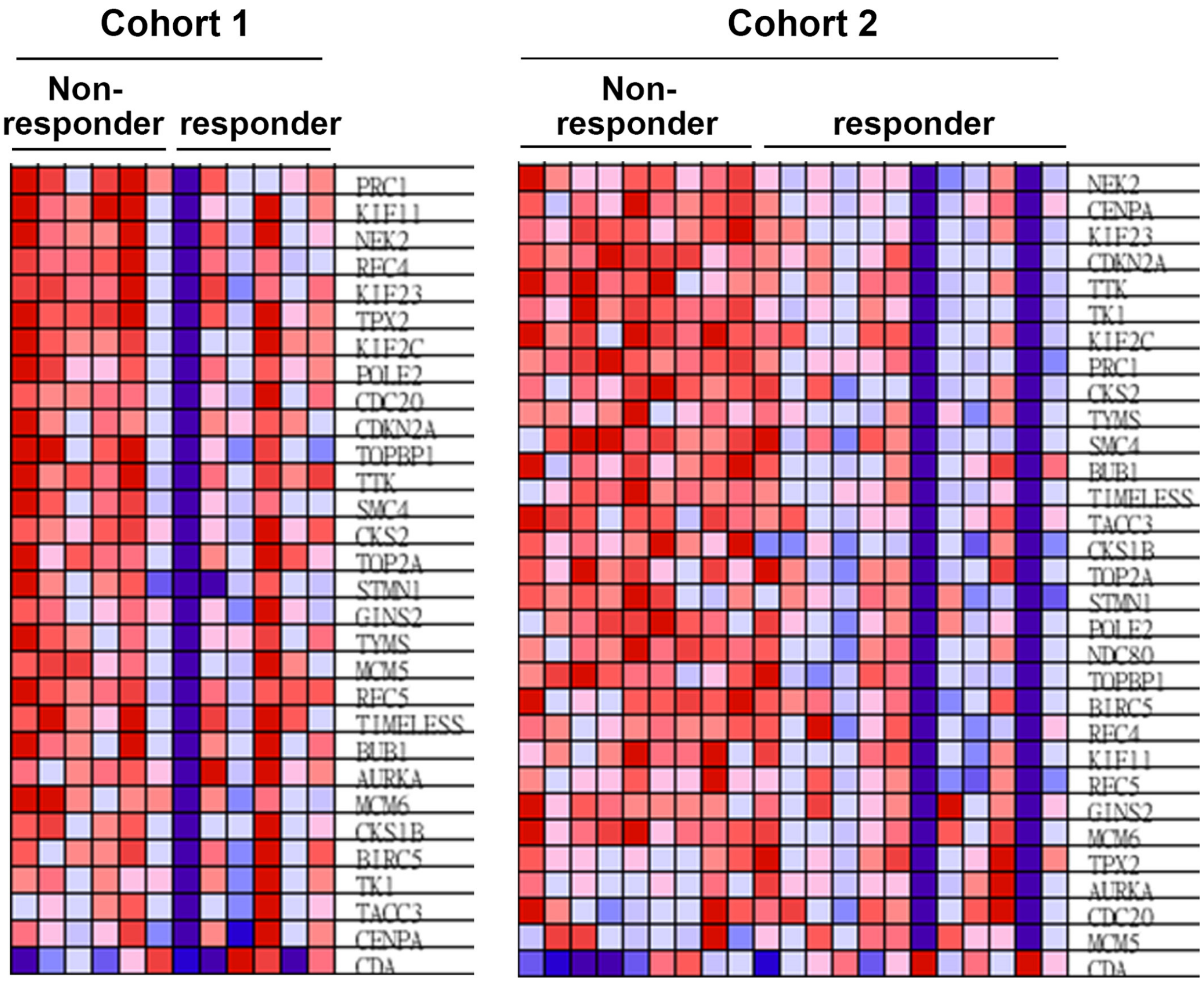

Supplementary Figure 3: The role of CxCa-Sig in clinical outcomes of cervical cancer patient. Heat map visualization for the GSEA results in Figure 4A.

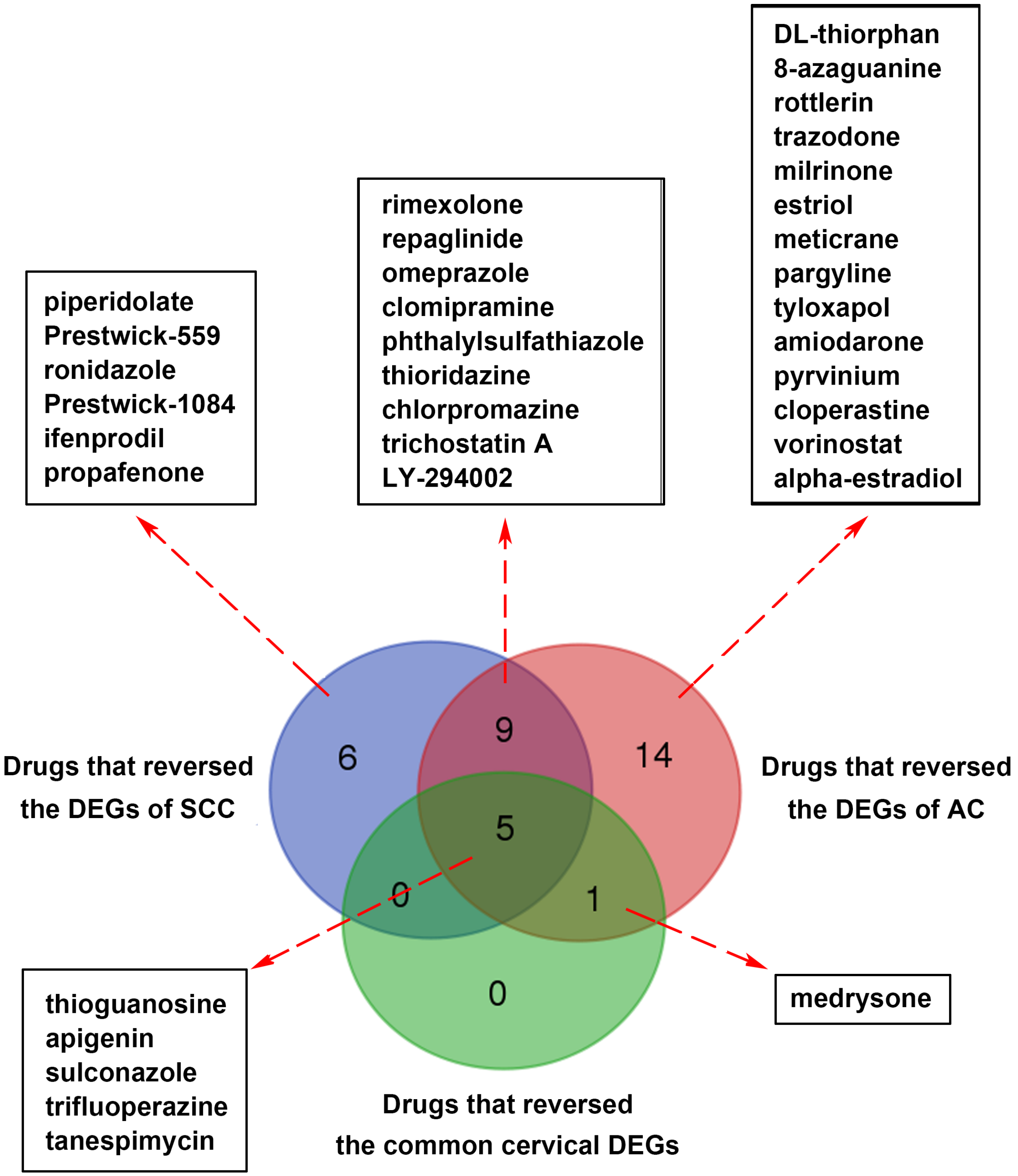

Supplementary Figure 4: The venn diagram for CMap drugs. The DEGs for SCC and AC were queried using CMap database. The predicted drug list was shown in Supplementary Table 4. The overlapping drugs among drugs that reversed the SCC-DEGs, AC-DEGs, and common DEGs were illustrated by the venn diagram that was generated using the Draw Venn Diagram (http://bioinformatics.psb.ugent.be/webtools/Venn/).

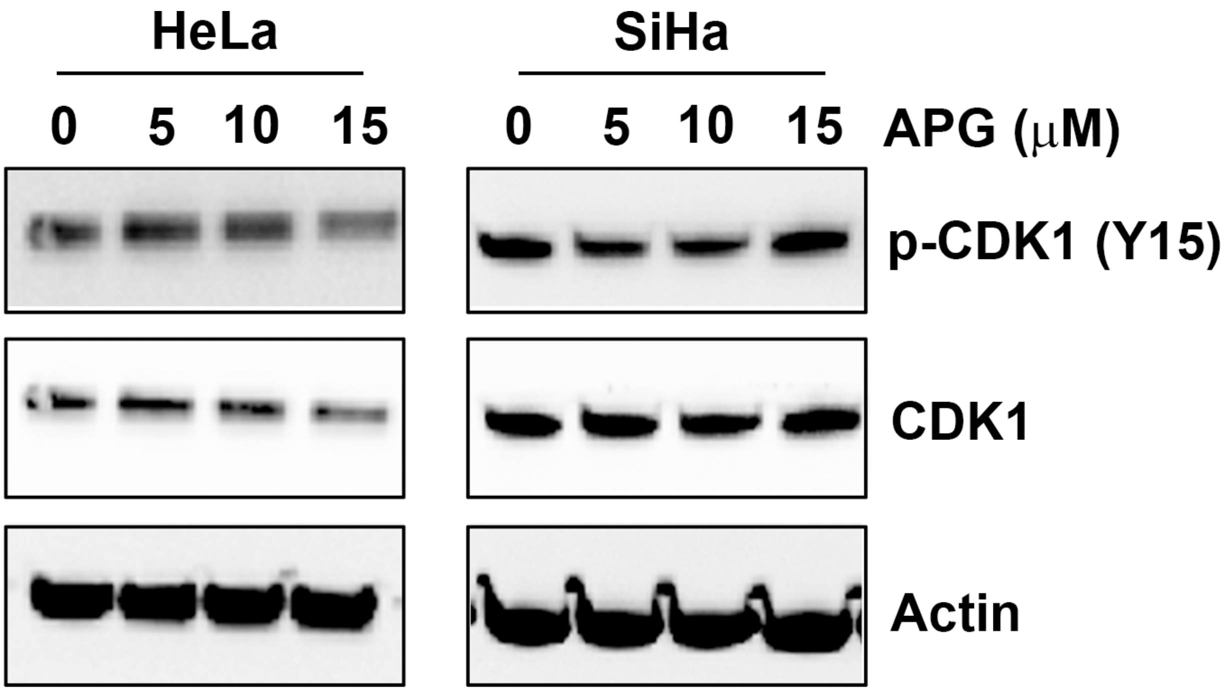

Supplementary Figure 5: Effect of apigenin on the inhibition of CDK1 in HeLa and SiHa cells. HeLa and SiHa cells were treated with 5, 10, and 15 μM apigenin for 24 h, and then the expressions of phospho-CDK1 (Tyr15; Y15) and total CDK1 were examined by Western blotting.

Supplementary Table 1: Top-50 up- and down-regulated genes used for pathway enrichment in Figure 1B

	IFNγ
Top-50 upregulated genes	HLA-DRB3, HLA-DRB1, CYLD, HLA-DRA, HLA-DRB4, CA12, HLA-DPA1, LCN2, ANKRD33, HLA-DQA1, S100A9, CTSD, LHFPL2, HCST, MARVELD1, P2RY11, TNFAIP6, CHPF, HLA-C, SERPINE2, LHPP, CACNG6, GRN, PLAC1, MSLN, VNN1, ITGA2, CTNNBIP1, HLA-G, RASD1, MKNK2, FAM20C, MT1G, KLF13, KLHL3, EVI2B, ARHGEF4, PARP6, TTC17, SCNN1D, HLA-DPB1, UBAP2L, HLA-A, PRSS27, LTBP4, ARSD, SPRED2, S100A8, DHDH, ATP11B
Top-50 downregulated genes	HMMR, CDCA8, SLC2A5, UBE2C, TOP2A, NEIL3, GPD1L, S100A10, CCNB2, PTPN6, CD36, KIF14, OSGEP, ACOT11, CENPE, KRT8, ABCA4, CTNNAL1, ACAA2, HSD17B8, CLTB, TPX2, FANCD2, GTSE1, FSTL1, PIAS3, CH25H, SLCO6A1, MIF, CDK6, NUSAP1, KIF23, FBXO5, LMNA, MRPL11, SLC9A3R1, COMTD1, PKP3, CCDC24, MAPK3, TAF5L, TK1, CCT2, ATPIF1, PDHB, PRC1, DCTN3, TOMM40, CDCA5, SPAG5

Supplementary Table 2: The differentially expressed genes (DEGs) of 7 cervical cancer patient cohorts for pathway (Figure 2A) and CMap (Figure 7) analysis.

See Supplementary File 1

Supplementary Table 3: The differentially expressed genes (DEGs) of cervical adenocarcinoma (AC) and squamous cell carcinoma (SCC) obtained from the dataset GSE39001 (GPL201).

See Supplementry File 2

Supplementary Table 4: The differentially expressed genes (DEGs) of cervical adenocarcinoma (AC) and squamous cell carcinoma (SCC) obtained from the dataset GSE39001 (GPL201).

See Supplementry File 3

Supplementary Table 5: Summary of predicted CMap drugs that reversed the gene expression profiles of SCC and AC. The differential expressed genes (DEGs) from SCC and AC were used to query the CMap. Only the results of CMap drugs with enrichment score < 0 and *p* value < 0.01 were considered significant. The predicted CMap drugs were ranked according their mean scores. The more negative in mean score indicates the higher possibility of CMap drugs to reverse the DEGs.

See Supplementry File 4
