# Supplementary material for: Bioinformatics and in vitro experimental analyses identify the selective therapeutic potential of interferon gamma and apigenin against cervical squamous cell carcinoma and adenocarcinoma
Source: Oncotarget. 2017 May 2;8(28):46145–62. doi: 10.18632/oncotarget.17574 (PMC5542256; doi:10.18632/oncotarget.17574)
Supplement: Supplementary file 2 [file oncotarget-08-46145-s002.docx]

**Table S2. The differentially expressed genes (DEGs) of 7 cervical cancer patient cohorts for pathway (Figure 2a) and CMap (Figure 7) analysis.**

| **GEO accession number** | **Upregulated genes** | **Downregulated genes** |
| --- | --- | --- |
| GSE7803 | CDKN2A, APOC1, NTS, FANCI, MCM5, SYNGR3, NUSAP1, CEL, HOXC6, APOBEC3B, FN1, AURKA, MUC1, ECT2, CXCL13, CCL20, LHX2, MCM2, SPP1, ISG20, DTL, NASP, WHSC1, KIF20A, MYBL2, NCAPH, RFC4, RPL39L, PLOD2, WARS, CKS1B, ASPM, HMMR, ENO2, EMC1, CXCL8, IFI44L, TOP2A, CXCL1, NEK2, TIPIN, APOE, KNTC1, ITGA6, TRIO, LAMP3, MLF1, CENPN, KRT17, TRIO, GAD1, SLC6A8, TK1, UCP2, FOXM1, PRC1, ACOT9, AIM2, RIBC2, TNFSF10, E2F1, EPCAM, MELK, CDK1, KIF4A, CFHR1///CFH, MAGOHB, ALCAM, SOX4, CEP55, SYCP2, CENPF, IL32, KCTD7, KIAA0101, MET, PCNA, PLOD2, LYN, MRE11A, SYT17, ATP2C1, NUSAP1, SMC4, TAZ, DSG2, IFI16, SMC4, SRSF10, HP1BP3, SAT1, TOP2A, GINS1, HJURP, MMP12, ACKR3, BIRC5, FANCI, GINS2, CCL18, MAGOH, ANKRD36B, AURKA, RRM2, SHARPIN, SPAG5, RECQL4, ADAM8, EFNA1, IGF2BP3, TTK, E2F3, EIF2S2, FOXD1, NUP93, SAC3D1, SNAPC4, WDHD1, ISG20, PLSCR1, BTN2A1, IGF2BP2, MCM7, RAD51AP1, NDC80, TYMS, ATP2C1, SIVA1, NUDT1, NUP210, TMEM194A, CDC45, HELLS, PPM1A, SLC16A1, TGM2, BRCA1, DTYMK, PLSCR1, CCNF, VSIG4, FARS2, IFI16, RSRC1, TP53I11, CDKN2A, HIST2H2AA3, INHBA, LAPTM4B, MARCKSL1, STAT1, DFNA5, ECI1, H2BFS, KIF23, LMNB1, CDC20, HOXC10, MAGEF1, CDKN2C, U2SURP, CIZ1, MKI67, TFDP2, HMGB3, TFRC, ATP13A3, NCF1C, CCNE2, FEN1, ANKRD36, LAPTM4B, MAD2L1, SOX4, CTSZ, GZMB, HOXD3, IL1A, LYN, RPP25, SNX10, TACC3, APEX2, CKS2, POLQ, USP18, BST2, IFI16, KIF20B, RMI1, SLC25A36, STMN1, TOPBP1, UPF3B, MIS18BP1, BIRC5, GMNN, LOC100507619, SLC16A3, TNFSF10, HLTF, MPHOSPH9, PLK4, XRCC4, CCR1, CELSR3, GOLIM4, MOCOS, POLA1, POLQ, SMC2, LDLR, NCAPG, PRR5-ARHGAP8///ARHGAP8, SHCBP1, VEGFA, VRK1, COX17, HSPBAP1, KIF14, LIMK1, TFRC, ZWINT, CENPF, MCM6, PBXIP1, PLAU, PLCE1, ARHGAP4, CENPA, CTSL, NUP210, FANCA, ITGB4, MGA, MTHFD2, PARP2, TPP1, ZNF668, ADA, ATP2C1, B4GALT4, CDC25A, E2F8, LAPTM5, NCAPG, PCTP, RNFT2, SLC15A3, U2SURP, MAGEA2B///MAGEA2, PCDH12, PRKCI, RAD51, CCNE1, EPRS, HMGB2, SLC16A1, ACYP1, ATP5C1, CHAF1B, DLGAP5, HEY1, KIFC1, NCF2, OIP5, PSRC1, PTTG1, SLC35G2, SMC2, TPX2, CALML4, HIST1H2BH, NCF1C///NCF1B///NCF1, EZH2, HIST1H3H, KIF18B, MBD4, TMEM106C, ABCG1, CCNF, CSE1L, INTS7, LAMC2, STAG1, ATP2C1, CCNB1, DONSON, ECE1, MCM10, PSMC3IP, RACGAP1, RANBP3, RBM41, CDK1, MKI67, NCOA3, SYNJ2, DNA2, GALNT2, HS6ST1, LOC100510692///NAIP, WRAP73, APOL6, BACE2, C1QB, CENPM, CYFIP2, KIF2C, MIR10B///HOXD4///HOXD3, RFC5, RRM2, YEATS2, SEP9, DNMT1, MTF2, PHIP, TIMELESS | ITM2A, LPAR6, PDZRN3, PPIL2, SASH1, SCNN1B, TNXB///TNXA, AQP1, GDF11, KRT32, LIMCH1, OR2F1, AHNAK, FAIM3, MREG, NSG1, SLIT2, TNXB///TNXA, VPS53, ATP10D, CCND2, GAB2, SLC18A2, CTNNAL1, NAV3, PHYHIP, SMAGP, BMP2K, CYP3A5, DKK3, EIF4A1, RHBDF1, EFNB2, PDE2A, RAPGEFL1, DIAPH2, TRAV12-2, CFAP69, CX3CR1, GFOD2, SASH1, SC5D, ADH6, ANXA1, ITM2A, LDOC1, PELI1, CYP2C9, LGI2, PDCD4, PLA2G3, PPL, S100A12, BTRC, IL1RN, PRDM12, PROS1, PTGDS, TUBA1A, ITPR2, MSMO1, ODC1, CA12, PLAGL1, TRPS1, CD24, CSTB, MEIS2, CD24, FZD1, IRS1, KLK13, LPIN1, MADCAM1, DSG3, EFNB2, GATM, GULP1, GULP1, LANCL1, SYNGR1, ZNF750, CADM1, ZNF185, HIGD1A, SH3BP5, KANK1, MYOZ3, TGM1, WIF1, ACPP, PEG3, S100A14, VSNL1, ZNF426, CYP2C18, FCER1A, PLAGL1, CERS4, PITPNA, SOX9, GEM, MGLL, ASAP3, CYP2C18, ELOVL6, EVPL, ME1, SULT2B1, FKBP1B, CD24, HLA-DQB2, RND3, SERPINI1, DCLK1, HBB, PDLIM2, KRT2, SERPINB1, SPINK2, TP53AIP1, ABCA8, DEPTOR, DUSP1, HBEGF, EPB41L4A, HBB, TGFBR3, C2orf54, KCNK7, MPZL2, EFNA5, KLF4, PRSS3P2///PRSS1, GNG11, MIR22HG, KLK12, TNXB///TNXA, ALOX15B, METTL7A, NEBL, FLRT2, ME1, TUBB2A, ZNF91, DEGS1, IGFBP5, WNT5A, DCN, ECHDC3, CYP3A5, KIF1C, C1orf116, SFTPD, PTGER4, TST, DPP4, LYPD3, PI3, ZBED2, GAS7, GPX3, NUAK2///AKIP1, DCN, MALL, NOD2, PRSS2, DCN, DSC2, CCND1, EIF1, CD24, CWH43, PDGFD, SPRR2B, VSNL1, CYP3A7, EPB41L3, DCN, C1orf116, DIO2, EMP1, CYR61, FGFBP1, LOR, CALML3, NSG1, SLC24A3, DKK3, SPON1, DIO2, GJA1, TP53I3, PITX1, SLC24A3, SCGB2A1, COL9A1, UPP1, DNAJB1, GPX3, PTGDS, CDA, AREG, KLF4, GLTP, TMPRSS11E, AR, FCGBP, GSTA4, IGF1, PTK6, TTC39A, NDN, RRAGD, SERPINB1, PRSS3///PRSS2///PRSS1, PTGDS, ALOX12B, ARC, CXCR2, HSPB8, KLK10, SERPINB2, EPB42, SLC16A7, ISL1, PRSS3, GYS2, THSD4, MAOB, SPRR1B, OCA2, CLDN8, IL18, TMPRSS11D, FGFR2, DSC2, KLK7, HPGD, SPARCL1, CRABP2, NSG1, PRSS3, ZSCAN18, AKR1B10, GREB1, CXCL12, PI3, ID4, RHCG, EMP1, HPGD, LGALSL, EREG, BBOX1, CYP3A5, FAM107A, FOSB, SPRR1A, PAMR1, HPGD, KLK11, SCEL, HPGD, EMP1, OLFM4, PPP1R3C, ENDOU, ESR1, IL1R2, IVL, SOSTDC1, CFD, CWH43, CRYAB, ALOX12, KRT13, SLURP1, EDN3, HOPX, SPINK5, CRCT1, CRISP3, IL1R2, DSG1, SPRR3, KRT4, MAL, UPK1A, KRT1, KRT4, CRNN |
| GSE29570 | MMP12, SYCP2, SMC1B, MMP1, CDKN2A, SPP1, FAM111B, TPX2, CDC6, IDO1, DTL, NUF2, CXCL10, TOP2A, MELK, STIL, ASPM, CLSPN, CENPF, ANLN, DLGAP5, MMP3, CDK1, KIAA0101, NUSAP1, ECT2, FOXM1, MIR15A, EXO1, KIF4A, KIF2C, CCNB2, MMP10, CCL18///CCL18, E2F7, AIM2, MCM2, CASC5, KIF14, ARHGAP11A, BRIP1, LAMC2, CDKN3, TYMS, ASF1B, CLDN1, MMP13, CENPI, CXCL8, AURKA, CCNE2, KIF11, PRC1, UBE2T, POLQ, APOBEC3A_B///APOBEC3B, KIAA1524, KIF23, CENPK, FANCI, WDHD1, NEK2, CDH3, ITGB6, LCORL///NCAPG, RFC4, MKI67, APOL1, CEP55, HBB, KIF20A///CDC23, TTK, DEPDC1, MYBL2, CCNB1, PLK1, FAM72A///FAM72D, GINS1, RAD51AP1, PAK6///BUB1B, ABCA13, CDC7, RRM2, BUB1, MCM4, DSG2, IQGAP3, PBK, PTTG3P///PTTG1, KIF18A, CHI3L1, ORC1, SHCBP1, WDR76, KIFC1, CXCL9, KNTC1, MCM5, GBP5, HELLS, HIST1H3B, HIST1H3F, ORC6, PLOD2, KRT17, LMNB1, SPAG5///SPAG5, TICRR, HORMAD1, CDCA2, POLE2, KIF15, TDRD6///PLA2G7, CDC45, CDC20, CXCL2///CXCL1, DIAPH3, EGLN3, KPNA2, MCM8, ATAD2, XRCC2, RPL39L, CHEK1, FAM72A///FAM72D///FAM72B, TIMELESS, MOCOS, NETO2, UBD, DNA2, NCAPG2, SMC4, TK1, ESCO2, LAMP3, NCAPH, PLAT, SPC25, TFRC, TRIM59, USP41///USP18, BRCA2, HIST2H4A, HIST2H4B, NUP210, PLK4, UBE2C, EZH2, LIPH, PRR11, SLFN13, TCF19///TCF19, ATAD5, C3orf52, CENPO///ADCY3, CKS2, HIST2H3D///HIST2H3A///HIST2H3C, MEST, PLAU, HBA2///HBA1, SKA3///MRPL57, BLM, SGOL1, UHRF1, BRCA1, FCGR3B///FCGR3A, GEN1, IRF1, OAS3, PCNA-AS1///PCNA, CKS1B, ICAM1, RAD51, FANCA, HIST1H2AB, HIST2H4B///HIST2H4A, MEI1///MEI1, HCAR2///HCAR3, HIST1H3G, IL1B, MMP9, MYO3A, RDH10, CMC2///CENPN, STMN1, PARP14, HIST1H1B, NDC80, ALG1L, IFI30, GMNN, SLC2A1, CENPE, MCM6, RNF212, CA9, CENPW, FEN1, ARHGAP11B, DHFRL1///DHFR, HIST1H2BH, LAMB3, NEIL3, RACGAP1, TRIP13, CENPU, DEPDC1B, DSN1, HIST1H3H, HJURP, LOC1720, CDK2, PRIM1, RAD54L, UBE2S, CKAP2, CKAP2L, DTX3L///PARP9, GAS2L3, RIBC2, RNASEH2A, CDC25C, CDCA5, HIST1H3H///HIST1H2AI, HIST1H3I, HMMR, IGHG4///IGHA2, NEDD4L, NQO1, TAP1///PSMB8-AS1, ZWINT, ADAMDEC1, HIST1H2BF, ITGA3, MCM7, FANCD2, IL1A, TMPRSS4, WARS, ZYG11A, CA2, CYP4F11, MCM10, STRIP2, CDCA8, GGH, MCM3, TOPBP1, CENPA, E2F8, ITGA2, OCIAD2, TACC3, TMC7, TNFSF10, C1orf112, GINS2, ATP13A3, HLTF, PSMB9, RBL1, SDC4, CHAF1B, HENMT1, MAD2L1, OAS2, PARP9, ACTL6A, AURKB, BAK1, CDT1, OAS1, TMEM194A, BORA///DIS3, CD274, IFI6, MGME1///MGME1, NUP62CL, TMEM171, CCNF, FBXO5, LCTL///ZWILCH, LY6K, OTUD7A, BAIAP2L1, HIST1H2BJ, NCAPD2, OIP5, PLAUR, VEGFA, AQP9, GINS4, MMS22L, NEFH, SLC6A10P///SLC6A8, CCNA2, EFNA1, FANCB, KIF4B///KIF4A, PLEK2, ZNF367, HIST1H4J///HIST1H4K, ITGB4, LOC100131860, MTHFD1, SMC2, STAT1, BIRC5, IL32, LYN, MSL3P1, RFC5, CCDC88C, CDC25B, CIT, PKMYT1, TMEM106C, HIST1H3A, HIST1H4L, IFIT3, IL12RB2, RMI1, CXCL13, LOX, POLA2, CCNE1, HCP5, MB21D1, NRIP3, SNORD30, DNMT1, H2AFZ, MTFR2, HIST1H3J, HK2, NDC1, RFC2 | AREG, CCL21, CD36, GPR124, KANK2, MIR218-1, PCSK5, PLEKHH2, TSC22D3, ANTXR2, CD248, EPB41L4A-AS1, FREM1, GREB1L, NDP, PPFIA2, SGCD, SLURP1, ZNF208, ZNF320, ARHGAP10, ATP1B2, CAV1, DAB2, LGI4, METTL7A, PWARSN///SNORD107///SNURF///SNRPN, ROR1, SPG20, ZNF426, ZNF516, ABCD2, ECM1///ECM1, SNORD116-15, TCEAL1, ABCB1, C16orf45, GPM6B, MRVI1, SEMA3E, SRPX, STARD9, TBX3, C8orf4, GPRASP1, LAMB2, NPR2, SCUBE2, SLC2A13, SYTL4, FHL5, KLHL3, MOXD1, REV3L, TPTEP1, ZNF506, EPS8, MGC24103, NDRG2, NR2F1, PAM, PCDHB15, PDE8B, SASH1, SNCAIP, BARX2, FAM198B, HSPB8, LHFP, SDPR, SMOC2, TEK, VIM, VIPR2, CACHD1, CCBE1, IGFBP6, LSAMP, PCOLCE, SNORD116-29, CNTN1, HLF, PDE3A, SEMA3D, ZNF429, ABCA10, AOX1, ARHGAP20, CHRDL1, CXorf36, DES, NR3C2, RERG, SNORD116-20, SNORD116-23, TACR1, TENC1, TRPS1, CPA3, F13A1, MATN2, PRSS23, RGS2, SCGB1D2, SCN7A, SLC16A7, SULT2B1, AASS, JUN, KCNMA1, LINC01140, PIK3R1, SEMA5A, SLIT3, SPRY1, SYNE1, TGFB3, TIMP2, DNM3, ECM2, ELTD1, FLJ38379, HTR2B, KLK10, LTBP1, PLCB4, PRSS27, RAET1E, RGS1, RPTN, SNORD116-22///SNORD116-21///SNORD116-20///SNORD116-18///SNORD116-17///SNORD116-16///SNORD116-15///SNORD116-14///SNORD116-19///SNRPN, SORBS1, ZNF493, RGN, ATP6V1C2///PDIA6, CALCRL, HSPB6, KLK6, SNORD116-4///SNRPN, BEX4, CCL15-CCL14///CCL15///CCL14, FAM189A2, FBLN1, FERMT2, MCOLN3, NBEA, SHISA6, SLC24A3, CYP4F22, EDN3, FAXDC2, MS4A2, NCCRP1, RBMS3, SELP, ZFPM2, BNC2, GLT8D2, ITM2A, MYLK, NR4A3, PDGFRB, WISP2, CACNA1D, CLIC3, CNN1, CPEB2, CSRNP3, GEM, ISLR, KLF4, KLK11, MMP2, NAALAD2, PODN, PPL, PRUNE2, RBP7, RBPMS, CDR1, CLDN17, CPXM1, CYYR1, GSTA4, ITGA9, MUM1L1, NLGN1, PTPRD, PXK, ZNF676, FGF14, FMOD, MYCT1, NRN1, ZNF43, ZNF626///ZNF93///ZNF253, ANKRD35, DIO3, FAM3D, SNORD116-14, TSPYL5, ZNF521, AGPAT9, CC2D2A, DDR2, DSEL, FGF9, KLHDC1, LMO3, OLFML3, PARM1, SLC5A1, CTTNBP2, DMKN, LONRF2, PLN, RECK, SSPN, CNTN4, DKK1, GRIA3, KGFLP2///KGFLP1///FGF7, SEPP1, SLC27A6, SNORD116-24, ZEB1, CPQ, NPY1R, THSD7A, ZBTB16, ZNF254, ZNF385D, AKT3, CRABP2, ITGBL1, LRRK2, METTL24, PRRX1, RNF150, IL18, PRKG1, TFPI, XG, ENPP3, GRIA2, SPON1, ADAMTS5, GJA1, LDOC1, MPPED2, NDNF, PTGIS, BNIPL, DPP4, EMX2, GNG11, GSTM2, MPDZ, PWAR6, ADAMTS1, COL21A1, DEPTOR, F10, FBLN5, HOPX, KGFLP1///FGF7, MRGPRF, NR4A1, PLTP, ANKRD31, ME1, NOSTRIN, OMD, PCDHB5, PI15, PRELP, ADAMTS19, FAM13C, IL1R1, RHOJ, ZNF415, EBF1, EGR1, MAOB, LRRC17, NT5E, PPAP2B, SOX5, ABCA9, ABCC9, LPAR1, PCP4, TIMP3, DACH1, IVL, RPS6KA6, SNORD116-6, SOSTDC1, TNXB///TNXA, ASPA, NCAM1, PTCH1, MIR675///H19, PGM5, PTGER2, ALDH1A1, CILP, FBN1, FXYD1, PDE1A, PI16, RGS22, CRISPLD2, FLG, KIT, MGP, RFTN2, FAM171B, LMOD1, PCDHB4, PRICKLE2, RGS7BP, ZNF91, ALDH1A2, CDA, LPHN3, MICU3, TMEM132C, ZFHX4, ZNF626, MASP1, PDGFRA, PREX2, CTGF, GUCY1A2, SNORD113-3, ANO4, EDNRB, FRZB, ARMCX1, FLRT2, NAP1L2, RBM24, ZEB2///ZEB2, ZNF483, CD34, FIBIN, FAT4, JAM2, PGM5P2///PGM5, RNF180, SGCE, AQP1, EPHA7, PTN, BBOX1, GPR133, GREB1, KCNIP4, RDH12, SNORD114-3, MEG3, PCDH18, SNORD64///PWAR5, PMP22, CAB39L, CFD, CXCL14, LY75-CD302///CD302///CD302, ZNF737, JAZF1, LDB2, ANKFN1, ARHGAP28, CYBRD1, HAND2-AS1, MYH11, PDZRN3, PRSS3P2///PRSS3///PRSS2, PTGDS, SHC3, MYZAP///GCOM1///POLR2M, SLC16A9, SNORD116-9///SNORD116-8///SNORD116-7///SNORD116-5///SNORD116-3///SNORD116-1, CCDC80, ENPP1, RORB, SNORD116-1, CYP1B1, EPHA3, GPX3, GULP1, ENDOU, LCE3D, SULT1C4, PKHD1L1, ABCA6, BCHE, SNORD116-9///SNORD116-8///SNORD116-7///SNORD116-6///SNORD116-5///SNORD116-4///SNORD116-3///SNORD116-2, SERPINF1, RUNX1T1///RUNX1T1, CPED1, EDNRA, EREG, FGF7, MIR145, MITF, SDR9C7, TMEM47, TSTD2///TMOD1///TMOD1, LRCH2, MUC15, LOC100288114///ZNF667-AS1, DCN, KLK13, DCLK1, EYA1, MAPK10, SVEP1, PRLR, LRFN5, CTSK, FHL1, MAMDC2, TRPC1, CXCL12, SNORD116-21, TSPAN7, ANK2, EMP1, OSR2, ITGA8, LAMA2, NAP1L3, PDGFD, PRSS3///PRSS2, PDK4, SPARCL1, ACSS3, SFRP1, ABCG2, IL36A, TGM3, NOVA1, ABI3BP, C7, FGF10, MFAP4, SYNPO2, SNORD114-2, DPT, OLFML1, FABP4, KRT78, SLC18A2, AR, GYS2, PDE5A, FOS, DUSP1, CYR61, RHCG, MIR99AHG, MMRN1, SH3BGRL2, KLK12, TGFBR3, FAM25G///FAM25C///FAM25A, PAMR1, EMCN, THSD4, SPRR1A, ESR1, LYVE1, TRHDE-AS1///TRHDE, KRT13, PGR, KRT1, SNORD113-4, ABCA8, A2ML1, SCEL, EPGN, HPSE2, TPRG1, C1QTNF7, SLIT2, CWH43, LIFR, CGNL1, IGF1, FOSB///FOSB, IGFBP5, CRYAB, GSTM5, HPGD, TMPRSS11A, COL14A1, UPK1A, LOC441178, TMPRSS11E, OGN, FMO2, KRT4, SBSN, SPRR3, KRTDAP, APOD, PPP1R3C, SFRP4, SPINK5, CNFN, MUC21, CRCT1, ALOX12, MAL, DSG1, CRNN, TMPRSS11B, CRISP3, SPINK7 |
| GSE39001  (GPL201) | RRM2, NUSAP1, MMP12, TYMS, CDC20, PRC1, UBD///GABBR1, KRT17, RFC4, CDKN2A, TOP2A, CDKN3, ZWINT, SYCP2, CKS2, CDK1, MMP1, S100P, SMC4, GAPDH, UBE2C, MCM5, MCM2, HBA2///HBA1, KRT7, APOBEC3B, CCNB2, GMNN, CXCL10, PTTG1, AURKA, MEST, CENPA, CXCL9, TPX2, KIF20A, BUB1B, PLOD2, NEK2, MSLN, UBE2S, MAD2L1, LAMP3, RACGAP1, PLSCR1, GINS2, CDC7, HLA-DRA, EZH2, PBK, PCNA, TOPBP1, HLTF, SPP1, KIF4A, NCAPG, IL32, ISG15, SDC4, IFI27, HSPA8, CKS1B, BIRC5, PSMB9, TTK, TACC3, NDC80, MCM6, SLC38A1, CXCL13, MUC1, IFI44L, IFI30, FEN1, KIF11, H2AFZ, AIM2, ILF2, CA2, RFC3, RFC5, LAMB3, TIMELESS, TK1, ATP2A2, CENPE, RYR1, PDZK1IP1, F11R, LSM4, IDH2, MX1, KRT19, MRPS12, HN1, HOXC6, STIL, ENO1, OAS3, CDH3, GGH, HLA-DRB4///HLA-DRB3///HLA-DRB1, PLAT, PSMB8, DHX15, HPRT1, CCNA2, STMN1, CCT2, HSP90AB1, RAD51AP1, HNRNPF, STAT1, OAS1, KPNA2, SCD, RNASEH2A, HBB, YWHAE, PNP, CDC25B, DNMT1, SMC1A, NEFH, TRIP13, SELT, CTSS, MCM3, PSMC3, ITGB4, KRT8, LAPTM4B, SLBP, KIF15, TGFBI, GMPS, ASS1, RANBP1, TNFSF10, IRF1, POLE2, C1QB, BARD1, EIF2S2, AP2S1, DSG2, EFNA1, DEK, PLP2, IRF7, GNB1, TAGLN2, HAT1, TUBG1, CCNE2, DDX39A, SOX4, CDC45, RPN2, LIG1, PRIM1, IMPA2, PLAU, BUB1, BTN3A2, POLE3, NUP107, ISG20, NMI, ELF3, CA9, BST2, CDKN2C, ADAMDEC1, TAPBP, MSH2, YWHAH, ELF4, GCH1, RAN, CTPS1, ACTL6A, SHMT2, WHSC1, LAPTM5, SPAG5, FZD6, MTHFD1, AP2M1, HMGB1, HSPA2, LAP3, SLC6A8, DDOST, SNRPD1, USP1, KIF2C, NCK1, SLC25A5, CTSC, HLA-B, PSMB2, MICB, STAT3, ITGB3BP, MLF1, HIST1H1C, LDHA, CSNK1D, PHLDA2, ACOT7, HNRNPAB, SDC1, GJB3, HSPA1B///HSPA1A, CD164, LAMC2, PPP1CA, PSMA7, HLA-G, CAPG, PDXK, PAFAH1B3, RAD23A, H2BFS, VAMP8, LMNB1, KIF22, PLAUR, ITPR3, PSMD8, ERBB3, ARPC5, RRM1, DCK, CIB1, BLM, SERF2, NUP62, TNNT1, RC3H2, YWHAZ, RUVBL1, CDK2, USP18, TYMP, TMX1, PHB, H1FX, INHBA, PSMD11, MTF2, SNRPB, MKI67, BRCA1, FAT1, PSMD1, VDAC1, HNRNPU, RAD21, LSR, VRK1, KIF23, CYBA, LSM2, PDCD10, PRKCI, ATP1A1, IL10RB, SLC20A1, ALG6, PSMD12, ACTR2, MSH6, FCGR3B, GDE1, PTPRF, ZNF148, MYBL2, PKM, ICAM1, HLA-DRB1, SLC16A1, UCK2, EIF4EBP1, PRDX1, XPNPEP1, RAD51, CEBPG, HMMR | CDO1, RAPGEF3, NR3C2, CAV1, FBLN2, ACPP, ZEB2, TGFB3, SMARCD3, NRCAM, RCAN2, QPRT, FYN, GAS7, CPA3, RCBTB2, ZFPM2, PENK, PLAGL1, NSG1, WASF3, ACAT1, MRC2, DSG1, CACNA1D, PDGFRB, GAS6, PDLIM2, ENDOU, SLURP1, NUCB2, WFS1, LAMC3, HTRA1, SLC5A1, STX18, TACC1, IRS2, RCAN1, SALL1, CH25H, TPST1, TST, DPT, ARHGAP6, PCDHGA1///PCDHGA2///PCDHGA3///PCDHGA4///PCDHGA5///PCDHGA6///PCDHGA7///PCDHGA9///PCDHGA10///PCDHGA11///PCDHGB1///PCDHGB2///PCDHGB3///PCDHGB5///PCDHGB6///PCDHGB7///PCDHGC4///PCDHGC5///PCDHGA12///PCDHGA8///PCDHGB4///PCDHGC3, ENTPD3, RRAGD, REV3L, CDA, HOXA10, BBOX1, MAF, PDGFRL, UPK1A, ATP1A2, BCL2, COL6A3, CNN1, ZBTB16, LAMB2, SNCAIP, WFDC1, LHFP, DPP4, ME1, KLHL3, ECI2, SATB1, F10, CRTAP, THBS2, IL33, STC1, PTGIS, NELL2, EDNRB, PEBP1, COL1A2, LAMA2, AQP1, PLTP, BDKRB2, CHN2, CITED2, MXRA7, CHST7, RGS2, GSTA4, PTGER2, RGS1, FBXL7, DCLK1, ADIRF, EPHB6, SCUBE2, NBL1, RECK, DACH1, THBD, SLC39A8, COLEC12, SOX17, TCEAL1, KAT2B, KIT, FMOD, MAOB, PKIG, DIO3, CX3CR1, KCNMA1, IL20RA, PRR16, ALPP, FGF9, LDOC1, ABCG2, FGFR1, RBPMS, SEPP1, DKK2, LMOD1, PAM, IVL, ALDH1A3, PMP22, HEPH, ADRB2, FRZB, PCSK5, CIRBP, GATA2, EMP1, NT5E, FBLN1, EFEMP2, FAM107A, IRS1, SORL1, TGFB1I1, CCL15-CCL14///CCL14, PECAM1, FGF13, TUBA1A, PPAP2A, RHOB, CDC42EP3, MMP2, GSTM5, TGM2, HP, PCOLCE, ADAMTS5, RUNX1T1, GHR, NOVA1, SORD, PRSS23, SGCE, FZD10, SLC18A2, DAB2, TNS1, EGR1, LEPR, SLIT2, TRH, FABP4, SLPI, SCNN1B, SOCS2, S100A4, C4BPA, PPAP2B, NCAM1, ECM2, DIO2, GRIA2, CLU, SERPINF1, SSPN, PDGFD, ADAMTS1, COL16A1, CRYAB, ALDH1A2, MITF, SLC24A3, FXYD1, ISLR, KRT1, GNG11, KCNJ15, JAM2, IGFBP6, FOSB, CTSK, CLEC3B, DKK1, PTGS2, DUSP1, EDNRA, NAP1L3, SNAI2, LTBP1, IL1R1, CYP1B1, GJA1, FOS, CILP, CYP2B7P///CYP2B6, HTR2B, NDP, SPARCL1, LRRC17, ADRA2A, ITM2A, FBN1, FCGBP, TGFBR3, MATN2, RAI2, COX7A1, MPPED2, EDN3, LDB2, AMY2B///AMY2A///AMY1C///AMY1B///AMY1A, NR2F1, SRPX, MYLK, ABCA8, BCHE, PPP1R3C, ANK2, ALOX12, OSR2, PDGFRA, CYR61, DKK3, IGF1, ID4, SFRP1, NDN, PCP4, SPINK5, DEFB1, FLRT2, FHL1, PTGDS, TFF3, CRCT1, SCGB2A2, ESR1, MSX1, MAL, CRISP3, CXCL14, WISP2, SCGB2A1, PTCH1, CRNN, SFRP4, APOD, CFD, SCGB1D2 |
| GSE39001  (GPL6244) | CDKN2A, SYCP2, SMC1B, MCM2, MIR15A, DTL, HBA2///HBA1, FAM111B, STIL, ASF1B, MYBL2, EXO1, FOXM1, KIF2C, E2F7, IRF1, IQGAP3, CLSPN, NUSAP1, CDC6, MELK, MCM5, ICAM1, CDH3, ASPM, TYMS, ORC1, ECT2, AURKA, NUF2, UHRF1, NUP210, TPX2, HIST2H3D///HIST2H3A///HIST2H3C, CDC45, EFNA1, NEK2, RFC4, CDK1, KIF14, MEST, CENPO///ADCY3, RNF212, CENPF, TCF19///TCF19, TK1, TOP2A, BRIP1, PRC1, KIF20A///CDC23, PLK1, XRCC2, MCM4, WDR76, UBE2T, CELSR3, CCNB2, TIMELESS, ARHGAP11A, CDT1, CDCA5, KIAA1524, KIF18A, PLAU, HIST2H4B///HIST2H4A, ATAD2, POLQ, ANLN, BAK1, ZNF367, CDC20, MLF1, CDC7, HIST2H4A, HIST2H4B, UBE2S, CEP55, NCAPH, FANCI, RNASEH2A, PRR11, CASC5, KIAA0101, FAM72A///FAM72D, UBE2C, CKS2, KIFC1, MKI67, HIST1H3B, NCAPG2, CDC25C, RPL39L, KIF4A, E2F8, HIST1H2BH, FOXRED2, FANCA, HIST1H3G, DEPDC1, TICRR, SPAG5///SPAG5, CCNB1, HIST1H3H, SDC4, CDCA2, RRM2, C19orf48, HIST1H3H///HIST1H2AI, NUP62CL, LMNB1, CDCA8, CENPK, KIF11, ORC6, GINS2, FAM72A///FAM72D///FAM72B, CENPI, MCM8, PTTG3P///PTTG1, CENPA, DLGAP5, KIF23, LCORL///NCAPG, MCM7, BLM, EZH2, BUB1, GINS4, RAD51AP1, ZYG11A, NETO2, CIT, KIF15, PLK4, RAD51, HIST1H3I, HENMT1, SEP3, KPNA2, TACC3, GINS1, CDK2, WDHD1, RAD54L, HIST1H1B, MCM6, HIST1H2BJ, C17orf53, CDC25B, PODXL2, DNMT1, RFC5, CHEK1, IL32, TTK, LIG1, PRIM1, HIST2H2AA4///HIST2H2AC///HIST2H2AA3, PAK6///BUB1B, OTUD7A, SMC4, PKMYT1, FAM20C, HELLS, KNTC1, HIST1H2BO, MCM3, POLE2, CKAP2L, CHAF1A, C9orf84, SGOL1, PCNA-AS1///PCNA, LSR, CKS1B, SOX30, BIRC5, DSN1, TOPBP1, HJURP, BRCA2, ARHGAP11B, EPHB2, KNSTRN, TRIP13, C7orf13, NDC80, LOC642846///DDX12P///DDX11, LOC642846///DDX12P///DDX11///LOC642846///DDX12P, OIP5, MCM10, LOC100131860, GAS2L3, NCAPD2, CHAF1B, CCNF, HIST1H1C, POLA2, HLTF, GTSE1, STMN1, TMEM106C, DHFRL1///DHFR, AURKB, HIST1H4I, POLD1, CENPL, LOC642846///DDX12P///DDX11///LOC642846///DDX12P///DDX11, KIF22, POLE, CENPE, CDKN2C | NDN, ARHGAP10, SLC25A27, GAB1, SNORD114-26, AOC3, LRRFIP1, CXorf36, ABLIM3, NPR2, SOCS2, HSPB6, FRMD4B, ZNF404, NUDT6///FGF2, ZBTB16, MSRB3, GPRASP1, CYP4F22, CORIN, ZNF426, SLC47A1, LONRF1, CD248, AVPR1A, FGF14, ADCYAP1R1, METTL24, PLN, PAIP2B, DLC1, AIF1L, TNXB///TNXA, TRPS1, KATNAL1, STON1-GTF2A1L///STON1///GTF2A1L, SLC16A6, TBX3, C3orf67, SDPR, PPM1K, KAT2B, PGBD5, SPNS2, ZNF208, GPR22, USP53, MEG3, ACADL, SPG20, ULK4, IGFBP6, SASH1, GREB1L, DYNC2H1, PPFIA2, KCNJ3, TSC22D3, WISP2, CLDN8, CLEC1A, YOD1, STAC, LOC100131826, CC2D2A, ZNF493, ANKRD35, ARHGAP20, LDOC1, KCND2, ABCA5, RGS13, SYTL5, KLHL3, CA5B, VGLL3, APLF, BCO2, RGN, OLFML3, TCP11L2, DIAPH2, NRG1, FAM19A2, CSMD3, ALDH1A3, MOP-1, SLC2A13, ZNF429, CCBE1, GSTA4, CMAHP, TM4SF18, HPGDS, GSTM2, TMEM55A, NEGR1, LINC00312, KDR, IL1RL1, FAM13C, MYLK, MGC24103, TCEAL1, TCEAL4///TCEAL4, PAPPA-AS1///PAPPA, TACR1, SYTL4, KLF6, ADAMTS5, PDGFRB, PLA2G4D, ATF3, SPRY1, ZNF675, PROK1, RPTN, SLIT3, AKT3, GNA14, MRGPRF, DNM3, MEDAG, KLHDC1, BNC2, FHL5, THSD7A, TPTEP1, AGPAT9, OMD, TRPC6, CPEB2, CD34, BEX4, ZNF43, FBLN1, EPHA5, LSAMP, F10, PLCB4, CPQ, PCDHB4, HOPX, SELP, CTTNBP2, ITGBL1, NAALAD2, ECM2, RECK, NDNF, TEK, OSR2, PXK, RGS7BP, ZNF254, SSPN, ZFHX4, CFD, RBP7, GEM, DIO3, FXYD1, FMOD, NRN1, LRRK2, COL21A1, GNG11, CYYR1, FAM171B, ZNF91, PRRX1, EBF1, ZFPM2, PMP22, AQP1, PDZRN3, ARMCX1, EDN3, CYBRD1, KCNJ15, GPR133, LMOD1, MASP1, ABCD2, RBM24, NAP1L2, SULT1B1, SHC3, FAM3D, PRICKLE2, SCNN1G, PTGDS, ZNF385D, NR4A1, JUN, JAM2, MS4A2, LINC01140, RGS2, RGS22, RND3///RND3, CAB39L, MYCT1, PPAP2B, CADPS, JAZF1, NOSTRIN, ZEB2///ZEB2, ALDH1A2, AREG, RUNX1T1///RUNX1T1, MIR145, PDE1A, PRLR, NR4A3, ZNF626, PRKG1, PCDH18, TSTD2///TMOD1///TMOD1, CTGF, GRIA3, LPAR1, KLF4, ZEB1, GUCY1A2, HTR2B, SEMA3E, MICU3, RFTN2, DMKN, IL1R1, LDB2, ZNF415, LY75-CD302///CD302///CD302, SEMA3D, TFPI, CDA, ASPA, GPX3, GULP1, PDE5A, ADAMTS19, ABCC9, NOVA1, CPED1, PAPSS2, CALCRL, MITF, SOX5, EPHA3, RPS6KA6, DDR2, ABCA6, MFAP4, LRFN5, ELTD1, DSEL, FLRT2, PRSS23, MAMDC2, MAPK10, RDH12, ENDOU, TRPC1, CRISPLD2, PKHD1L1, RNF180, KGFLP2///KGFLP1///FGF7, MYZAP///GCOM1///POLR2M, DPT, RORB, SGCE, SYNPO2, SLC27A6, ANK2, FHL1, ABCG2, ME1, CXCL12, KGFLP1///FGF7, EGR1, SLC16A9, TSPAN7, ANKRD31, DCLK1, FBN1, LRCH2, NPY1R, CYP2B7P, SPARCL1, RGS1, SNORD113-3, ITGA8, FGF10, ADAMTS1, ANKFN1, KCNIP4, GREB1, AR, EDNRA, ABCA8, CDR1, PAMR1, TGFBR3, ABI3BP, SULT1C4, EDNRB, KRT78, NT5E, PDK4, NAP1L3, ACSS3, PDGFD, ARHGAP28, EMCN, FGF7, BBOX1, FLG, FAM25G///FAM25C///FAM25A, EYA1, CGNL1, LYVE1, LAMA2, SNORD114-3, OGN, KLK12, DUSP1, SH3BGRL2, LCE3D, HPSE2, GALNT5, IGF1, FOS, SOSTDC1, GYS2, THSD4, SNORD114-2, PGR, C1QTNF7, GSTM5, EREG, LIFR, TRHDE-AS1///TRHDE, COL14A1, MMRN1, SLC18A2, CWH43, SCEL, CYR61, HPGD, TPRG1, PI15, SNORD113-4, IGFBP5, FOSB///FOSB, CNFN, LOC441178, FABP4, FMO2, UPK1A, CRCT1, EPGN, PPP1R3C, MUC21, KRT4, SBSN, APOD, ALOX12, KRTDAP, MAL, SPINK5, CRNN, SPINK7, TMPRSS11B, DSG1, CRISP3 |
| GSE52903 | MMP12, SYCP2, SMC1B, MMP1, CDKN2A, SPP1, CXCL10, IDO1, TPX2, DTL, FAM111B, NUF2, CCL18///CCL18, CDC6, TOP2A, MELK, CENPF, ASPM, ANLN, AIM2, DLGAP5, STIL, CDK1, CLSPN, KIAA0101, NUSAP1, EXO1, FOXM1, MMP3, KIF2C, KIF4A, KIF14, CCNB2, ECT2, CASC5, MCM2, CDKN3, NTS, ARHGAP11A, KIF11, PRC1, UBE2T, CLDN1, ASF1B, MIR15A, BRIP1, CXCL8, E2F7, MKI67, TYMS, CDH3, CENPI, MMP13, NEK2, KIF23, POLQ, AURKA, CEP55, KIAA1524, CENPK, WDHD1, CXCL9, MYBL2, CCNE2, FANCI, KIF20A///CDC23, APOBEC3A_B///APOBEC3B, LCORL///NCAPG, MMP10, TTK, LAMC2, RFC4, PBK, APOL1, ITGB6, BUB1, GBP5, CCNB1, PLK1, CDC7, DEPDC1, PAK6///BUB1B, CHI3L1, GINS1, HBB, IQGAP3, MCM4, FAM72A///FAM72D, TDRD6///PLA2G7, ABCA13, HIST1H3F, RRM2, RAD51AP1, KIF18A, CLSPN, HIST1H3B, SPAG5///SPAG5, DSG2, WDR76, KRT17, MEST, TICRR, CKS2, MCM5, PTTG3P///PTTG1, SHCBP1, CDCA2, LMNB1, ORC1, HORMAD1, HELLS, KIFC1, ORC6, KIF15, PLK4, CXCL11, CDC45, PLOD2, KNTC1, NCAPG2, NETO2, PLAT, DIAPH3, FAM72A///FAM72D///FAM72B, XRCC2, ESCO2, TIMELESS, CHEK1, CXCL2///CXCL1, EGLN3, FCGR3B///FCGR3A, RPL39L, ATAD2, CDC20, KPNA2, KPNA2, MMP9, PLAU, POLE2, SMC4, UBD, ALG1L, DNA2, FAM72A///FAM72D///FAM72B, NCAPH, PRR11, UHRF1, FAM72A///FAM72D///FAM72B, MCM8, BRCA2, GEN1, UBD, CENPO///ADCY3, EZH2, LAMP3, MOCOS, TK1, HIST1H1B, HIST2H3D///HIST2H3A///HIST2H3C, ICAM1, KRT17, UBE2C, CENPE, IFI30, NUP210, OAS3, RAD51, TRIM59, HCAR2///HCAR3, HIST1H2AB, TCF19///TCF19, TCF19///TCF19, BLM, HBA2///HBA1, HBA2///HBA1, IRF1, TCF19///TCF19, CENPW, KIFC1, NDC80, SGOL1, USP41///USP18, SKA3///MRPL57, SPC25, ATAD5, BRCA1, SLFN13, STMN1, TFRC, CMC2///CENPN, FANCA, IL1B, MEI1///MEI1, CA9, CKS1B, PCNA-AS1///PCNA, TDRD9, HIST1H3G, HIST2H3D///HIST2H3A///HIST2H3C, HIST2H3D///HIST2H3A///HIST2H3C, HIST2H4A, HIST2H4B, SLC2A1, TRIP13, C3orf52, RNASEH2A, CKS1B, HIST1H2BH, MCM6, RNF212, CD274, CKAP2, MCM10, NEIL3, PARP14, CDCA5, CXCL13, FCGR3B///FCGR3A, GMNN, ARHGAP11B, CDCA8, FEN1, GAS2L3, LIPH, RACGAP1, CKAP2L, DEPDC1B, GINS2, HIST1H2BF, HIST2H4B///HIST2H4A, HIST2H4B///HIST2H4A, IGHG4///IGHA2, UBE2S, ADAMDEC1, APOC1, CDC25C, CDK2, DSN1, HIST1H3H///HIST1H2AI, HJURP, RDH10, TAP1///PSMB8-AS1, TAP1///PSMB8-AS1, TAP1///PSMB8-AS1, CENPA, HIST1H3H, HMMR, IL1A, LOC1720, MCM3, MCM7, NEFH, TOPBP1, WARS, ZWINT, GINS4, MSL3P1, RAD54L, STRIP2, TACC3, DHFRL1///DHFR, FANCD2, LAMB3, NEDD4L, TNFSF10, ZYG11A, HIST1H3I, MYO3A, RIBC2, CA2, CDT1, DHFRL1///DHFR, KIAA0101, PRIM1, DTX3L///PARP9, E2F8, IL12RB2, MAD2L1, PSMB9, PSMB9, PSMB9, CCDC88C, HENMT1, OTUD7A, CCNA2, CENPU, HLTF, IFI6, OAS2, OCIAD2, RBL1, TMEM171, AQP9, CHAF1B, GGH, LY6K, NRIP3, SLC6A10P///SLC6A8, TMEM194A, ZNF367, AURKB, CCNF, CIT, EFNA1, ITGA3, UBE2S, BAK1, C1orf112, HIST1H2BJ, HIST1H3J, MGME1///MGME1, IL32, KIF4B///KIF4A, NQO1, OAS1, OIP5, STAT1 | CRISP3, SPINK7, TMPRSS11B, DSG1, CRNN, MAL, ALOX12, APOD, MUC21, CRCT1, MUC21, SFRP4, MUC21, SPINK5, KRTDAP, CNFN, SBSN, PPP1R3C, SPRR3, OGN, FMO2, KRT4, LOC441178, LOC441178, UPK1A, HPGD, IGFBP5, COL14A1, GSTM5, CRYAB, PGR, KRT1, TPRG1, LIFR, CWH43, HPSE2, THSD4, C1QTNF7, TMPRSS11E, ABCA8, TMPRSS11E, IGF1, TMPRSS11A, EPGN, FOSB///FOSB, PAMR1, SCEL, SLIT2, SPRR1A, SNORD113-4, LYVE1, TGFBR3, TRHDE-AS1///TRHDE, FAM25G///FAM25C///FAM25A, FAM25G///FAM25C///FAM25A, FAM25G///FAM25C///FAM25A, KLK12, CGNL1, SH3BGRL2, EMCN, ESR1, A2ML1, CYR61, SLC18A2, MMRN1, TGM3, AR, KRT13, DUSP1, LAMA2, SYNPO2, GYS2, SPARCL1, ACSS3, FGF10, KRT78, MIR99AHG, FOS, PDE5A, PDK4, ABI3BP, LCE3D, SFRP1, ABCG2, ITGA8, IL36A, DPT, EMP1, MFAP4, NOVA1, OLFML1, SNORD114-2, SPRR2E, ABCA6, DCN, ANK2, MAMDC2, PDGFD, PRSS3///PRSS2, RHCG, TSPAN7, GREB1, LRCH2, PRLR, EYA1, PKHD1L1, TRPC1, FHL1, KLK13, MUC15, NAP1L3, OSR2, CPED1, CTSK, EDNRA, FGF7, SVEP1, TNXB///TNXA, TNXB///TNXA, CXCL14, SDR9C7, C7, CXCL12, RORB, TSTD2///TMOD1///TMOD1, SNORD114-3, MITF, SNORD116-9///SNORD116-8///SNORD116-7///SNORD116-5///SNORD116-3///SNORD116-1, SNORD116-9///SNORD116-8///SNORD116-7///SNORD116-5///SNORD116-3///SNORD116-1, ANKFN1, MIR145, SNORD116-9///SNORD116-8///SNORD116-7///SNORD116-6///SNORD116-5///SNORD116-4///SNORD116-3///SNORD116-2, SULT1C4, ENDOU, EREG, BBOX1, GPX3, MAPK10, RUNX1T1///RUNX1T1, SERPINF1, SGCE, SNORD116-1, SLC16A9, SNORD116-9///SNORD116-8///SNORD116-7///SNORD116-5///SNORD116-3///SNORD116-1, SNORD116-9///SNORD116-8///SNORD116-7///SNORD116-5///SNORD116-3///SNORD116-1, GULP1, LRFN5, TMEM47, EPHA3, LDB2, ARHGAP28, CYBRD1, LY75-CD302///CD302///CD302, NT5E, DCLK1, MYH11, PTN, LOC100288114///ZNF667-AS1, SLC5A8, SNORD116-21, FLG, SPRR1B, TNXB///TNXA, AQP1, CRISPLD2, FLRT2, PRSS3P2///PRSS3///PRSS2, PTGER2, RDH12, CFD, GJA1, RNF180, ENPP1, GJB6, GUCY1A2, CD34, JAZF1, PCDH18, EDNRB, MGP, PDZRN3, SHC3, CAB39L, CCDC80, LPAR1, JAM2, ZNF737, ALDH1A2, GPR133, KCNIP4, PCDHB4, PDE1A, SNORD113-3, CDR1, DEPTOR, BCHE, CDA, FRZB, IL1R1, MASP1, PI15, PMP22, PREX2, RGS7BP, ABCA9, MPPED2, PGM5P2///PGM5, PTGDS, SNORD64///PWAR5, CTGF, DPP4, LMOD1, MEG3, MICU3, MYZAP///GCOM1///POLR2M, PRICKLE2, ASPA, DKK1, IVL, NOSTRIN, PDGFRA, SLC5A1, ZEB2///ZEB2, CYP1B1, FIBIN, NAP1L2, RGS22, SCGB2A1, SOSTDC1, ARMCX1, CILP, EPHA7, FBN1, ZFHX4, ZNF91, ABCC9, FXYD1, SOX5, ANO4, COL21A1, HAND2-AS1, TNXB///TNXA, DDR2, EGR1, FAT4, MAOB, NDNF, PTCH1, RFTN2, KIT, RPS6KA6, ZNF483, ZNF626, EBF1, NPY1R, PGM5P2///PGM5, PRELP, RBM24, DMKN, MRGPRF, PPAP2B, ANKRD31, OMD, PGM5, SNORD116-6, ZNF415, DSEL, SPON1, TFPI, ADAMTS1, ATP6V1C2///PDIA6, FAM171B, TIMP3, ZNF385D, FBLN1, PTGIS, FAM13C, NR4A1, SLC27A6, THSD7A, CALCRL, DACH1, DIO3, F10, GNG11, LDOC1, MIR675///H19, MUM1L1, PRUNE2, TMEM132C, ADAMTS19, ADAMTS5, AKT3, CLDN17, GSTA4, GSTM2, KGFLP1///FGF7, ME1, PI16, ZNF254, HOPX, METTL24, PRRX1, SHISA6, SLC7A2, AGPAT9, ELTD1, FBLN5, FMOD, EPS8, GEM, KLF4, KLK11, LUM, NAALAD2, ANKRD35, BNIPL, CYYR1, FAM3D, PDGFRB, PLN, RBPMS, AREG, BNC2, GRIA3, HTR2B, ITGBL1, LPHN3, MYCT1, PRKG1, PRSS23, RHOJ, XG, PLCB4, PWAR6, SNORD116-14, SPRR2F, ZEB1, AMY2B///AMY2A///AMY1C///AMY1B///AMY1A, CPQ, CRABP2, FAM189A2, GLT8D2, LINC01140, PARM1, SNORD116-24, CTTNBP2, DIO2, FGF14, KLK10, PXK, RNF150, CNTN4, EMX2, FERMT2, IL18, KLHDC1, LMO3, MPDZ, OLFML3, PCP4, RECK, ZFPM2, ABCA10, AOX1, LRRK2, SCGB1D2, SELP, SEPP1, SNORD116-22///SNORD116-21///SNORD116-20///SNORD116-18///SNORD116-17///SNORD116-16///SNORD116-15///SNORD116-14///SNORD116-19///SNRPN, SNORD116-22///SNORD116-21///SNORD116-20///SNORD116-18///SNORD116-17///SNORD116-16///SNORD116-15///SNORD116-14///SNORD116-19///SNRPN, SORBS1, SSPN, TSPYL5, CC2D2A, CPXM1, DDR2, HSPB8, ISLR, KGFLP2///KGFLP1///FGF7, KGFLP2///KGFLP1///FGF7, KGFLP2///KGFLP1///FGF7, LONRF2, MMP2, NCAM1, NLGN1, PPL, RBP7, ZBTB16, NBEA, PCDHB5, PODN, SLIT3, CACNA1D, CNN1, FGF9, ITM2A, MS4A2, MYLK, SNORD116-20, ZNF43, AMY2B///AMY2A///AMY1C///AMY1B///AMY1A, AMY2B///AMY2A///AMY1C///AMY1B///AMY1A, AMY2B///AMY2A///AMY1C///AMY1B///AMY1A, CPA3, ECM2, KLK6, MCOLN3, PDE8B, PLTP, SCN7A, SPRY1, ZNF493, CPEB2, EDN3, MGC24103, PAM, SLC24A3, SNORD116-15, SULT2B1, TENC1, LRRC17, LTBP1, RBMS3, RGN, SEMA3D, SNCAIP, C8orf4, CACHD1, CCL15-CCL14///CCL15///CCL14, CYP4F22, FAXDC2, MATN2, PIK3R1, SEMA3E, TACR1, WISP2, ZNF676, HSPB6, NRN1, PDE3A, RPTN, ZNF521, ZNF626///ZNF93///ZNF253, BEX4, CLIC3, RGS2, RGS5, SNORD116-23, TIMP2, ZNF429, CCBE1, DNM3, NDRG2, PTPRD, SCNN1G, SMPDL3A, CAV1, CXorf36, ITGA9, MOXD1, NR4A3, NCCRP1, RAET1E, SASH1, SYTL4, TEK, TPTEP1, AASS, CNTN1, DAPL1, PCDHB15, SLC16A7, VIPR2, FLJ38379, LAMB2, PCSK5, RGS1, SNORD116-4///SNRPN, TCEAL1, ABCB1, CADPS, ECM1///ECM1, FHL5, IGFBP6, PAPPA-AS1///PAPPA, PCOLCE, SLC47A1, SPRR2G, TGFB3, ARHGAP20, CLU, CSRNP3, F13A1, NR3C2, PRSS27, PRUNE2, SCNN1B, SDPR |
| GSE63514 | CDKN2A, CTHRC1, ZIC2, ELAVL2, SYCP2, MMP12, AIM2, INHBA, TCAM1P, NUP210, HS6ST2, GREM1, IFI44L, CD163, FST, GREM1, CA9, DTL, KIF14, HS6ST2, APOC1, HOXC6, LHX2, SYNGR3, CXCL8, MLF1, SULF1, SPP1, POLQ, TSEN54, FN1, WDR72, FST, ABCA17P, HIST1H3H, CENPN, COCH, KLHDC7B, PLA2G7, CXCL9, FN1, ANKRD36BP2, MMP9, ATAD2, FBXO5, ADAMDEC1, MCM10, DNA2, INHBA, MTHFD1L, DDIAS, COL8A1, BRIP1, E2F7, APOC1, MIR155HG, FNDC3B, TMSB15A, DEPDC1, C18orf54, C3orf70, ZFR2, BMP8B, NETO2, WDHD1, KIF14, MLF1, KIAA1524, MS4A7, PLOD2, ASPM, TNFSF13B, NUF2, IQCG, C18orf54, CCDC88A, WDR66, SPAG5, ATAD2, RFC4, CXCL11, GBP5, FAM72A///FAM72D///FAM72B///FAM72C, NTS, C18orf54, FNDC3B, POSTN, CHI3L1, CDC6, FOXD1, FNDC3B, WDHD1, TNFAIP6, KIF23, HENMT1, RSAD2, ZWILCH, RSAD2, HELLS, TMEM194A, SULF1, PLAUR, IRX2, KIF15, FOXM1, FANCA, SMC4, CASC5, INSIG1, CDKN2A, CXCR4, GNG2, CDC7, CENPE, CENPQ, KNTC1, CXCL10, HMMR, SLC16A1, MYO3A, COL8A2, DTL, NEFH, IFI44, CENPO, SLC7A7, MERTK, TRIP13, KIF4A, PLK4, CDK1, WDHD1, C5orf34, LINC01305, MS4A4A, SP110, FAM26F, EIF5A2, UHRF1, LIN9, LOC375196, HPS3, CENPI, SNX10, RBL1, OIP5, LRR1, RMI2, ANLN, ALCAM, POLE2, CEP128, SLC16A1, ENO2, MLIP, CENPK, MTFR2, HLTF, MELK, C1orf112, LSG1, IGKC, LAMA4, SMC2, CXCL11, CSE1L, CCNE2, PRIM1, LINC00925, HOXD10, CENPF, BACH2, PLK4, ADAM12, CYP27B1, C9orf84, STAT1, ECT2, RASSF4, TAGLN, SLC16A1, STAT1, IGK///IGKC, CDK1, CENPA, PARPBP, CD274, MAP4K1, RSRC1, DEPDC1, TBC1D30, SIX4, PEX13, MSL2, IFI44, NUP210, TMEFF1, OVOS2, BUB1, NFATC1, GAS2L3, CDC25B, ZYG11A, NEK2, CDCA7, KRT17, CDC25C, CENPN, EPSTI1, ANKRD36BP2, WDR5B, TICRR, MCM2, CABLES2, MSRB3, CTLA4, MX2, GAS2L3, KLHL7, NRIP3, MFI2, MKI67, ENTPD1, SMYD2, APOBEC3B, MAP7D2, FAM69A, ZNF367, KIF2C, SFMBT2, BAG2, HOXD11, MYBL2, GINS2, PKMYT1, CDCA2, ASPM, THBS1, LY96, SOAT1, TMPO, MICB, DEPDC1B, TOP2A, FN1, LOC100288693, PXDN, FAM26F, CDKN3, RAD54L, NDC80, UBXN7, CMPK2, IGKV1-5, AURKA, KPNA1, ABHD10, MMP3, FAP, HOXD13, FAM102B, SGOL2, TOP2A, TXNRD1, SELL, RAB3IP, DMXL2, CEP55, DCLRE1B, POLQ, CELSR3, NEXN, PIGX, IFIT3, SLAMF8, HP1BP3, FLJ32255, THBS1, CDK1, CDC6, MCM8, TNFAIP6, BBOX1-AS1, ANP32E, CHEK1, IL24, MARCKSL1, U2SURP, PRKDC, OASL, MRAS, RAD51AP1, FADS1, PIK3AP1, DLGAP5, CDKN3, FILIP1L, KIAA1804, REEP1, KIFC1, SHCBP1, USP18, TOPBP1, TTK, PTP4A3, C1QB, TIPIN, NUP62CL, CD163, STK4, E2F1, INPP4A, VEGFA, MRC1, ANKRD32, CSPG5, TRIO, ESCO2, BUB1B, ZGRF1, HERC5, NEDD4L, FCHSD2, RSRC1, NUSAP1, TIMP3, TMEM206, EXO1, ISG15, RGS1, PRTFDC1, FN1, MKI67, MYNN, DDX6, ASXL1, FAM26F, OSGIN2, IL17RB, SDCBP2-AS1, WDR76, TMEM68, SGOL2, ZMAT3, HELLS, FOXF2, B3GALTL, TDO2, HSPBAP1, PIK3CA, STAT1, GSPT1, SLAMF8, C4orf46, MIR181A2HG, CHEK1, PDK3, MTF2, CENPJ, SLFN11, GABPB1, RGS1, PIF1, SLC25A28, ANGEL2, CCDC150, COL5A2, ASXL1, GINS1, CENPF, ASPN, GEMIN2, C3orf52, NCAPG2, DEPDC1, HMMR, SLC25A37, PRC1, IGK///IGKC, CCDC71L, ADAMTS2, FANCI, TMEM38B, MUC4, MIS18BP1, BIRC5, GBP1, PCOLCE2, RECQL4, USP13, SLC20A1, IQGAP3, RNASEH2B, RFC3, TAZ, FNDC3B, DDX58, ARMC8, DONSON, TRAM2, MCM3, VASH2, FNDC3B, HAUS6, RMI1, KIF11, FAM111B, GBP1, STRIP2, FAM64A, MIPEPP3, NUSAP1, MCOLN2, MCM4, ERAP1, CEP85, FAM89A, STIL, DBF4, TMEM44, MOCOS, PLSCR1, ANLN, KCNS3, BLM, PDE10A, NETO2, RAD1, FBXO5, NCEH1, MIRLET7D, ASAP1, CDC45, MKI67, DSCC1, DLEU2, HELLS, IL7R, CHML, COL5A2, SLC16A1, ASXL1, NCAPG, VCAM1, AMIGO2, ZNF519, NDE1, IGL, COQ10A, ABL2, RRM2, FPR3, LOC100509694///HDAC10///MAPK12, TRIM59, CXCL13, MCPH1, GPR19, CHEK1, IGSF6, UNC5CL, CPNE8, FST, CSF2RB, HAUS5, CEP152, PDIA5, MTF2, CDCA5, RIBC2, SGK494, ETV5, SULF1, NASP, RNF213, IFI30, FILIP1L, CXCR4, DLEU2, TCF19, VMP1, PXDC1, FXR1, EID3, LAYN, KLHL24, C21orf91, CDK2, AURKA, ZDHHC23, CCNB1, EZH2, C17orf75 | CRNN, CRISP3, TMPRSS11B, SPINK7, MAL, FAM3D, SPINK5, ENDOU, CLCA4, UPK1A, SPRR3, THSD4, KRT4, EDN3, RDH12, LCE3D, CRCT1, SCNN1B, PIGR, ALOX12, KRT1, SBSN, KRTDAP, CLIC3, TFF3, PPP1R3C, CRISP2, TCN1, GREB1, TPRG1, CYSRT1, SLURP1, BPIFB1, CNFN, BBOX1, KLK12, ZG16B, C1orf177, S100A12, CEACAM7, FCGBP, FMO2, KRT13, A2ML1, RHCG, ZBED2, KLK12, KLK11, RPTN, THSD4, HPGD, SFTA2, SOSTDC1, KLK12, MUC5B, PRSS27, RBM20, CYP4F22, HPGD, PHYHIP, ALOX15B, KLK13, GALNT5, IVL, NSG1, DAPL1, TMPRSS11E, SCNN1G, GPX3, ESR1, AR, IL1R2, AR, FMO2, BNIPL, SCEL, CGNL1, DMKN, HPGD, CYP3A5, DSG1, PRSS3, TGM3, CWH43, FLG, SCEL, CST6, ALOX12B, CYP4B1, GCOM1, TGM1, NSG1, CRABP2, CRYAB, TMPRSS11D, SPRR1A, HOPX, CXCL14, TF, GYS2, WNT4, CWH43, IL1R2, SLC5A1, GJB6, LCN2, SMIM5, SULT2B1, VSIG10L, KRT2, APOD, SAMD5, TMEM184A, CSTB, EREG, LOC100505650, PADI1, C2orf54, AGFG2, KRT4, TGM5, SPAG17, MMP28, SPRR1B, EMP1, CITED4, FUT3, NDRG4, PGR, GBP6, GGT6, NIPAL4, TMEM45B, CFTR, FLG-AS1, CDA, SLC16A9, TTC22, CYP2C18, C10orf99, HOXB8, GJB2, P2RY2, KLK8, THSD4, DUOX1, PROM1, LOC441178, KLK10, KLK6, CXCR2, MAB21L3, MUC5AC, LOC100130476, ACPP, LYNX1, MMP28, MALL, SH3PXD2A-AS1, CYP2B7P, KLK10, MPPED2, ATP6V1C2, MUC15, ZNF300P1, CCDC64B, GPX3, TBX3, LOC102659288, TF, RHOD, GGT2///GGTLC3///GGTLC2///GGT3P///GGT1, SCGB1D2, AKR1B10, PLA2G4F, GCNT3, TP53AIP1, PRSS3, DPP4, EPHX2, CLDN10, CTTNBP2, LOC100507487, SLC15A1, IL18, FAM3B, HBA2///HBA1, CXCL14, TMEM40, ANKRD35, ZNF506, C15orf52, SERPINB1, PRSS3P2///PRSS1, HOPX, SLC46A2, THSD4, PRSS3///PRSS2///PRSS1, HSPB8, TMEM229B, HP, MAML3, PTK6, PRAC1, ALKBH7, MMP28, TM7SF2, ATP6V1C2, GLTP, ST6GALNAC1, ZNF844, C10orf99, DGAT2, AKR1C3, CFD, SCGB3A1, SLPI, PNLIPRP3, CYP4X1, FAM189A2, EPHA1, LOR, TTC39A, SCGB2A2, VSIG2, MGLL, GATSL3///TBC1D10A, RGS12, CAPN5, BCO2, MUC5AC, KLK7, PI3, TOM1, GDPD3, TMEM132C, MSX1, FAM83A, ECM1, CYP2C18, KLK7, PITX1, FAM46B, A2ML1, ACPP, CYP3A5, KRT78, SERPINB13, ZNF667-AS1, SH3RF2, TP53AIP1, FAM86A, TRIM16, PDGFD, TFF1, DEFB1, ZBTB7C, C6orf132, CXCL14, TMEM45B, SNORA68, CERS3, ANPEP, KLK13, SLC24A3, KLF4, DMBT1, SH3BGRL2, SLC7A2, MGLL, HBA2///HBA1, LOC441178, C1orf116, SLC5A1, PTCRA, FCER1A, C1orf116, SPNS2, CHMP6, ACOX2, PDZK1IP1, SLC16A6, YIF1B, SPRR2C, FGFBP1, C5orf66-AS1, PSCA, STRA6, PKDCC, KLB, C4BPA, SPON1, CYP3A5, FAM83A, ALAD, SPTLC3, EPS8L1, SLC16A6, GBP6, PCP4L1, ZSCAN18, SYNGR1, SHROOM3, THSD4, SORBS2, AGPAT6, GGTLC1, SLC34A2, C2orf88, RAET1E, EMP1, PLLP, LOC100287024, SLC27A6, DEPTOR, ARRB1, IL36RN, EPB41L3, TP53I3, TBX3, NUDT18, BEX4, C15orf59, DCUN1D3, PAMR1, HINT3, RSPH1, ZNF667-AS1, GGT1, EVPL, KCNK7, NDN, HPR///HP, KRT78, IL20RA, MIR31HG, EPB41L3, ZNF91, QSOX1, ALDH1A2, CCDC88B, ZNF415, AHNAK, PI3, HBA2///HBA1, SERPINB1, CRYL1, PKIA, RAPGEFL1, ECHDC2, BTBD11, LOC100505650, RNF128, EPS8L1, CAPS, SLC16A7, IGFBP6, PPL, OTUB2, SCEL, MPZL2, CD177, CRYM, EMP1, SLC26A4, PLA2G6, KLF8, MUC6, C6orf1, CDRT4, ACPP, ZNF750, ZNF439, C9orf16, MYL5, SYNGR1, HPGD, SPON1, ANKRD22, MUC15, SHROOM3, TINCR, LDOC1, MAST4, GULP1, SBSPON, BAIAP2, TST, ARRB1, EPB41L3, C17orf59, GGT1, AADACL2, SDK2, ZNF667, PEX11G, TMEM79, FUT6, CPA6///CPB1, FAM20A, ALKBH7, RHOD, ADAMTSL4, PEG3, ALDH3B2, SLC24A3, ANXA9, IL17C, SORBS2, ELOVL6, FAM63A, KALRN, VSIG2, MPST, ZNF91, B3GNT8, RARG, CD24, EHF, BCL2L15, PTPRCAP, DUOX1, RAB7B, TMPRSS13, CSF3, CCND1, ANO10, TMEM120A, FUT6, C6orf132, FLJ23867///QSOX1 |
| GSE67522 | CDKN2A, SPP1, CELSR3, CXCL8, CEL, KRT7, MMP12, UBE2C, CDC20, ZIC2, KLHL35, ALG1L, KIF2C, ASF1B, TCAM1P, APOC1, AURKA, PNCK, CCNB2, NUSAP1, CA9, CDT1, KIF20A, UHRF1, CXCL10, CDC45, AURKA, CDCA5, MELK, MMP9, CDKN2A, POLQ, CENPF, AURKB, RAD54L, TACC3, TROAP, ASPM, HENMT1, IDO1, SYCP2, MELK, KIFC1, SPP1, PRC1, SLC16A3, TRIP13, C17orf53, GINS2, BIRC5, STIL, TOP2A, CEP55, PLEKHG4, MMP11, NEK2, PAQR4, CKAP2L, SYCP2, DSG2, C16orf59, RYR1, UBE2T, NUP210, DLGAP5, CCNF, KRT19, NCAPG, RFC4, CDCA8, CXCL1, FOXM1, EXO1, KNTC1, TIMELESS, CDKN3, TTK, KIAA0101, CENPN, AUNIP, RAD51AP1, CBS, CXCL9, PTTG1, AIM2, CENPA, ECT2, NEFH, YBX2, OIP5, LAMP3, RMI2, WDR66, DLL3, ARHGEF16, FANCI, E2F7, MCM4, ENO2, C1orf112, GSDMB, FANCD2, ATAD2, ISG15, CHAF1B, CDK1, HJURP, GRP, DLGAP5, RNASEH2A, TYMS, TNFRSF12A, SMC4, RFC4, POC1A, SLC6A10P, PLAU, PTTG3P, GDF15, MICB, TPX2, BUB1, TK1, E2F2, IL32, HMMR, KIF14, FAM64A, RBP1, SALL4, FEN1, RIPPLY3, PODXL2, STAT1, PARP14, RNF212, TMEM44, OLR1, MCM10, DHDH, PIF1, TMEM44, ANLN, MTHFD1L, STMN1, KIF23, PLAUR, MOCOS, SIX4, ABCC5, SUV39H1, ADAM8, PLXNA1, IL32, LRP8, TOPBP1, CHTF18, IFI30, RECQL4, CEP85, TYMP, NUP62CL, GBP5, SPC24, CENPM, KIF15, HES6, SLC2A1, RACGAP1, BRCA1, ALG1L, STAT1, TUBB3, NRIP3, NMB, PLOD2, TTYH3, MCM5, MCM7, CDCA2, LHX2, GMNN, CCNA2, MTFP1, DONSON, WDR90, WARS, NUSAP1, C10orf35, FOXM1, DEPDC1, TYMSOS, MCM6, CDC25C, REC8, IRF7, BCL2L12, WARS, YEATS2, SLC6A8, CDC25A, PLK4, NCAPD2, KRTCAP3, CDCA4, PLA2G7, ZWINT, CKS1B, CKS2, MCM4, HMMR, PBK, CCNE1, ITGAX, PCSK9, POLD1, BCL2L12, FANCG, GAS2L3, LIG1, RYR1, DDX11, ELF4, BORA, ISG20, SLC9A1, SMC1B, TFRC, SEP3, TEAD4, TRIB3, GSDMB, ITPKA, CHAF1A, VEGFA, CDK2, CDC25B, ADAM19, IRF1, POLA2, CKS2, KIF22, POLE2, KIF11, FBXO5, IL17RB, LYPD1, C16orf93, SMIM22, HLTF, EXOC3L4, HOXD1, SAPCD2, PLOD2, MARVELD3, BRCA1, KIF11, SUGCT, EFHD2, CABLES2, SLC15A3, SKA3, RSRC1, TTF2, STEAP3, SNORD99, OAS2, SLC52A1, HELZ2, SLC51A, TICRR, HOXC8, HAUS8, DNMT1, BAIAP2L1, C19orf48, APOL1, APOL2, PHF19, SLC26A6, TMEM132A, DSN1, FAM24B, SLAMF8, MCM3, RUNX3, APOL2, PCNA, TMEM171, CYP27B1, MMP12, PSMB8, FAM72D, RASEF, PRIM1, FADS1, TBC1D31, KRT86, MCM8, ENTPD6, MARVELD3, EDARADD, GAD1, NCEH1, RFC5, CKS1B, LRCH4, RPL39L, FANCB, IFNAR2, PARPBP, GPC2, ABCB9, C17orf96, ARTN, LRR1, DDIAS, BCAS4, SYNE4, HAUS7, SLX4, NCBP2, TINAGL1, OAS3, PDLIM7, SEC61A1, SNORA61, KBTBD12, HOXC9, LMNB2, MRPL2, CDCA7, BRICD5, HRK, LEMD1, SYCE2, RNF213, CEP131, SLC35G2, HES4, ERVMER34-1, CENPK, BCAS4, SLC29A2, SLC20A1, B4GALT4, DSG2, SLC25A19, RPP25, NDC80, SNX8, RPS6KA1, PLXNA3, WDR34, HELLS, RFC3, MAD2L2, CHEK1, DCBLD2, SAPCD2, RPA3, PAFAH1B3, POLE, WDHD1, MARVELD3, SKP2, EME1, HSPA6, TMEM106C, DMRTA2, BRI3BP, LAMP3, FOXD2-AS1, E2F3, PRSS53, C3orf52, RFWD3, HMGB2, NUP155, ACOT9, TMC6, PTPN6, BRI3BP, WRAP73, CDKN2D, OCIAD2, SDHAP2, SLC12A8, REC8, VRK1, TAP1, TRIM11, FANCE, ORC6, LSR, KIF24, SMC2, EFCAB11, TNFRSF10B, ACTL6A, KIF20B, DIDO1, CCDC77, HOXC4, CCDC14, PLK1, PARP12, PUS1, PCED1A, ABCA7, NR2C2AP, FAM50A, MB, B4GALT5, RMI1, TMEM194A, PDXK, CEP104, MUTYH, MIS18A, SMG5, FEN1, HAUS8, BST2, AK2, DCBLD2, CENPA, EIF4EBP1, PCK2, FBXL18, PRIM1, INCENP, HAUS8, NPL, LYN | BOC, LINC00936, SEMA5A, OTUB2, ULK4, NAP1L3, ABCG4, FBXO3, RPL21P28, LANCL1, PDCD4, OMD, THEM5, NAALAD2, DIXDC1, CACHD1, EMCN, SNRK, PDE1A, LHFP, SDC2, FAM174A, ZNF135, RPL14, GPIHBP1, STXBP6, SLC25A25, GSPT2, SDR9C7, TRIM13, GPR63, RBM20, SRI, ROR1, TANC1, TSC22D3, ENTPD3, DYNLT3, HNMT, PLK2, EDNRB, ZFP36L2, DEGS2, FGF2, ZNF586, C1QTNF7, EIF4B, OSBPL1A, PDGFD, SMARCD3, EBPL, IL20RA, HLF, CAT, RNU4ATAC, CREB3L2, GFRA2, ZNF586, FAM110B, NPR2, NRXN2, CX3CR1, BTF3, EPB41L4A-AS1, HAND2-AS1, RPL9, CCM2L, PALM, DCUN1D3, ID2, KLHDC1, SNRK, PTN, UST, CD300LG, FBLN5, EEF1B2, ISOC1, JMY, TEK, MUSTN1, BTG2, SCUBE2, LTBP4, RELN, REM1, RPL15, DDAH2, S1PR3, C4orf3, NAAA, SYNGR1, EMX2, TREX2, LAMA2, BEND5, SPTLC3, PTGIS, MEOX1, ZNF667-AS1, DLG2, SC5D, LIPK, OSBPL5, NOV, ARC, SAMD5, HIGD1A, SNHG7, VWA5A, PRCP, CSDC2, FAXDC2, RPS29, RFTN2, SORBS2, CD34, ANKRD35, ACVRL1, FAM110D, CPQ, SDR9C7, SH3D19, NELL2, PLLP, IDE, DNALI1, THSD4, DKK3, LANCL1, OSBPL5, FXYD1, PDE7B, CLEC14A, ZNF426, IGFBP4, LYVE1, COL14A1, SPINK9, IRS2, VAMP2, PRICKLE2, GEM, LMO2, FAM162B, ADRA2A, MITF, RPS23, SLC9A9, GEM, KCNK7, RNF125, PCDH18, ANKRD29, GPR1, ARSF, SSPN, VAT1, FBXL5, ACYP2, PALM, ZBTB16, FAM25G, CKMT2, ABCA12, NDNF, EML1, HIGD1A, MUC15, TEK, DEPTOR, CCSER2, PCSK5, HSPB2, AQP1, ENDOD1, ECSCR, C10orf32, FAM25C, TP53INP2, RAB5A, FAM198B, SPIN2B, METTL7A, RPL9, PPAP2A, RPRM, ADAMTS1, WFDC5, PROS1, UNC93A, CES1, SNHG7, QPCT, SPRY2, LRRC20, ALDH2, SLIT3, PRUNE2, JAZF1, FAM134B, ARL5A, ZFP36, KAT2B, ZBED2, PDGFD, TST, NDP, ZNF415, PTGS1, FMOD, GPR124, IGF1, MAOB, LPAR1, FLRT2, CCSER2, RECK, RORA, TMTC1, CPEB2, GSDMA, PIK3R1, TNFRSF19, IL6ST, CCDC178, HOXA10, TEX101, HSPB8, RGS5, RGS5, GATSL3, TMEM98, MATN2, CITED4, ARL4A, TMOD1, SBSPON, SERPINF1, SPTLC3, HOXA13, CA4, LDB2, CPQ, OLFML3, CYYR1, CITED2, FETUB, ANKRD22, PRSS2, APCDD1, OSR1, ALOXE3, SMO, NOSTRIN, UBL3, DDIT4L, ATP1B2, RUNDC3B, LPAR6, SLIT2, AHNAK, SORBS2, GHR, PRRX1, SNHG8, SRPX, PLCD1, ARMCX1, SASH1, ARL4A, IL17D, FXYD1, EPHB6, SH3BGRL, SERPINA12, SNX21, RNF150, PPAP2B, OLFML1, MED27, LCE1A, SASH1, IVL, KCNK7, MAPK10, MGLL, TUBB2A, TPRG1, EFNB3, ABCA8, AADACL2, TBX3, SOSTDC1, WNT5A, SLIT3, BEX4, DEPTOR, SPNS2, PID1, PCDH18, ZSCAN18, SNORD3A, FAM189A2, CD207, CFAP58, KLK9, POF1B, CIDEA, TGM5, RERG, COX7A1, GAS7, JAM2, SLC5A1, MYLK, RAI2, FRZB, PDGFRA, PDE1A, SNORD3C, ACPP, SEP5, PDGFRA, LGALSL, YOD1, SCARA5, SLC24A3, EPB41L3, LCE2C, SFTA2, PNLIPRP3, MT4, AGPAT9, DACH1, SLC39A2, KANK1, RHOB, WFDC12, GNG11, PEG3, SLC7A2, RNASE4, FHL1, TM7SF2, DCN, CYBRD1, CPED1, ELOVL4, MSX1, COBL, SNORD13, CTSG, TCEAL2, PGM5, ZNF91, TSPAN7, EGR1, CDA, ACOX2, BCHE, DNASE1L3, GSTA4, C2orf40, GSTM3, SDPR, HSPB6, GSTM5, DCN, PDE5A, NSG1, CYP2C18, RASD1, ITM2A, LDOC1, ANKRD35, CYBRD1, C15orf59, TNFAIP8L3, SPRY1, SNORD3D, HAL, SH3BGRL2, LCE1C, SPRY1, ENDOU, KLF4, IL36RN, LCE1B, DEGS1, TNRC6A, PARM1, HPGD, SPON1, EDN3, EMX2, DSC1, MFAP4, TGM5, FLG2, CFD, C6orf15, AR, CWH43, ATP6V1C2, CXCL14, NDN, GJA1, CCL21, CDSN, HOPX, KLF4, SPARCL1, BBOX1, DPT, FAM107A, GPX3, KLK5, DSC1, PGM5, ALDH1A2, SPARCL1, CLEC3B, ARG1, PGM5, MASP1, IGFBP5, KLK12, CLDN17, LY6G6C, ALOX12B, PLA2G4D, KRT78, FAM3D, MAMDC2, CRYAB, VSIG8, ALOX12, IGFBP5, CGNL1, DMKN, TMPRSS11B, DUSP1, LCE2B, PLAC9, FCER1A, PRSS3, ABI3BP, ACKR1, PAMR1, TFF3, CXCL12, MMRN1, RDH12, PI16, LCE3E, ASPRV1, KLK13, SFRP4, HOPX, SFRP1, FAM25A, FAM25A, KRT2, FOS, FOSB, WISP2, UPK1A, CYP4F22, PPP1R3C, CCL14, MAL, PTGDS, MAL, RPTN, LCE3D, APOD, SLURP1, SPINK7, TGM3, CRNN, LOR |
