# Supplementary material for: Bioinformatics and in vitro experimental analyses identify the selective therapeutic potential of interferon gamma and apigenin against cervical squamous cell carcinoma and adenocarcinoma
Source: Oncotarget. 2017 May 2;8(28):46145–62. doi: 10.18632/oncotarget.17574 (PMC5542256; doi:10.18632/oncotarget.17574)
Supplement: Supplementary file 3 [file oncotarget-08-46145-s003.docx]

**Table S3. The differentially expressed genes (DEGs) of cervical adenocarcinoma (AC) and squamous cell carcinoma (SCC) obtained from the dataset GSE39001 (GPL201).**

| **Histological subtype** | **Upregulated genes** | **Downregulated genes** |
| --- | --- | --- |
| SCC | NME1, IGSF3, CYC1, CIB1, SNRPB, IFIT3, USP14, VDAC1, MX2, ATG5, MARCKS, RAD21, PKM, BLM, KIF22, LSM2, HSPA4L, ALG6, OAS2, PRPF4, ABCC5, ITGB2, MKI67, CCZ1B///CCZ1, PFDN2, PSMA3, EIF4EBP1, CORO1A, NCBP1, UBE2L3, PRDX1, SLC20A1, ASNS, PAFAH1B3, MTHFD2, XPNPEP1, CAPNS1, NUP155, UMPS, DCK, ALDOA, PTPRF, HNRNPU, SNX10, ATG3, PSMD2, HIST2H2AA4///HIST2H2AA3, PLAUR, GPSM2, PDXK, LAMC2, PHB, PRMT1, NPC1, TFAP2A, HMMR, CSNK1D, IL10RB, GZMA, BTN3A3, MSH6, MIR3658///UCK2, CD2, SCO2, KIF23, MTF2, SYNGR3, RAD23A, RRM1, CCNA2, CDK2, HTATIP2, HNRNPAB, WHSC1, UBE2I, ATP1A1, CCL18, TFDP2, DAZAP2, LOC101928634///SENP3-EIF4A1///SNORD10///SNORA48///SNORA67///EIF4A1, CD164, NUP62, LMNB1, SLC25A5, ELF3, PSMA1, MTHFD1, PSMD1, TNNT1, PCCB, USP1, USP18, ZNF207, HIST1H1C, GZMB, HLA-G, MIR1282///HYPK///SERF2, YWHAZ, FCGR3B, H1FX, PAWR, LIG1, HSPA1L///HSPA1B///HSPA1A, KRT8, PSMD8, TUBG1, PIK3CA, RUVBL1, PPP1CA, SLC2A1, CDKN2C, PSMD11, GJB3, PRKCI, ACTR2, SNRPD1, MMP9, HLA-DRB1, GDE1, ARPC5, HMGB1, HLA-B, PSMA7, TMX1, NIT2, HOXC6, ACOT7, ABCA1, CAPG, PSMB2, SPAG5, ZNF148, CD14, FCER1G, PSAP, ELF4, CEBPG, PRIM1, STAT1, MICB, INHBA, KIF2C, LDHA, DVL3, ICAM1, ALOX5AP, CDC45, ITGB4, LOX, KIF15, SLAMF8, IMPA2, VAMP8, YWHAH, DDOST, DHCR24, SLC16A1, TAPBP, GAPDH, PDCD10, SHMT2, RC3H2, PSMD12, BARD1, DDX39A, ACTL6A, ASS1, SLC6A8, CKMT1A///CKMT1B, MCM3, STAT3, TYMP, CDC25B, EFNA1, IRF7, H2BFS, ISG20, DEK, POLE2, SOX4, POU2AF1, ATP1B3, BUB1, LAP3, SDC1, RPN2, BST2, NMI, CTPS1, DSG2, GNB1, LAPTM4B, RAN, RANBP1, NUP107, TAGLN2, CCNE2, SMC1A, AP2M1, MSH2, DNMT1, SLBP, RNASEH2A, GBP1, LOC101060835///LOC100996809///HLA-DRB5///HLA-DRB4///HLA-DRB1///HLA-DQB1, CLDND1, EIF2S2, AP2S1, POLE3, ACTB, RAD51AP1, PLP2, WARS, TRIP13, P4HB, STMN1, HAT1, TK1, STIL, PNP, KPNA2, CTSC, HN1, BTN3A2, GCH1, LAMB3, HNRNPF, C1QA, FZD6, IRF1, NCK1, PSMC3, RFC3, PLAU, HSPA2, HPRT1, LAPTM5, GMPS, PSMB8, DHX15, RFC5, TIMELESS, HBB, CTSS, CHI3L1, AURKA, LSM4, KRT19, FEN1, IFI16, PDZK1IP1, IDH2, H2AFZ, KIF11, HCAR3, TACC3, ENO1, SCD, F11R, ILF2, CCT2, CENPE, ATP2A2, MCM6, BIRC5, SLC38A1, MRPS12, TGFBI, SELT, CA2, HSP90AB1, MX1, LOC101060835///LOC100996809///HLA-DRB4///HLA-DRB3///HLA-DRB1///HLA-DQB1, OAS3, NEK2, KIF4A, TTK, ADAMDEC1, MMP3, OAS1, NDC80, PBK, TRIM29, EZH2, PCNA, TNFSF10, SDC4, IFI30///PIK3R2, NCAPG, RYR1, C1QB, GINS2, IFI27, ID1, IDO1, GGH, CKS1B, CDC7, LOC100509457///HLA-DQA2///HLA-DQA1, TOPBP1, IL32, CDH3, MAD2L1, RACGAP1, PSMB9, HLTF, MEST, PLAT, ISG15, BUB1B, SNORD14D///SNORD14C///HSPA8, IFI44L, HBA2///HBA1, S100P, GMNN, KIF20A, SPP1, UBE2S, PLSCR1, SLC35F6///CENPA, TPX2, CXCL11, PTTG1, HLA-DRA, CCNB2, GPR87, UBE2C, MCM5, SYCP2, AIM2, APOBEC3B, LAMP3, ZWINT, PLOD2, TOP2A, CXCL13, MCM2, CDK1, CKS2, SMC4, CDKN2A, CDKN3, CDC20, RFC4, PRC1, MMP1, CXCL9, CXCL10, KRT17///JUP, UBD///GABBR1, TYMS, NUSAP1, RRM2, MMP12 | SCGB1D2, CFD, APOD, SCGB2A1, CRISP3, TFF3, PTCH1, CRNN, SFRP4, WISP2, SCGB2A2, CXCL14, MSX1, ESR1, MAL, DEFB1, CRCT1, ACTG1P4///AMY2B///AMY2A///AMY1C///AMY1B///AMY1A, PROM1, PTGDS, PCP4, CYR61, FHL1, ID4, LOC100506718///FLRT2, NDN, OSR2, IGF1, BCHE, ALOX12, SPINK5, ABCA8, PPP1R3C, FCGBP, ANK2, EDN3, TGFBR3, RAI2, NR2F1, LDB2, DKK3, SFRP1, COX7A1, SRPX, LRRC17, CYP2B7P///CYP2B6, MPPED2, PDGFRA, FOS, NDP, ADRA2A, MYLK, LOC101928635///ALDH1A2, FOSB, DUSP1, CILP, HTR2B, LOC100506558///MATN2, NAP1L3, C4BPA, CLDN10, CYP1B1, MITF, IL1R1, EXOSC7///CLEC3B, EDNRA, FXYD1, DKK1, SLIT2, EGR1, SLPI, FBN1, LTBP1, GNG11, RBPMS, NCAM1, JAM2, SPARCL1, GRIA2, PDGFD, TRH, TGFB1I1, SSPN, KCNJ15, TNS1, ISLR, ADAMTS1, DIO2, ITM2A, , SLC24A3, CLU, RHOB, NT5E, SORL1, SGCE, IGFBP6, NOVA1, ECM2, SCNN1B, S100A4, LEPROT///LEPR, GJA1, IL20RA, SORD, FGF9, ADAMTS5, SOCS2, PLPP3, SLC18A2, DPP4, CIRBP, FAM107A, PRSS23, SOX17, PCSK5, GATA2, GSTM5, COL16A1, PCOLCE, ALPP, FZD10, FGF13, EFEMP2, TST, TUBA1A, CCL15-CCL14///CCL14, CFTR, SNAI2, RUNX1T1, SLC4A4, FGFR1, IRS1, CRYAB, PAM, TGM2, GHR, IRS2, FOLR1, DKK2, DACH1, PKIG, ABCG2, LOC101926921///DAB2, LMOD1, MINOS1-NBL1///NBL1, LDOC1, BDKRB2, CTSK, CHN2, SERPINF1, DIO3, PLPP1, PRR16, RARRES2, HEPH, CTGF, AQP1, TCEAL1, CDC42EP3, PTGER2, PEBP1, FMOD, EPS8, PECAM1, , CACNA1D, EPHB6, MMP2, CITED2, MAOB, IL1R2, LAMB2, NUCB2, CHST7, COLEC12, KAT2B, SLC5A1, PMP22, ALDH1A3, KCNMA1, KIT, RECK, ADRB2, FBXL7, SCUBE2, CX3CR1, WFDC1, F10, DCLK1, GCNT3, RGS2, BNIP3, EDNRB, HOXA10, KLF4, FRZB, REV3L, ATP1A2, MXRA7, UPK1A, LOC100130872///SPON2, SATB1, ZBTB16, STX18, CRTAP, NME5, QPRT, RRAGD, LAMA2, KLHL3, AKAP12, RCAN1, CNN1, PLTP, GSTA4, PLLP, TCF12, DUSP6, ARHGAP6, CDA, ALDH6A1, SALL1, WFS1, IL33, INHBB, TACC1, SLCO1B3, ZFPM2, PTGIS, ENDOU, RAPGEF3, NR3C2, LAMC3, MAP1A, GALNT6, TPM2, CLIC5, COL18A1, CAMLG, MOAP1, GAS6, , PCDHGA1///PCDHGA2///PCDHGA3///PCDHGA4///PCDHGA5///PCDHGA6///PCDHGA7///PCDHGA9///PCDHGA10///PCDHGA11///PCDHGB1///PCDHGB2///PCDHGB3///PCDHGB5///PCDHGB6///PCDHGB7///PCDHGC4///PCDHGC5///PCDHGA12///PCDHGA8///PCDHGB4///PCDHGC3, WASF3, PHYH, SERPINA5, ENTPD3, BBOX1, PENK, TPST1, NFATC4, PDGFRL, NELL2, DPT, BDH2, SNCAIP, NRCAM, CH25H, PDE4A, BCL2, ASPA, ACPP, PDS5B, CDO1 |
| AC | IRX5, PPIH, SF3B4, ACTL6A, B3GNT3, SYNGR2, CDC25C, ARPC5, PLEK2, TROAP, LDHA, ATP1A1, KCNK1, MARCKS, DTYMK, EZR, PPM1G, CDK2, PRSS8, HLA-A, RALY, HMGN2, PSMB2, HMGB3, BST2, CHAF1B, ALG6, AKR7A3, CTPS1, TXNRD1, MTF2, PSMD8, PSMB3, VRK1, RPA3, RAD21, ENO2, RAN, IRF1, HLA-B, RBBP4, SMPDL3B, ISG20, SIM2, HMGA1, EIF2AK1, XPNPEP1, HNRNPU, SLX1B-SULT1A4///SLX1A-SULT1A3///SULT1A4///SULT1A3, AKAP1, CHAF1A, TSTA3, NUP62, SLC20A1, TMEM2, CCNE2, TRAF4, USH1C, HLA-G, LMNB1, LSM2, SNRPD1, RAD51, SNRPB, OAS3, PAX6, MSH2, CIB1, PHB, POLE3, PDXK, KIF2C, NMI, DCK, MKI67, NUP107, SHMT2, CLDN7, GAPDH, SMS, DHFR, BUB1, KIF22, VDAC1, SLC25A5, PXMP2, MYO10, BLM, ELF4, RAD23A, MYBL2, FAT1, CLIC1, PFKP, PLAUR, CCNA2, IRF7, LAMC2, EIF2S2, ITGA3, SPAG5, CTSS, PLPP2, VILL, CSNK1D, HNRNPAB, DDOST, GATM, USP1, TAPBP, ENO1, MTHFD1, SOX9, RPN2, MLF1, RHOC, PAFAH1B3, TRIP13, CRIP2, HMGB1, TJP3, CKLF-CMTM1///CKLF, F11R, EFNA1, PSMB9, TCP11, ISG15, DEK, MYB, TMPRSS4, MGAT4B, LSR, GNB1, TAGLN2, GUSB, DSG2, KRT19, BACE2, DNALI1, MRPS12, YWHAH, PSMC3, VNN2, MST1R, KPNA2, CENPE, KRT18, RANBP1, DDX39A, ATP2A2, CYBA, CDC45, PDZK1IP1, IDH2, SLBP, ITPR3, HPRT1, POLE2, CDKN2C, PNP, AURKA, PLSCR1, WHSC1, BRCA1, FA2H, ASS1, PRIM1, ITGB3BP, BARD1, PSMB8, SMC1A, LAPTM4B, TUBG1, DNMT1, RAD51AP1, STMN1, LLGL2, HNRNPF, LIG1, ANXA3, IFI30///PIK3R2, NDC80, STIL, MCM3, KIF15, ELF3, RNASEH2A, GMDS, HN1, TTK, CKS1B, DHX15, MYRF, TIMELESS, ITGB4, RFC5, ATP1B1, HLTF, TOPBP1, IFI27, SLC44A4, LAMB3, ILF2, LSM4, APOBEC3B, PLS1, NQO1, FEN1, CDC25B, SDC4, MCM6, CA9, KIF11, TK1, NCAPG, IL32, BIRC5, H2AFZ, HOXC10, UBE2S, RACGAP1, HBA2///HBA1, PTTG1, RFC3, SLC38A1, TACC3, EZH2, CTSE, KIF4A, CDC7, ABCC3, ERBB3, SLC35F6///CENPA, TPX2, KRT8, KIF20A, GINS2, SMC4, AKR1C3, PCNA, MAD2L1, EPCAM, BUB1B, CLDN3, PBK, MCM2, TSPAN1, CCNB2, TFF1, MMP12, KRT17///JUP, GABRP, HOXC6, CKS2, GMNN, MEST, CDKN2A, NEK2, RFC4, CDKN3, CDK1, MCM5, UBE2C, MUC1, SYCP2, ZWINT, PRC1, KRT7, TOP2A, S100P, CDC20, MSLN, TYMS, RRM2, LGALS4, NUSAP1 | KRT13, KRT14, SPRR3, SFRP4, CFD, SPRR1B, CRNN, KRT6A, MAL, CSTA, SCGB1D2, SPINK5, APOD, CXCL14, CRCT1, WISP2, MSX1, DKK3, SPRR1A, LOC100506718///FLRT2, FHL1, SNAI2, SFRP1, PTGDS, PTCH1, RHCG, SCGB2A2, ESR1, PDGFRA, ITM2A, SPRR2B, MYLK, NDN, IGF1, GJA1, PCP4, ANK2, SRPX, FBN1, CTSK, KRT6C///KRT6B///KRT6A, LOC100506558///MATN2, LTBP1, SPARCL1, PPP1R3C, ID4, LDB2, ABCA8, BCHE, EDN3, OSR2, CRYAB, DEFB1, PRNP, MPPED2, COX7A1, AKR1B10, NR2F1, COL16A1, SERPINF1, EFS, IGFBP6, RAI2, FABP4, IVL, DKK1, CYP1B1, THBD, CYR61, KCNJ15, BEX3, C1S, ADRA2A, ADRB2, NAP1L3, FGFBP1, HTR2B, SLC24A3, IL1R1, MAF, EDNRA, SCNN1B, JAM2, CSRP2, NDP, EMP1, EXOSC7///CLEC3B, CILP, BNIP3, CYP2B7P///CYP2B6, FBLN1, GPNMB, ADIRF, RGS1, GNG11, FRZB, PLPP3, ECM2, ISLR, S100A4, TGFBR3, PECAM1, SOCS2, SEPP1, GHR, LOC101926921///DAB2, SSPN, CDC42EP3, ME1, GRIA2, FZD10, MAOB, MMP2, DUSP1, ALDH1A3, PLPP1, PDGFD, ADAMTS1, FXYD1, RUNX1T1, COL3A1, LPAR6, LEPROT///LEPR, KCNMA1, SERPING1, NCAM1, CFH, TGM2, PMP22, SLC18A2, CX3CR1, KAT2B, TUBA1A, TRH, IRS1, THBS2, DIO2, NELL2, CCND2, SORD, GSTM5, FYN, KIT, MITF, PLTP, GSTA4, HEPH, SGCE, COL1A2, NSG1, BASP1, ADAMTS5, ECI2, NOVA1, FGF13, COLEC12, CCL15-CCL14///CCL14, DPYSL3, LMOD1, EFEMP2, COL6A3, PRSS23, PCOLCE, LHFP, CIRBP, RECK, MAFB, HTRA1, ANOS1, PRR16, GATA2, DKK2, PTGIS, SNCAIP, PCSK5, FOSB, DCLK1, BCL2, EPHB6, LDOC1, ABCG2, SCUBE2, TNS1, CPA3, DIO3, LAMA2, PDGFRL, CHST7, TCEAL1, STC1, IL33, MXRA7, KLHL3, PDLIM2, FAM107A, FMOD, COL5A1, FBXL7, DPT, CH25H, CRTAP, TPST1, RAB31, FGFR1, RCBTB2, MRC2, EGR1, CAV1, MINOS1-NBL1///NBL1, EDNRB, ROBO1, TCEAL9, PKIG, PCDHGA1///PCDHGA2///PCDHGA3///PCDHGA4///PCDHGA5///PCDHGA6///PCDHGA7///PCDHGA9///PCDHGA10///PCDHGA11///PCDHGB1///PCDHGB2///PCDHGB3///PCDHGB5///PCDHGB6///PCDHGB7///PCDHGC4///PCDHGC5///PCDHGA12///PCDHGA8///PCDHGB4///PCDHGC3, LMO2, GAS7, PLSCR4, TCF4, CNN1, CDA, SLCO2A1, ZNF185, CITED2, ACAT1, F10, TAF7, ZBTB16, PTGER2, BACE1, WIPF1, LAMA4, HLA-DQB1, SERPINE2, RHOQ, PDGFRB, ARHGAP6, TGFB3, BDKRB2, CELF2, ADGRL4, SNORA11E///SNORA11D///MAGED4///MAGED4B, F13A1, RCAN2, PLAGL1, SPOCK1, BCL6, ATP1A2, FBLN2, SALL1, PRKAR2B, PEBP1, A2M, ENDOU, ZEB2, SATB1, NOTCH2, PENK, TACC1, LAMC3, SMARCD3, RGN, WFS1, DACH1, WNT4, CXCL12, PLA2G4C, TGFB1I1, HSD17B6, LAMB2, CCNG2, DYNLT3, SERPINI1, SSBP2, TRPS1, KAT6B, PDE2A, P2RX5-TAX1BP3///TAX1BP3, GSTM2, GRK5, IL19, SAA2-SAA4///SAA4, MEF2C, MAGI2, GAS6, ARL6IP5, TSPAN7 |
