# Supplementary material for: Bioinformatics and in vitro experimental analyses identify the selective therapeutic potential of interferon gamma and apigenin against cervical squamous cell carcinoma and adenocarcinoma
Source: Oncotarget. 2017 May 2;8(28):46145–62. doi: 10.18632/oncotarget.17574 (PMC5542256; doi:10.18632/oncotarget.17574)
Supplement: Supplementary file 4 [file oncotarget-08-46145-s004.docx]

**Table S4. The differentially expressed genes (DEGs) specific for cervical adenocarcinoma (AC) and/or squamous cell carcinoma (SCC).** The AC-specific, SCC-specific, and common DEGs were prepared from supplementary Table S3.

| **Category** | **Upregulated genes** | **Downregulated genes** |
| --- | --- | --- |
| Specific genes altered in SCC | NME1, IGSF3, CYC1, IFIT3, USP14, MX2, ATG5, PKM, HSPA4L, OAS2, PRPF4, ABCC5, ITGB2, CCZ1B///CCZ1, PFDN2, PSMA3, EIF4EBP1, CORO1A, NCBP1, UBE2L3, PRDX1, ASNS, MTHFD2, CAPNS1, NUP155, UMPS, ALDOA, PTPRF, SNX10, ATG3, PSMD2, HIST2H2AA4///HIST2H2AA3, GPSM2, PRMT1, NPC1, TFAP2A, HMMR, IL10RB, GZMA, BTN3A3, MSH6, MIR3658///UCK2, CD2, SCO2, KIF23, SYNGR3, RRM1, HTATIP2, UBE2I, CCL18, TFDP2, DAZAP2, LOC101928634///SENP3-EIF4A1///SNORD10///SNORA48///SNORA67///EIF4A1, CD164, PSMA1, PSMD1, TNNT1, PCCB, USP18, ZNF207, HIST1H1C, GZMB, MIR1282///HYPK///SERF2, YWHAZ, FCGR3B, H1FX, PAWR, HSPA1L///HSPA1B///HSPA1A, PIK3CA, RUVBL1, PPP1CA, SLC2A1, PSMD11, GJB3, PRKCI, ACTR2, MMP9, HLA-DRB1, GDE1, PSMA7, TMX1, NIT2, ACOT7, ABCA1, CAPG, ZNF148, CD14, FCER1G, PSAP, CEBPG, STAT1, MICB, INHBA, DVL3, ICAM1, ALOX5AP, LOX, SLAMF8, IMPA2, VAMP8, DHCR24, SLC16A1, PDCD10, RC3H2, PSMD12, SLC6A8, CKMT1A///CKMT1B, STAT3, TYMP, H2BFS, SOX4, POU2AF1, ATP1B3, LAP3, SDC1, AP2M1, GBP1, LOC101060835///LOC100996809///HLA-DRB5///HLA-DRB4///HLA-DRB1///HLA-DQB1, CLDND1, AP2S1, ACTB, PLP2, WARS, P4HB, HAT1, CTSC, BTN3A2, GCH1, C1QA, FZD6, NCK1, PLAU, HSPA2, LAPTM5, GMPS, HBB, CHI3L1, IFI16, HCAR3, SCD, CCT2, TGFBI, SELT, CA2, HSP90AB1, MX1, LOC101060835///LOC100996809///HLA-DRB4///HLA-DRB3///HLA-DRB1///HLA-DQB1, ADAMDEC1, MMP3, OAS1, TRIM29, TNFSF10, RYR1, C1QB, ID1, IDO1, GGH, LOC100509457///HLA-DQA2///HLA-DQA1, CDH3, PLAT, SNORD14D///SNORD14C///HSPA8, IFI44L, SPP1, CXCL11, HLA-DRA, GPR87, AIM2, LAMP3, PLOD2, CXCL13, MMP1, CXCL9, CXCL10, UBD///GABBR1 | SCGB2A1, CRISP3, TFF3, ACTG1P4///AMY2B///AMY2A///AMY1C///AMY1B///AMY1A, PROM1, ALOX12, FCGBP, LRRC17, FOS, LOC101928635///ALDH1A2, C4BPA, CLDN10, SLIT2, SLPI, RBPMS, CLU, RHOB, NT5E, SORL1, IL20RA, FGF9, DPP4, SOX17, ALPP, TST, CFTR, SLC4A4, PAM, IRS2, FOLR1, CHN2, RARRES2, CTGF, AQP1, EPS8, CACNA1D, IL1R2, NUCB2, SLC5A1, WFDC1, GCNT3, RGS2, HOXA10, KLF4, REV3L, UPK1A, LOC100130872///SPON2, STX18, NME5, QPRT, RRAGD, AKAP12, RCAN1, PLLP, TCF12, DUSP6, ALDH6A1, INHBB, SLCO1B3, ZFPM2, RAPGEF3, NR3C2, MAP1A, GALNT6, TPM2, CLIC5, COL18A1, CAMLG, MOAP1, WASF3, PHYH, SERPINA5, ENTPD3, BBOX1, NFATC4, BDH2, NRCAM, PDE4A, ASPA, ACPP, PDS5B, CDO1 |
| Specific genes altered in AC | IRX5, PPIH, SF3B4, B3GNT3, SYNGR2, CDC25C, PLEK2, TROAP, KCNK1, DTYMK, EZR, PPM1G, PRSS8, HLA-A, RALY, HMGN2, HMGB3, CHAF1B, AKR7A3, TXNRD1, PSMB3, VRK1, RPA3, ENO2, RBBP4, SMPDL3B, SIM2, HMGA1, EIF2AK1, SLX1B-SULT1A4///SLX1A-SULT1A3///SULT1A4///SULT1A3, AKAP1, CHAF1A, TSTA3, TMEM2, TRAF4, USH1C, RAD51, PAX6, CLDN7, SMS, DHFR, PXMP2, MYO10, MYBL2, FAT1, CLIC1, PFKP, ITGA3, PLPP2, VILL, GATM, SOX9, MLF1, RHOC, CRIP2, TJP3, CKLF-CMTM1///CKLF, TCP11, MYB, TMPRSS4, MGAT4B, LSR, GUSB, BACE2, DNALI1, VNN2, MST1R, KRT18, CYBA, ITPR3, BRCA1, FA2H, ITGB3BP, LLGL2, ANXA3, GMDS, MYRF, ATP1B1, SLC44A4, PLS1, NQO1, CA9, HOXC10, CTSE, ABCC3, ERBB3, AKR1C3, EPCAM, CLDN3, TSPAN1, TFF1, GABRP, MUC1, KRT7, MSLN, LGALS4 | KRT13, KRT14, SPRR3, SPRR1B, KRT6A, CSTA, SPRR1A, RHCG, SPRR2B, KRT6C///KRT6B///KRT6A, PRNP, AKR1B10, EFS, FABP4, IVL, THBD, BEX3, C1S, FGFBP1, MAF, CSRP2, EMP1, FBLN1, GPNMB, ADIRF, RGS1, SEPP1, ME1, COL3A1, LPAR6, SERPING1, CFH, THBS2, CCND2, FYN, COL1A2, NSG1, BASP1, ECI2, DPYSL3, COL6A3, LHFP, MAFB, HTRA1, ANOS1, CPA3, STC1, PDLIM2, COL5A1, RAB31, RCBTB2, MRC2, CAV1, ROBO1, TCEAL9, LMO2, GAS7, PLSCR4, TCF4, SLCO2A1, ZNF185, ACAT1, TAF7, BACE1, WIPF1, LAMA4, HLA-DQB1, SERPINE2, RHOQ, PDGFRB, TGFB3, CELF2, ADGRL4, SNORA11E///SNORA11D///MAGED4///MAGED4B, F13A1, RCAN2, PLAGL1, SPOCK1, BCL6, FBLN2, PRKAR2B, A2M, ZEB2, NOTCH2, SMARCD3, RGN, WNT4, CXCL12, PLA2G4C, HSD17B6, CCNG2, DYNLT3, SERPINI1, SSBP2, TRPS1, KAT6B, PDE2A, P2RX5-TAX1BP3///TAX1BP3, GSTM2, GRK5, IL19, SAA2-SAA4///SAA4, MEF2C, MAGI2, ARL6IP5, TSPAN7 |
| Common genes altered in SCC and AC | CIB1, SNRPB, VDAC1, MARCKS, RAD21, BLM, KIF22, LSM2, ALG6, MKI67, SLC20A1, PAFAH1B3, XPNPEP1, DCK, HNRNPU, PLAUR, PDXK, LAMC2, PHB, CSNK1D, MTF2, RAD23A, CCNA2, CDK2, HNRNPAB, WHSC1, ATP1A1, NUP62, LMNB1, SLC25A5, ELF3, MTHFD1, USP1, HLA-G, LIG1, KRT8, PSMD8, TUBG1, CDKN2C, SNRPD1, ARPC5, HMGB1, HLA-B, HOXC6, PSMB2, SPAG5, ELF4, PRIM1, KIF2C, LDHA, CDC45, ITGB4, KIF15, YWHAH, DDOST, TAPBP, GAPDH, SHMT2, BARD1, DDX39A, ACTL6A, ASS1, MCM3, CDC25B, EFNA1, IRF7, ISG20, DEK, POLE2, BUB1, RPN2, BST2, NMI, CTPS1, DSG2, GNB1, LAPTM4B, RAN, RANBP1, NUP107, TAGLN2, CCNE2, SMC1A, MSH2, DNMT1, SLBP, RNASEH2A, EIF2S2, POLE3, RAD51AP1, TRIP13, STMN1, TK1, STIL, PNP, KPNA2, HN1, LAMB3, HNRNPF, IRF1, PSMC3, RFC3, HPRT1, PSMB8, DHX15, RFC5, TIMELESS, CTSS, AURKA, LSM4, KRT19, FEN1, PDZK1IP1, IDH2, H2AFZ, KIF11, TACC3, ENO1, F11R, ILF2, CENPE, ATP2A2, MCM6, BIRC5, SLC38A1, MRPS12, OAS3, NEK2, KIF4A, TTK, NDC80, PBK, EZH2, PCNA, SDC4, IFI30///PIK3R2, NCAPG, GINS2, IFI27, CKS1B, CDC7, TOPBP1, IL32, MAD2L1, RACGAP1, PSMB9, HLTF, MEST, ISG15, BUB1B, HBA2///HBA1, S100P, GMNN, KIF20A, UBE2S, PLSCR1, SLC35F6///CENPA, TPX2, PTTG1, CCNB2, UBE2C, MCM5, SYCP2, APOBEC3B, ZWINT, TOP2A, MCM2, CDK1, CKS2, SMC4, CDKN2A, CDKN3, CDC20, RFC4, PRC1, KRT17///JUP, TYMS, NUSAP1, RRM2, MMP12 | KRT13, KRT14, SPRR3, SPRR1B, KRT6A, CSTA, SPRR1A, RHCG, SPRR2B, KRT6C///KRT6B///KRT6A, PRNP, AKR1B10, EFS, FABP4, IVL, THBD, BEX3, C1S, FGFBP1, MAF, CSRP2, EMP1, FBLN1, GPNMB, ADIRF, RGS1, SEPP1, ME1, COL3A1, LPAR6, SERPING1, CFH, THBS2, CCND2, FYN, COL1A2, NSG1, BASP1, ECI2, DPYSL3, COL6A3, LHFP, MAFB, HTRA1, ANOS1, CPA3, STC1, PDLIM2, COL5A1, RAB31, RCBTB2, MRC2, CAV1, ROBO1, TCEAL9, LMO2, GAS7, PLSCR4, TCF4, SLCO2A1, ZNF185, ACAT1, TAF7, BACE1, WIPF1, LAMA4, HLA-DQB1, SERPINE2, RHOQ, PDGFRB, TGFB3, CELF2, ADGRL4, SNORA11E///SNORA11D///MAGED4///MAGED4B, F13A1, RCAN2, PLAGL1, SPOCK1, BCL6, FBLN2, PRKAR2B, A2M, ZEB2, NOTCH2, SMARCD3, RGN, WNT4, CXCL12, PLA2G4C, HSD17B6, CCNG2, DYNLT3, SERPINI1, SSBP2, TRPS1, KAT6B, PDE2A, P2RX5-TAX1BP3///TAX1BP3, GSTM2, GRK5, IL19, SAA2-SAA4///SAA4, MEF2C, MAGI2, ARL6IP5, TSPAN7 |
