# Supplementary material for: Bioinformatics and in vitro experimental analyses identify the selective therapeutic potential of interferon gamma and apigenin against cervical squamous cell carcinoma and adenocarcinoma
Source: Oncotarget. 2017 May 2;8(28):46145–62. doi: 10.18632/oncotarget.17574 (PMC5542256; doi:10.18632/oncotarget.17574)
Supplement: Supplementary file 5 [file oncotarget-08-46145-s005.docx]

**Table S5. Summary of predicted CMap drugs that reversed the gene expression profiles of SCC and AC.** The differential expressed genes (DEGs) from SCC and AC were used to query the CMap. Only the results of CMap drugs with enrichment score < 0 and *p* value < 0.01 were considered significant. The predicted CMap drugs were ranked according their mean scores. The more negative in mean score indicates the higher possibility of CMap drugs to reverse the DEGs.

| **Cervical cancer subtype** | **Drug name** | **Mean score** |
| --- | --- | --- |
|  |  |  |
| SCC | piperidolate | -0.72 |
|  | Prestwick-559 | -0.719 |
|  | thioguanosine | -0.712 |
|  | rimexolone | -0.71 |
|  | repaglinide | -0.709 |
|  | apigenin | -0.701 |
|  | ronidazole | -0.689 |
|  | sulconazole | -0.677 |
|  | Prestwick-1084 | -0.674 |
|  | omeprazole | -0.674 |
|  | ifenprodil | -0.65 |
|  | clomipramine | -0.646 |
|  | phthalylsulfathiazole | -0.643 |
|  | propafenone | -0.622 |
|  | trifluoperazine | -0.48 |
|  | thioridazine | -0.472 |
|  | chlorpromazine | -0.469 |
|  | trichostatin A | -0.421 |
|  | LY-294002 | -0.346 |
|  | tanespimycin | -0.34 |
| AC | DL-thiorphan | -0.888 |
|  | 8-azaguanine | -0.838 |
|  | rottlerin | -0.807 |
|  | trazodone | -0.791 |
|  | thioguanosine | -0.777 |
|  | milrinone | -0.756 |
|  | estriol | -0.741 |
|  | repaglinide | -0.734 |
|  | omeprazole | -0.732 |
|  | rimexolone | -0.729 |
|  | apigenin | -0.722 |
|  | clomipramine | -0.721 |
|  | sulconazole | -0.718 |
|  | medrysone | -0.703 |
|  | phthalylsulfathiazole | -0.695 |
|  | meticrane | -0.691 |
|  | pargyline | -0.682 |
|  | tyloxapol | -0.681 |
|  | amiodarone | -0.659 |
|  | pyrvinium | -0.628 |
|  | cloperastine | -0.607 |
|  | thioridazine | -0.576 |
|  | vorinostat | -0.562 |
|  | trifluoperazine | -0.557 |
|  | alpha-estradiol | -0.555 |
|  | chlorpromazine | -0.544 |
|  | trichostatin A | -0.541 |
|  | tanespimycin | -0.538 |
|  | LY-294002 | -0.398 |
